# Efficient Chitosan–Ferulic Acid Hydrogel Formation at Neutral pH by a Two-Domain Bacterial Laccase: Mechanistic and Functional Study

**DOI:** 10.3390/polym18172167

**Published:** 2026-09-05

**Authors:** Alexandr Dmitruk, Pavel Oskin, Artem Yushkin, Sergey Alferov, Aleksey Bykov, Olga Ponamoreva

**Affiliations:** 1 Biotechnology Department, Tula State University, Pr. Lenina 92, 300012 Tula, Russia; dmitrukar@gmail.com (A.D.); temayushkin@gmail.com (A.Y.); s.v.alferov@gmail.com (S.A.); 2 Laboratory of Ecological and Medical Biotechnology, Tula State University, Friedrich Engels Street 157, 300012 Tula, Russia; pavelfraj@yandex.ru; 3 Department of Biotechnology, Chemistry, and Standardization, Tver State Technical University, 25 Lenin Avenue, 170023 Tver, Russia; bykovav@yandex.ru

**Keywords:** bacterial laccase, *Streptomyces carpinensis*, chitosan, ferulic acid, cross-linking, hydrogel, reaction mechanism, antioxidant activity

## Abstract

For the first time, for oxidative cross-linking of chitosan with a natural polyphenol, ferulic acid, the so-called small two-domain bacterial laccase, was used. Recombinant *Streptomyces carpinensis* laccase (ScaSL) has been shown to oxidize ferulic acid in neutral and alkaline environments, which is important for subsequent nucleophilic reactions of chitosan with oxidation products. The selected conditions (pH 7.0, molar ratio FA/NH_2_ = 1:10) provide a cross-linking degree of more than 80%, which significantly exceeds the indicators previously achieved using other laccases. Based on the results of quantum chemical modeling and experimental studies using FTIR spectroscopy and XPS, as well as thermogravimetric and differential scanning calorimetry, a new mechanism for laccase-catalyzed cross-linking of chitosan with ferulic acid without the formation of Schiff bases is proposed. The resulting hydrogels have a uniform smooth microstructure, high oxygen permeability, a significant degree of swelling (270%), and stability over a wide pH range. The material neutralizes up to 95% of ABTS^+•^ cation radicals. Quantum chemical analysis within the framework of the Marcus theory has demonstrated that the chitosan–ferulic acid conjugate is superior in antioxidant capacity to ferulic acid dimers, despite the decrease in the matrix element of the bond. Application of two-domain bacterial laccase for modifying chitosan hydrogels with ferulic acid opens the way to the creation of environmentally friendly biomaterials with antioxidant protection.

## 1. Introduction

Laccases (p-benzenediol: oxygen oxidoreductase, p-diphenol oxidase, EC 1.10.3.2) are copper-containing oxidases that catalyze the oxidation of substrates from a wide range of compounds (aromatic phenols and their derivatives, azo compounds, cyano complexes with transition metals, etc.) in combination with the four-electron reduction of molecular oxygen to water. Copper–-containing oxidases are widely distributed in wildlife, from bacteria to higher plants and insects, and are involved in a variety of physiological and ecological processes, including stress responses, melanogenesis, lignification, and degradation of biopolymers [1,2,3,4,5].

The active center of laccases includes four copper atoms contained in three centers: T1 (paramagnetic “blue” center), T2 (paramagnetic “shallow” center) and a two-core cluster T3 (diamagnetic pair). Their coordinated work underlies the unique catalytic mechanism that provides direct four-electron reduction of molecular oxygen to water without the formation of any peroxide intermediates. During catalysis, the substrate is single-electron oxidized in the T1 center, then the electrons are transferred to the T2–T3 cluster, where oxygen is reduced [6].

The ability of laccases to oxidize a wide range of phenolic compounds has led to their active use in biotechnology, including modification of biomaterials, synthesis of biologically active compounds, and degradation of xenobiotics [7,8]. The oxidation of phenylpropanoids, in particular ferulic acid (FA), is of particular interest. It plays an important role in the formation of structural components of plant cell walls and regulation of phytohormonal balance [9,10]. Enzymatic oxidation of FA by laccase leads to the formation of stable phenoxyl radicals capable of dimerization and polymerization with the formation of oligomeric and polymeric products exhibiting antioxidant and antimicrobial activity [11,12,13].

One of the promising applications of laccase oxidation of phenylpropanoids is the natural modification of polysaccharides such as pectins, chitosan, and alginates [14]. These biopolymers have high biocompatibility, biodegradability, and hydrophilicity, which makes them in demand in biomedicine, pharmaceuticals, and the food industry. Modification of polysaccharides by phenylpropanoids proceeds by the mechanism of nucleophilic addition and allows the formation of covalently cross-linked structures due to reactive radicals generated during the oxidation of FA under the action of laccases. The resulting biomaterials form dense films and demonstrate improved performance characteristics [15,16,17,18].

Fungal laccases are most active in an acidic environment (pH 3.5–4.5). Under these conditions, the amino groups of chitosan become protonated and are not capable of nucleophilic addition. Therefore, chitosan modifications are typically carried out at a pH between 6.0 and 7.5 [19,20,21]. In this environment, the activity of fungal laccases is low. In contrast, two-domain (2D) bacterial laccases, also known as small laccases (SLACs), actively oxidize phenolic substrates in both neutral and alkaline conditions, have thermal stability, and are resistant to typical inhibitors of fungal laccases—halides [22]. Recently, there has been an increasing interest in two-domain laccases due to their potential as a green catalyst caused by their unique biochemical properties and the development of genetic engineering techniques for their production [18,23].

The two-domain laccase from the actinobacterium *Streptomyces carpinensis* VKM Ac-1300 (ScaSL) is the first representative of this class that has been characterized. It has an average T1-center redox potential. This recombinant enzyme can be easily produced in a heterologous expression system based on *E. coli*. ScaSL laccase exhibits maximum activity when oxidizing the phenolic substrate 2,6-dimethoxyphenol at a pH of 7.5. It also maintains stability within the pH range of 9–11 and is resistant to classical laccase inhibitors—halides at neutral pH [24]. The purpose of this study is to develop an effective method of enzymatic cross-linking of chitosan with ferulic acid using bacterial laccase ScaSL at neutral pH, to find out the reaction mechanism and evaluate the functional properties of the obtained hydrogels.

## 2. Materials and Methods

The experiments used chitosan with a high degree of purification, degree of deacetylation > 95%, molecular weight 150–200 kDa (BLDpharm, Shanghai, China), ferulic acid, purity 99% (Sigma-Aldrich, St. Louis, Missouri, USA), Tris-HCl buffer solutions (pH 4.5–9.0; 50 mmol/dm^3^) and Britton–Robinson (pH 10.0–11.0; 50 mmol/dm^3^). A 1% aqueous solution of acetic acid was used to convert chitosan to a dissolved state. All reagents used were purchased from DIAM (Moscow, Russia), unless otherwise indicated, and were qualified to be chemically pure and used without additional purification.

Bacterial two-domain laccase isolated from actinobacteria *Streptomyces carpinensis* VKM Ac-1300 (ScaSL) was obtained using a heterologous expression system based on the *Escherichia coli* strain M15 [pREP4] according to the protocol described in [24]. An enzyme solution with a protein concentration of 5 mg/cm^3^ and a specific activity of 17 U/mg was used in the experiments. The amount of 2,2′-azinobis (3-ethyl-benzothiazoline sulfonate) (ABTS) oxidized by the enzyme preparation in one minute at pH 4.5 and room temperature was taken per unit of activity (units).

### 2.1. Modification of Chitosan FA Gels Involving Laccase

All amino groups have been assumed to be in the deacetylated form since the degree of deacetylation of the chitosan used, according to the manufacturer’s specification, exceeds 95%; thus, 1 mole of amino groups accounts for 1 mole of the monomeric unit of the polysaccharide (glucosamine). To select the conditions for chitosan cross-linking, we used a sequential optimization method, as it was the most suitable under the given biochemical limitations. The chitosan modification was performed by varying the pH of the medium and the molar ratio of ferulic acid to chitosan amino groups (n(FA)/n(NH_2_)), while the conditions were selected sequentially. First, an appropriate pH was determined, and then the ratio of reagents was chosen in the optimal medium. For this purpose, a 1.3% chitosan solution was prepared in 1% acetic acid until a homogeneous gel was formed. Then, a buffer solution (Tris-HCl or Britton–Robinson, pH 7.5 to 12) was sequentially added to the resulting gel with a pH value from 4.5 to 9, FA solution (50 mM) in a volume from 0.1 to 0.8 cm^3^ (the ratio of n(FA)/n(NH_2_) varied from 1/2.5 to 1/20) and laccase ScaSL (2.6 units of activity, 130 units/g of chitosan). After thorough mixing, the samples were incubated at room temperature for 72 h in air with constant stirring on an RML-80Pro rotary mixer (JOANLAB, Huzhou, China).

### 2.2. Characteristics of Chitosan Gels

The FTIR spectra of the initial components and modified chitosan samples were recorded using an InfaLUM FT-08 IR spectrometer (Lumex, St. Petersburg, Russia) in the wavelength range of 4000–500 cm^−1^. The dried samples were ground in an agate mortar with spectrally pure KBr (Lumex, St. Petersburg, Russia), after which the resulting mixture was pressed under pressure on a PGR-10 hydraulic press (LabTools, Saint. Petersburg, Russia) until transparent tablets were formed, which were used to register absorption spectra.

XPS spectra were recorded using an upgraded ES—2403 electron spectrometer (SKB AP RAS, St. Petersburg, Russia) equipped with a PHOIBOS 100-5MCD energy analyzer (Specs GmbH, Berlin, Germany) and an MgKa/AlKa XR-50 X-ray source (Specs GmbH, Berlin, Germany). A detailed description of the procedure for recording and processing the spectra is provided in Supplementary Materials (S1).

TG/DSC analysis was performed using a Skz 1053a thermal analyzer (SKZ Industrial, Jinan, China) at a heating rate of 10 °C per minute in a nitrogen atmosphere. The heating range is 30–600 °C.

Scanning electron microscopy was used to examine the morphology of the hydrogel. The samples were first dried in air at room temperature. Then, the dried hydrogel fragments were mounted on aluminum SEM stubs using double-sided conductive carbon tape (Ted Pella, Redding, California USA). Prior to microscopy, a thin (~10 nm) conductive metal coating was applied to the sample surface by magnetic sputtering (C156RS setup, Technoinfo, Moscow, Russia). The survey was carried out using a Hitachi TM-4000 scanning electron microscope (Hitachi, Tokyo, Japan) with a secondary electron detector. The accelerating voltage was 15 kV. The degree of cross-linking was determined by the gravimetric method (by the loss of mass of the cross-linked gel after washing with an acetic acid solution according to the method used for other cross-linked polymers [25,26,27]). The air-dried and suspended gel was mixed with 3% acetic acid for a day, separated by filtration, washed with distilled water and dried to a constant weight. The degree of cross-linking (DC) was calculated by Equation (1):

(1) $$\mathrm{DC}=\frac{m_{G}}{m_{0}}\times 100\%$$

where m_G_ is the mass of the dried gel after treatment with acetic acid, and m_0_ is the initial mass of the dried gel.

The degree of swelling of the hydrogel samples was determined using a gravimetric method. Dry samples were weighed on an analytical balance (m_0_) and then placed in an excess of distilled water at room temperature until equilibrium swelling was reached. After this, the samples were removed from the water and excess liquid was removed from the surface using filter paper, and they were re-weighed (m). The degree of swelling (EWS, %) was then calculated using the following equation:

(2) $$EWS=\frac{{m-m}_{0}}{m_{0}}\times 100\%$$

To determine the oxygen permeability of the film, a modified technique from [28] was used. The oxygen content was measured using an Expert 001 thermoximeter (Econix, Moscow, Russia) in a 20 mL measuring cuvette containing distilled water at 18 °C. A modified chitosan gel used in the study was dried in air for three days. In the control experiment, the cuvette was purged with argon to establish a constant oxygen level of 1.86 ± 0.09 after which an increase in oxygen content was recorded using a Clark oxygen electrode when air entered the cuvette until equilibrium was established. Similar measurements were made using an oxygen electrode on which films of modified chitosan had been placed.

### 2.3. Determination of Modified Films Stability

The stability of the obtained modified chitosan films in various media was evaluated gravimetrically. Samples of modified chitosan gel dried to a constant weight (weight ~20 mg) were placed in 5 cm^3^ of an appropriate medium (distilled water, Britton–Robinson buffer solutions with pH 4.5, 6.0, 7.5, 9.0, 10.5, and 12.0 (50 mM), 95% ethanol, and 3% hydrogen peroxide solution). The samples were kept at room temperature for 24 h, after which they were extracted, washed with distilled water, dried to a constant weight at 60 °C, and weighed. The relative weight loss was calculated using the formula:

(3) $$X=\frac{m_{0}-m}{m_{0}}\times 100\%$$

where X is the relative weight loss, %; m_0_ is the initial mass of the sample; m is the mass after incubation. All measurements were performed in three repetitions; the results are presented with confidence intervals (*p* = 0.95).

### 2.4. Assessment of Antioxidant Activity

Anti-radical activity was determined by the spectrophotometric method using the radical cation ABTS^+•^. The ABTS solution was prepared by oxidizing the ABTS solution (7 mM) with potassium persulfate (2.45 mM) in the dark for 12–16 h. The resulting solution was diluted with phosphate buffer (pH 7.4) to an optical density of 0.70 ± 0.02 at 734 nm. A sample of the hydrogel under study (~10 mg) was added to 2 cm^3^ of the diluted ABTS solution, incubated at 30 °C for 30 min, and the optical density was measured at 734 nm. The degree of radical neutralization was calculated using the formula:

(4) $$\mathrm{AOA}=\frac{A_{0}-A}{A_{0}}\times 100\%$$

where AOA is the antioxidant activity, %; A_0_ is the optical density of the control solution; A is the optical density after contact with the sample.

Unmodified chitosan hydrogel was used as a control.

The content of phenolic groups in the hydrogel was determined using spectrophotometry with the Folin–Denis reagent, following a modified procedure in accordance with the work [29]. Ferulic acid solutions at concentrations ranging from 0 to 100 mg/mL were used to form the calibration curve. A sample of dried hydrogel (10 mg) was placed in a test tube, 1 mL of distilled water, 0.5 mL of Folin–Denis’s reagent (diluted 2 times) and 2 mL of a 20% sodium carbonate solution were added. The mixture was incubated at room temperature in the dark for 30 min, after which the optical density was measured at 760 nm against a blank sample (without hydrogel). The content of phenolic groups was expressed in mg of FA equivalents per 1 g of air-dried gel. All measurements were performed in three repetitions.

The iron-reducing ability of the hydrogel was assessed by the reduction of Fe(III) to Fe(II) in the presence of α,α′-bipyridyl, according to the method described in [30], with modifications. A total of 1 mL of a solution containing 1.25 mM of FeCl_3_·6H_2_O and 3.75 mM of α,α′-bipyridine in ethanol (with a final ethanol concentration of 10%) was mixed with 1 mL of hydrogel solution (1 mg/mL in acetate buffer with pH 5.0). The resulting mixture was incubated at room temperature for 60 min. After that, the optical density of the solution was measured at 512 nm, using a blank sample without hydrogel as a reference. Calibration was carried out using FA solutions with concentrations ranging from 0 to 100 μg/mL. The results were reported in mg of FA equivalents per 1 g of air-dried gel. All measurements were performed in three repetitions.

### 2.5. Quantum Chemical Modeling

Quantum chemical modeling was performed using the GFN2-xTB method [31] in the xTB v program.6.7.1 [32] with the opt extreme flag for geometric optimization with the strictest convergence criteria (Econv ≤ 5 × 10^−8^ Eh, Gconv ≤ 5 × 10^−5^ Eh/Å). Solvent effects were modeled using the continuous Poisson–Boltzmann model [33]. When studying the mechanism of chitosan modification by ferulic acid, the structure of the intermolecular complex of the ferulic acid radical and glucosamine was isolated at the first stage using molecular docking (an algorithm for automated screening of intermolecular interaction centers) [34]. Then, the transition states were searched using the method of metadynamics [35].

The Marcus equation was used to calculate the electron transfer rate constant:

(5) $$k_{\mathrm{ET}}=\frac{2\pi }{\hbar }\frac{{\left|H_{\mathrm{AB}}\right|}^{2}}{\sqrt{4\pi \lambda k_{B}T}}e^{-\;\frac{(-∆G+\lambda {)}^{2}}{4\lambda k_{B}T}}$$

where π is the mathematical constant, 3.141; ℏ is the reduced Planck constant, 1.054571800 × 10^−34^ J × s; HAB is the matrix coupling element; λ is the reorganization energy, J/mol; kB is the Boltzmann constant, 1.380649 × 10^−23^ J/K; ΔG is the Gibbs energy, J/mol T is the temperature, K.

The matrix element of the bond was calculated using the DIPRO method after optimizing the structure of the pre-reaction complex by molecular docking [36]. The Cartesian coordinates of all optimized structures are presented in Supplementary Materials (S2).

## 3. Results and Discussion

### 3.1. Dependence of Chitosan Cross-Linking Degree by Ferulic Acid on the Medium pH

Protonation of amino groups is known to suppress their nucleophilic activity, therefore, in an acidic environment, the number of reactive chitosan centers available for cross-linking decreases. However, in an alkaline environment, the solubility of chitosan deteriorates [37]. In addition, the activity of laccase, which generates phenoxyl radicals from ferulic acid under the action of atmospheric oxygen, also depends on the medium pH [24]. Thus, the optimal pH value of the medium is necessary to be determined for the reaction of chitosan cross-linking with ferulic acid under the action of ScaSL laccase.

**Figure 1:**
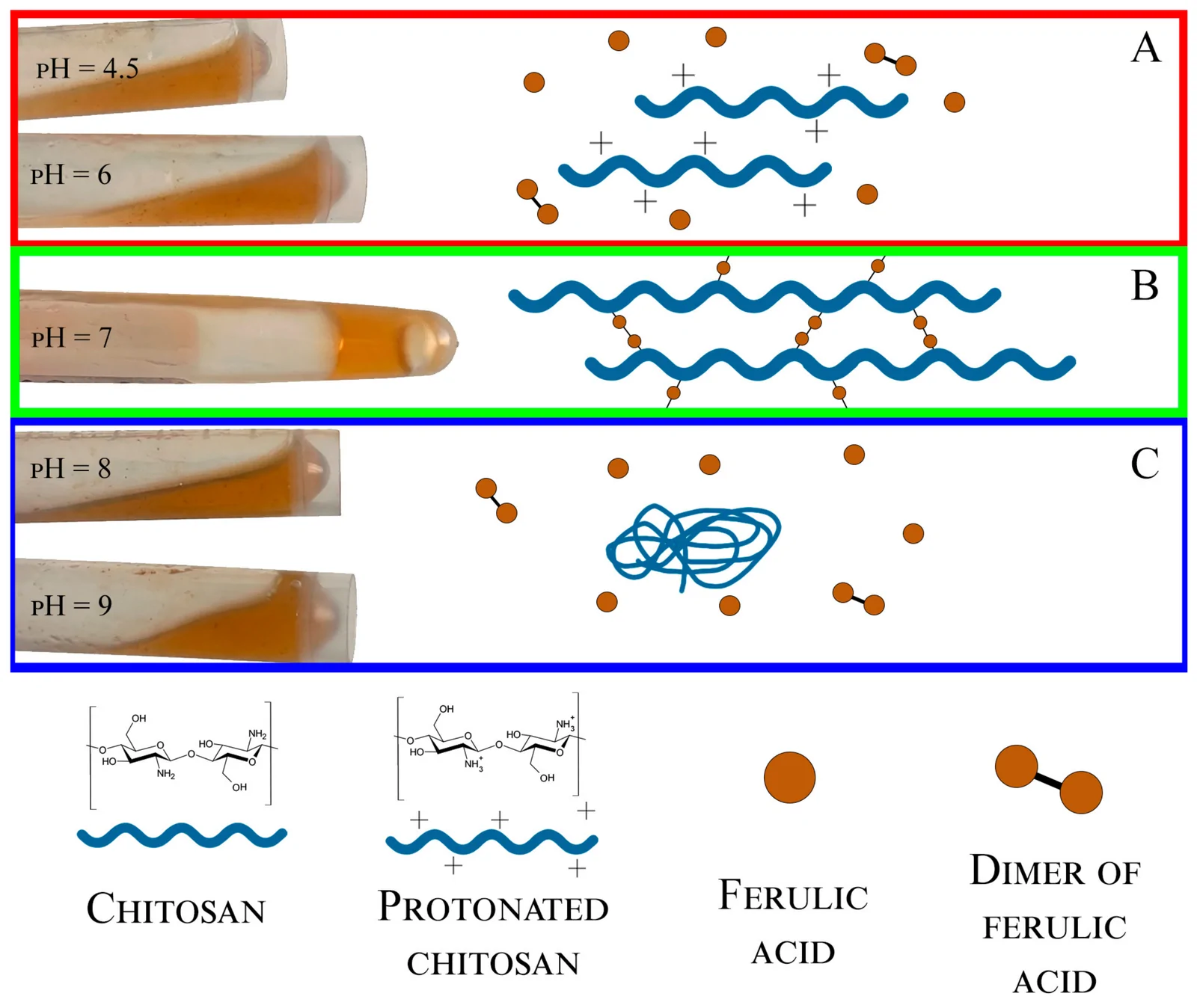
Chitosan gel samples after FA modification in the presence of ScaSL laccase (130 U/g of chitosan) after 72 h at a ratio of n(FA)/n(NH_2_) = 1:10 and various pH values of the reaction mixture: (**A**) slightly acidic medium; (**B**) neutral medium; (**C**) alkaline medium.

The formation of a form-stable hydrogel was observed at pH 7 (Figure 1). In the slightly acidic area (pH 4.5 and 6.0), the viscosity of the reaction mixture did not change significantly, due to a simultaneous increase in the degree of protonation of amino groups [38,39] and a decrease in the catalytic activity of the ScaSL enzyme with respect to phenols in an acidic environment [15]. In an alkaline medium (pH 8.0 and 9.0), turbidity of the gel was observed due to its coagulation.

Thus, cross-linking of chitosan with ferulic acid under the action of bacterial laccase ScaSL should be carried out at a neutral pH value.

### 3.2. Dependence of Chitosan Cross-Linking Degree by Ferulic Acid on the Ratio of Reagents

**Figure 2:**
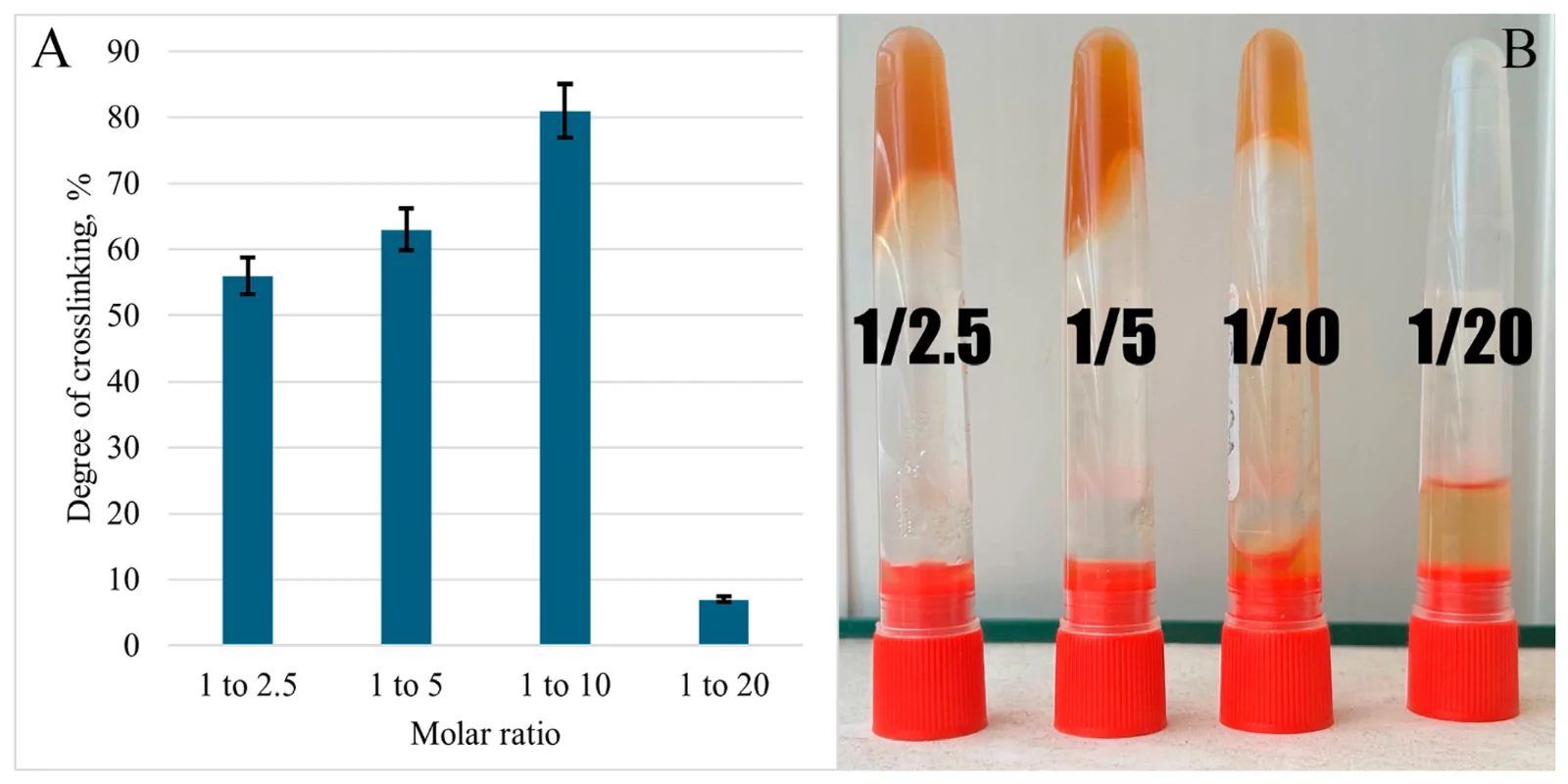
Effect of the molar ratio of ferulic acid/chitosan amino groups (n(FA)/n(NH_2_)) on the degree of cross-linking and consistency of hydrogels: (**A**) The degree of chitosan gels cross-linking depending on the FA ratio and chitosan amino groups (n(FA)/n(NH_2_). (**B**) Photo of hydrogels illustrating the change in their consistency from liquid to form-stable depending on the content of the cross-linking agent.

The ratio of components in the reaction mixture determines the degree of saturation of the polymer matrix with covalent cross-linking and the nature of the three-dimensional mesh formed, ranging from liquid and fluid gels to dense, strain-resistant materials with high cross-bond density. The shape stability of the obtained gels was visually assessed, and the degree of cross-linking was calculated by changing the mass of the samples soaked in a 3% acetic acid solution (Figure 2).

Biomaterial samples obtained with an intermediate ratio of n(FA)/n(NH_2_) 1:10 showed the best shape stability (Figure 2). Gel films had formed after 72 h of stirring in air and distribution of the reaction mixture over the substrate. Their thickness was 0.5 mm after drying. An increase in the proportion of FA also led to the production of form-stable gels, but their coloring was more intense due to the formation of dark-colored products of oxidative polymerization of phenylpropanoid residues (Figure 2B). Excessive cross-linking can reduce the diffusion permeability of the gel, since an increase in the degree of cross-linking leads to the formation of a denser mesh, which limits the release of incorporated substances [40,41]. With a decrease in the FA content, the gels remained fluid due to the insufficient number of cross-linkages formed (Figure 2B). Table 1 shows the conditions for production and characteristics of cross-linked chitosan gels obtained in other studies.

In this study, the comparable enzyme activity has made it possible to achieve the highest degree of cross-linking, which is explained by the unique properties of laccase ScaSL. Unlike fungal laccases, ScaSL exhibits pronounced catalytic activity against phenolic substrates precisely at neutral pH values; therefore it has advantages over analogues in reactions of nucleophilic modification of chitosan by phenylpropanoids.

### 3.3. The Mechanism of Chitosan Cross-Linking by Ferulic Acid Under the Action of Laccase

**Figure 3:**
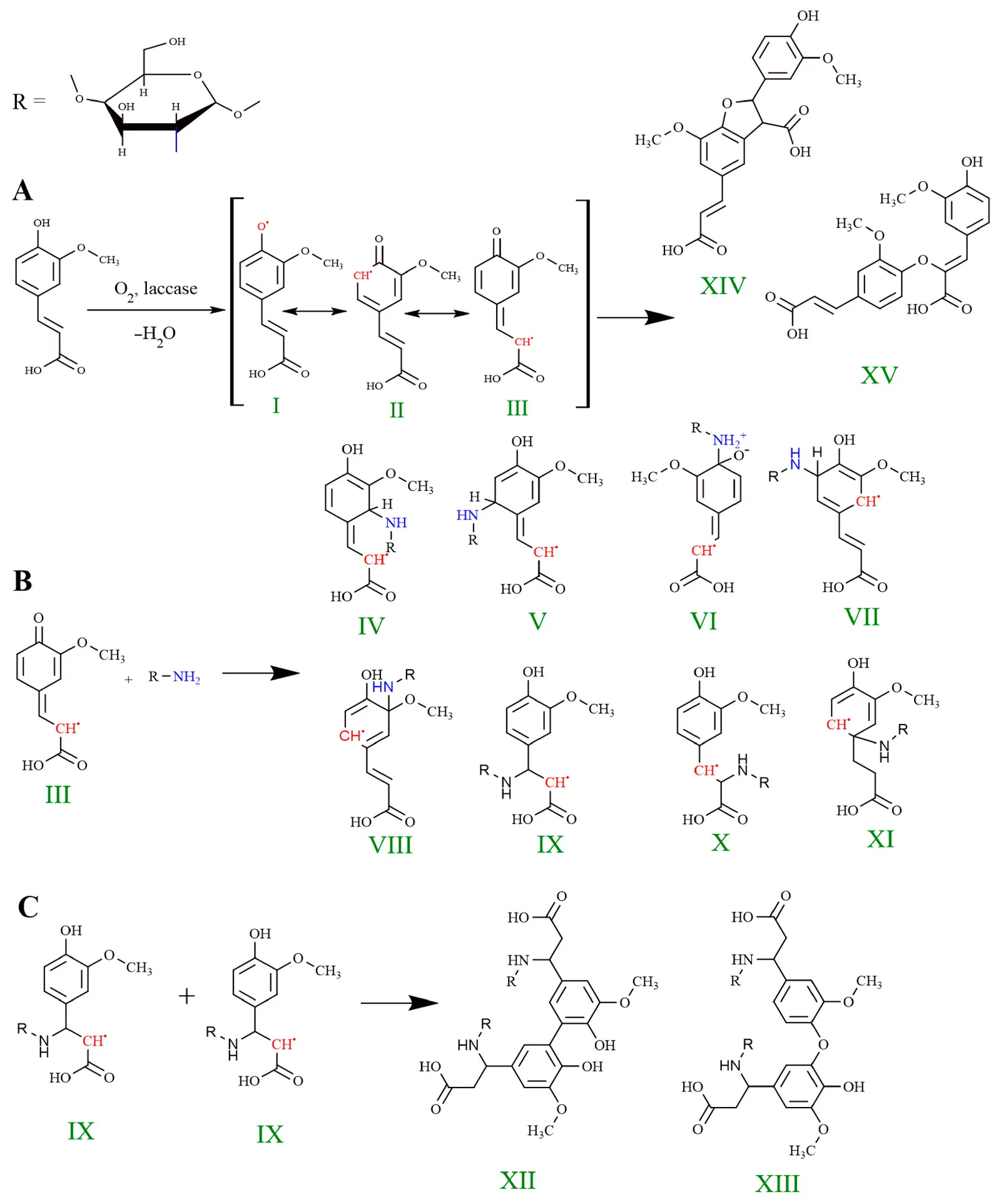
Possible processes occurring during the interaction of glucosamine (a model fragment of chitosan) with ferulic acid under the action of laccase: (**A**) oxidation of ferulic acid to the corresponding phenoxyl radical followed by dimerization; (**B**) possible ways of interaction of the amino group of glucosamine with the ferulic acid radical; (**C**) covalent cross-linking of two glucosamine molecules through ferulic acid radicals. The structures of possible intermediate compounds are indicated by roman numbers.

Despite the active use of laccase for chitosan cross-linking by ferulic acid, the detailed mechanism of cross-linking remains questionable [14,19,20,21,42]. Chitosan amino groups are traditionally believed to interact with oxidized forms of ferulic acid by joining Michael or forming Schiff bases. However, HPLC-MS data indicate that the end-products of FA laccase oxidation are dimers devoid of quinoid fragments and, consequently, incapable of covalent binding to amines [11,12,13]. In furtherance of the hypothesis proposed in [25] about the direct interaction of chitosan amino groups with the phenoxyl radical, we previously considered the latter as a carbonyl compound (resonance structure III, Figure 3A) capable of entering Michael reactions (according to C2/C6) and Schiff reactions (according to C4) [26]. Quantum chemical modeling using the PM6-DH2 method has shown that the main reaction pathway is the addition of a Michael in a pair position to the methoxy group (C6) [26], which is consistent with the conclusions of [25]. In this study, we improve the accuracy of calculations by applying a more modern method of quantum chemical modeling GFN2-xTB, and for the first time we conduct a systematic analysis of the reactivity of all carbon atoms of the skeleton of the FA radical, considering their potential equivalence within an extended conjugate system.

At the first stage, the intermolecular interaction between the ferulic acid radical and glucosamine as a model fragment of chitosan (Supplementary Materials S2, Figure S1) was considered using the molecular docking method. In view of the complex structure, stabilized by a hydrogen bond, amino group–carboxyl, it is possible to assume the attack of the amino group specifically on the phenylpropanoid fragment. To quantify this hypothesis, activation barriers (∆G) were calculated for all possible attachment positions of the chitosan amino group to the FA radical (Figure 3B, Table 2).

According to Hammond’s postulate, the preferred direction of the reaction is determined by the stability of the corresponding transition states [27]. Based on the energy of the formation of transition states, it can be assumed which of the products will be the main one. The lowest energy barrier (ΔG^‡^ = 8.75 kJ/mol) is observed when the amino group of glucosamine is attached to the beta position of the phenylpropanoid fragment relative to the carboxyl group (structure IX). The preference for this position is explained by the lower stability of the side chain compared to the aromatic ring and the strong polarization of the double bond under the action of two electron acceptor groups—the carboxylic and benzene rings with a phenoxyl radical. The significant positive charge that occurs on the β-carbon atom makes it an ideal center for a nucleophilic attack.

**Figure 4:**
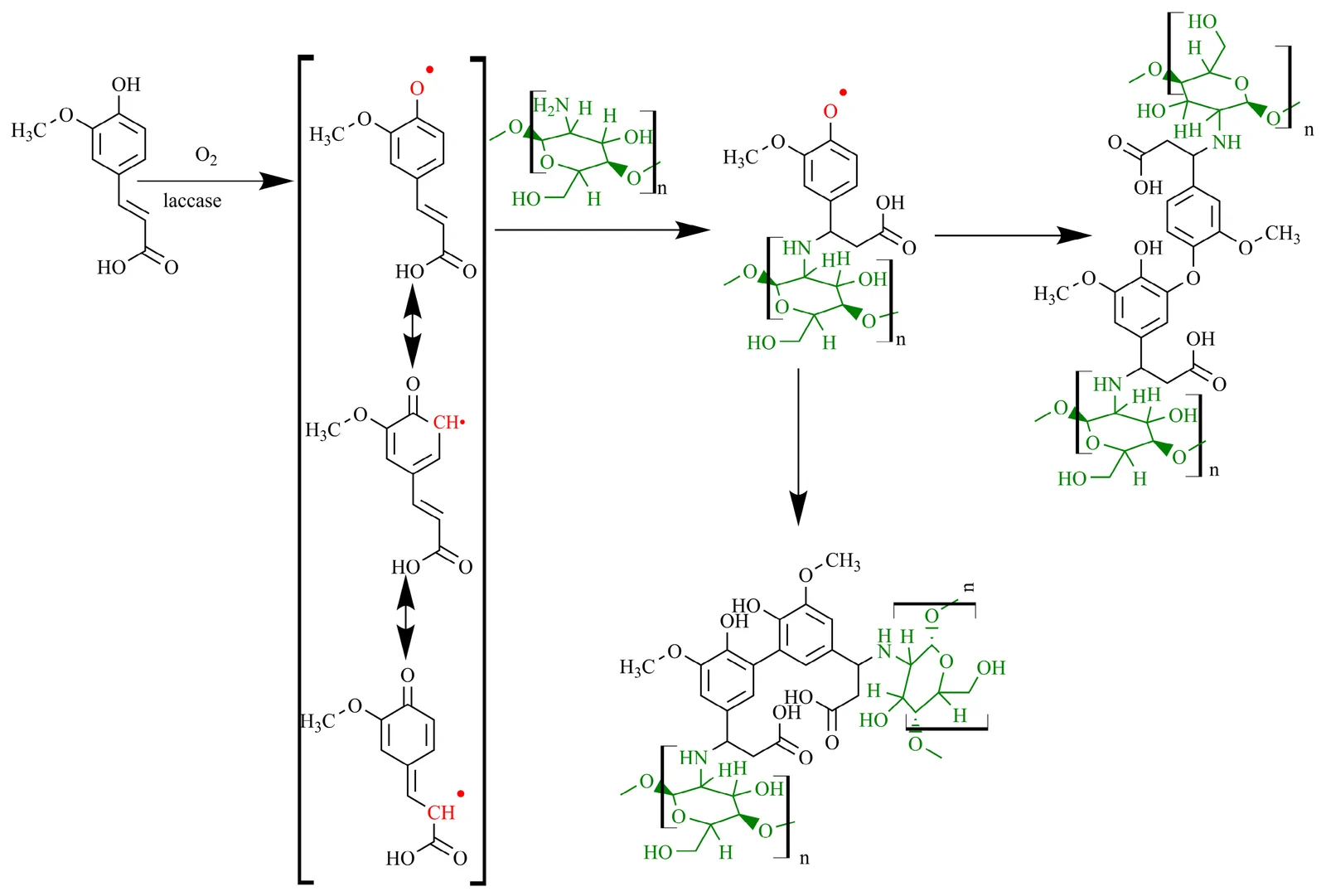
Proposed mechanism of FA chitosan cross-linking under the action of laccase. Chitosan fragments are indicated in green, radicals are indicated in red.

Phenoxyl radicals covalently bound to glucosamine amino groups subsequently recombine with each other to form cross-links, with the formation of two different structures possible (Figure 4). To select the predominantly formed reaction product, the corresponding energy barriers were calculated (Table 2). The formation of both products is characterized by negative Gibbs activation energy values (ΔG^‡^XIII = −63.52; ΔG^‡^XIII = −148.17 kJ/mol). This activity is typical for radical recombination, which often proceeds as a barrier-free diffusion-controlled process [44]. Consequently, the distribution of products is determined not by the energy barrier, but by the probability of meeting radicals in the solution, which leads to the equally probable formation of both compounds. The generalized mechanism of FA chitosan cross-linking under the action of laccase is illustrated in Figure 4.

**Figure 5:**
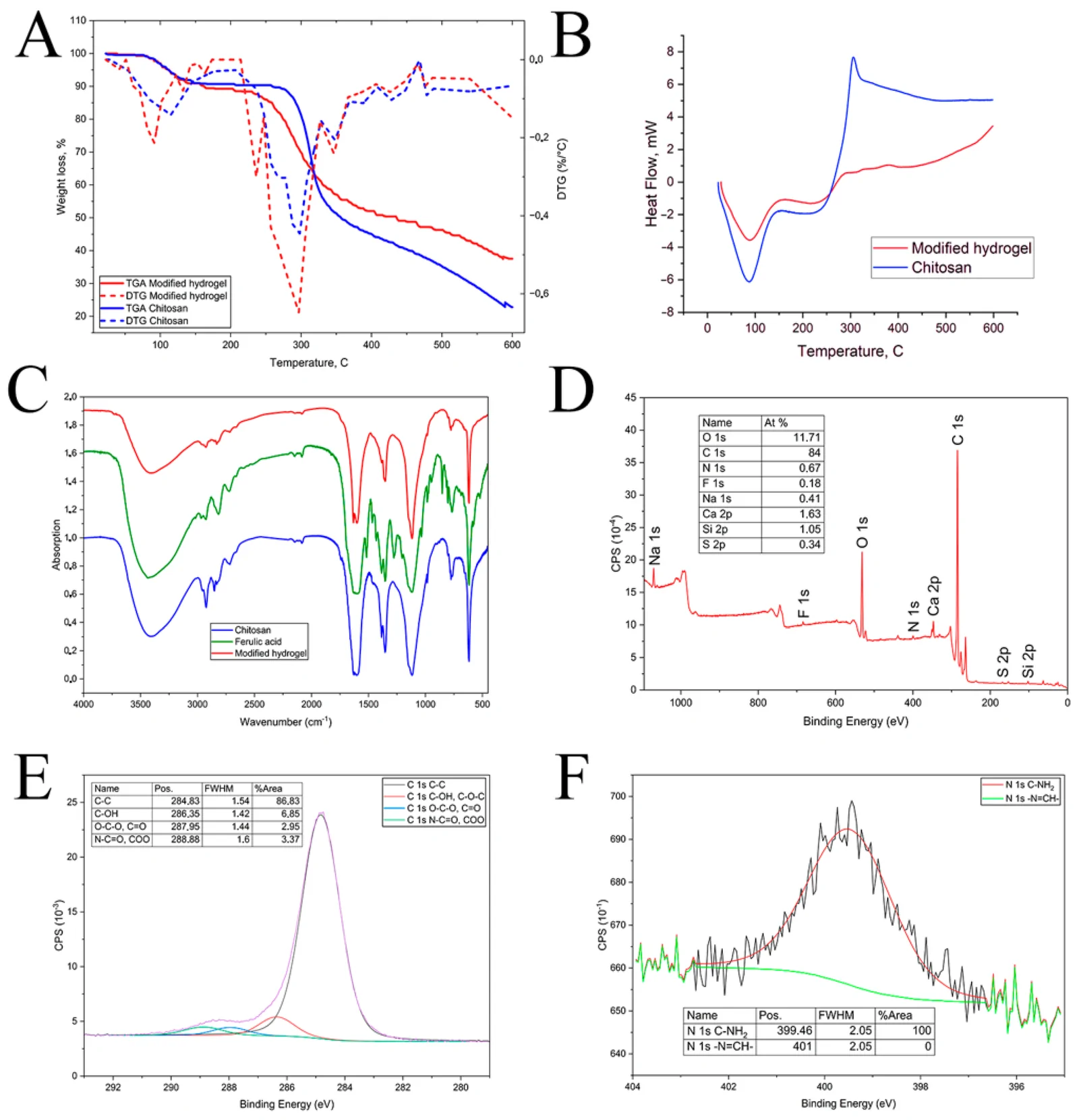
Characterization of the chitosan structure modified with ferulic acid in the presence of ScaSL laccase (130 U/g chitosan) at pH 7.0 with a ratio of n(FA)/n(NH_2_) = 1:10: (**A**) Thermogravimetric analysis (TGA) and differential curve (DTG); (**B**) differential scanning calorimetry curves; (**C**) FTIR spectra; (**D**) overview XPS spectrum of modified chitosan; (**E**) XPS–high-resolution spectrum of the C 1s band; (**F**) XPS-high-resolution spectrum of the N 1s band (the original sample and the results of its mathematical deconvolution). The experimental spectrum is shown as a solid black line. The colored lines indicate the individual components obtained after deconvolution: green—C–N bonds characteristic of amino groups (–NH_2_); red—C=N bonds characteristic of azomethine groups (–CH=N–).

The structure of a chitosan gel cross-linked with FA was characterized by synchronous thermal analysis, FTIR, and XPS spectroscopy to confirm the proposed mechanism experimentally (Figure 5).

Thermograms of the initial and modified chitosan demonstrate two main stages of mass loss (Figure 5A). The initial stage, which is already fixed at 50 °C, corresponds to the removal of physically sorbed water, which is confirmed by a clear endothermic peak on the DSC curve (Figure 5B). For a more detailed analysis of the temperature ranges of degradation, DTG curves are also presented in Figure 5A. In the DTG curve of the initial hydrogel (blue line), two main peaks of mass loss can be clearly observed. The first low-temperature peak in the range of 80–120 °C, is associated with the removal of bound and free water. The second high-temperature peak in the range of 250–300 °C, corresponds to the destruction of polysaccharide chains.

For the modified sample (red line), the DTG pattern is more complex and multimodal; there is an additional shoulder or peak between 180 and 220 °C. We attribute this change to the introduction of polyphenolic components, which contributes to an additional stage of thermal oxidative degradation. Therefore, DTG data confirm the two-stage nature of decomposition in the initial sample.

The main primary product of thermal decarboxylation of ferulic acid in the condensed phase is 4-vinyl glycol, which is formed at temperatures above 170 °C [45,46]. The mass loss of modified chitosan in the range of 220–260 °C is consistent with the formation and evaporation of 4-vinyl glycol (boiling point 224 °C). The second, main stage of weight loss proceeds up to 350 °C and is accompanied by significant destruction of the polymer matrix. For the initial chitosan, an acute exothermic peak is observed at this stage, due to the rupture of glycoside bonds and subsequent cascading degradation reactions of pyranose cycles [47,48]. In contrast, the chitosan–ferulic acid film demonstrates a smoother increase in heat flow and a lower rate of mass loss in this temperature range. The DSC curve of chitin, the fully acylated form of chitosan, is known to lack a sharp exothermic maximum in the region of ~350 °C, whereas the intensity of this peak grows with increasing degree of deacetylation [48]. Consequently, the disappearance of the characteristic exothermic peak on the thermogram of chitosan modified with ferulic acid occurs due to a decrease in the proportion of free amino groups. A gradual loss of mass above 350 °C may be associated with the gradual rupture of the cross-linked chitosan–ferulic acid networks, which triggers the simultaneous decomposition of both the acid and the polymer. Thus, the TG-DSC data indicate the presence of both free and covalently bound ferulic acid residues in the modified chitosan and also confirm a change in the thermal stability of the polymer due to its chemical modification. The FTIR spectra of ferulic acid, the initial and modified chitosan, were analyzed to determine the basic chemical bonds in modified chitosan (Figure 5C). The spectrum of modified chitosan lacks an absorption band at 1635 cm^−1^ corresponding to fluctuations in the C=N bond, which should be present in the case of Schiff base formation [49]. The absence of this signal provides direct experimental evidence that excludes the formation of azomethine bonds and, consequently, the mechanism of cross-linking through the Schiff base. Comparison of the spectrum of the initial FA with the spectrum of the modified chitosan revealed the disappearance of a few absorption bands in the range of 1400–1600 cm^−1^ corresponding to valence vibrations of C=C bonds in the FA aromatic ring. This is due to the appearance of additional substituents in the aromatic cycle because of FA dimerization. The appearance of FA dimers is also confirmed by the disappearance after cross-linking of a series of bands in the areas of 800–900 cm^−1^ corresponding to valence vibrations of the C–H bonds of the aromatic ring. Thus, the FTIR spectrum of the cross-linked gel supports the proposed mechanism of covalent cross-linking of chitosan amino groups with the phenylpropanoid skeleton of ferulic acid.

Additionally, the cross-linked chitosan was characterized by X-ray photoelectron spectroscopy (Figure 5D). The discrepancy between the experimentally measured (C 84.0 atm.%; N 0.67 at.%; O 11.7 at.%) and theoretically calculated (C 56.5 at.%; N 8.1 at.%; O 35.5 atm.%) elemental composition indicates differences composition on the surface and in the volume of the material. The surface of the studied sample is enriched in carbon and depleted in nitrogen on a scale that cannot be explained by simple adsorption of atmospheric pollutants, since typical values for unmodified chitosan according to XPS data are C ~54–67 at.%, N ~7–12 at.% [50]. The adsorption of ferulic acid oligomers on the surface of the cross-linked chitosan can explain the observed effect. Enzymatic oxidation of ferulic acid generates highly reactive phenoxyl radicals that enter two competing processes.: covalent binding to chitosan amino groups and recombination with each other to form dimers and oligomers. The latter process leads to the formation of polymer sections enriched with aromatic fragments on the surface due to the higher concentration of ferulic acid at the surface of the chitosan hydrogel, rather than in its volume. The XPS method, with an analysis depth of only 5–10 nm [51], mainly registers this surface layer, which explains the extremely low nitrogen content and high carbon content in the spectrum (Figure 5D).

Analysis of the high-resolution XPS spectra of C 1s and N 1s allowed us to confirm the proposed mechanism of nucleophilic attachment of chitosan amino groups to the phenylpropanoid skeleton of ferulic acid. Four characteristic signals are registered in the C 1s spectrum. The most intense peak at 286.35 eV corresponds to carbon atoms bound to hydroxyl groups (C–OH) and carbon atoms of the glycoside bond (C–O–C) of the chitosan backbone [52]. The signal at 284.83 eV refers to the hydrocarbon skeleton of the polymer (C–C/C–H) and fragments of ferulic acid [53]. The component at 287.95 eV corresponds to carbonyl groups (C=O) and acetal fragments (O–C–O) of chitosan [52]. The peak at 288.88 eV is in the region characteristic of chitosan amide groups (N–C=O) and ferulic acid carboxyl groups (O–C=O) [54].

The high-resolution XPS spectrum of N 1s shows a single symmetrical signal at 399.46 eV, corresponding to free amino groups (–NH_2_) and secondary amines (–NH–) formed during nucleophilic addition. This value of the binding energy agrees well with the literature data for the N–C bond in chitosan (399.4 eV) [52,54] and secondary amines (399.5 eV) [53]. The N 1s spectrum lacks a signal in the range of 401.0–402.0 eV, associated with azomethine groups (–N=C). This unambiguously excludes the implementation of the mechanism occurring with the formation of the Schiff base [50]. Thus, the totality of high-resolution XPS data confirms the successful covalent attachment of ferulic acid to chitosan and confirms the mechanism proposed above, which eliminates the formation of Schiff bases.

Thus, the mechanism of laccase-catalyzed cross-linking of chitosan with ferulic acid has been proposed based on experimental and theoretical studies. The covalent nature of the modification has been confirmed by data from thermogravimetric and differential scanning calorimetric analysis. The absence of the Schiff base in the cross-linked biopolymer has been experimentally confirmed using FTIR and XPS spectroscopy techniques. The preferred position of amino group attachment to the phenolic radical has been theoretically justified using GFN2-xTB quantum chemical modeling.

### 3.4. Characteristics of Modified Chitosan Films Functionality

The practical value of materials based on cross-linked chitosan is determined not only by their structural features, but also by their operational characteristics, primarily morphology, water swelling, oxygen permeability, resistance in various media, antioxidant, and antimicrobial activity. A comprehensive study of these properties was carried out for samples obtained under optimal conditions (pH 7.0, ratio n(FA)/n(NH_2_) = 1:10).

### 3.5. Morphology, Water Swelling, and Oxygen Permeability of the Film

The scanning electron microscopy (SEM) method is the main tool for visualizing the microstructure of the surface of chitosan-based gels, since it allows direct observation of morphological changes caused by cross-linking or functionalization (Figure S2). A chitosan film modified with ferulic acid in the presence of ScaSL laccase (2.6 U) at pH 7.0 and a ratio of n(FA)/n(NH_2_) = 1:10 has a uniform smooth surface, which is typical for cross-linked gels of this polysaccharide [55]. Micrographs show only small inclusions, probably representing ferulic acid oligomers. This morphology indicates a high compatibility of the components and the absence of microphase separation, which is an important prerequisite for achieving uniform physico-chemical characteristics throughout the entire volume of the material. The homogeneous morphology of hydrogels provides favorable conditions for oxygen diffusion [56].

The absence of pronounced structural defects and dense agglomerates favors the free diffusion of gases. Indeed, the modified chitosan film has demonstrated high permeability to oxygen. When applying a 0.5 mm thick film to the surface of the Clark electrode, the measured dissolved oxygen content without air bubbling was 1.2 ± 0.2 mg O_2_/L compared to 1.6 ± 0.3 mg O_2_/L in the absence of a film and with bubbling 9 ± 1 mg O_2_/L compared to 9.6 ± 0.5 mg O_2_/L. This confirms that the three-dimensional hydrogel network formed by laccase-catalyzed chitosan cross-linking with ferulic acid retains excellent gas transport properties necessary to ensure gas exchange in damaged tissues, stimulate angiogenesis, and prevent the development of anaerobic infection [40]. The technique with the Clark electrode, which is used in this study, is a validated approach for assessing the oxygen transport characteristics of materials in contact with tissue, and it provides reproducible results [28]. Chitosan-based hydrogels have a high level of oxygen permeability, which makes them an important treatment option for wounds and burns [57,58]. Oxygen diffusion through a hydrogel film based on modified chitosan slightly decreases, providing an oxygen concentration after diffusion of approximately 9.0 mg O_2_/L at 18 °C. This confirms the high oxygen permeability of the material and its ability to maintain aerobic conditions in the wound.

The swelling degree of the modified gel reaches 270%, which is comparable to the swelling of the modified chitosan gel obtained in another study [59]. A high swelling degree is critically important for the use of the material as a dressing, since it ensures the absorption of wound exudate and the maintenance of a moist environment [60] and creates prerequisites for the controlled release of medicinal substances. The proximity of the swelling values to well-known cross-linked systems additionally indicates that enzymatic grafting of phenylpropanoids does not lead to excessive compaction of the hydrogel network.

Thus, the resulting chitosan-based gel cross-linked with ferulic acid is characterized by a uniform smooth microstructure without signs of phase separation, which provides a combination of high oxygen permeability and a significant degree of swelling (270%). The combination of these interrelated properties indicates the promise of the developed material as a biocompatible wound coating capable of providing efficient gas exchange, absorbing exudate and maintaining an optimal moist environment for healing.

### 3.6. Stability in Aquatic and Organic Environments

The resistance of modified chitosan to aggressive media is a critical parameter that determines its functionality in real life conditions, such as in contact with wound exudate, medications, or disinfectants. To assess the stability of chitosan films cross-linked with ferulic acid, the samples were incubated in media simulating a wide range of chemical effects: Britton–Robinson buffer solutions in the pH range 4.5–12, 0.3% hydrogen peroxide solution as a strong oxidizer, and 95% ethanol as a polar organic solvent.

Treatment with 95% ethanol caused a moderate weight loss (15%), which is probably due to the extraction of bound water and low molecular weight oxidation products of FA, rather than degradation of the cross-linked polymer matrix. Hydrogen peroxide showed the greatest destructive activity, which initiated the loss of (71 ± 8)% of the sample mass. Exposure to H_2_O_2_ was accompanied by a macroscopic violation of the integrity of the film. The material sample turned into a jelly-like state, and the characteristic coloration caused by fragments of ferulic acid oligomers significantly weakened. This effect accords well with the known mechanism of oxidative depolymerization of chitosan, which proceeds through the breaking of glycosidic bonds under the action of hydroxyl radicals generated during the decomposition of peroxide [61].

**Figure 6:**
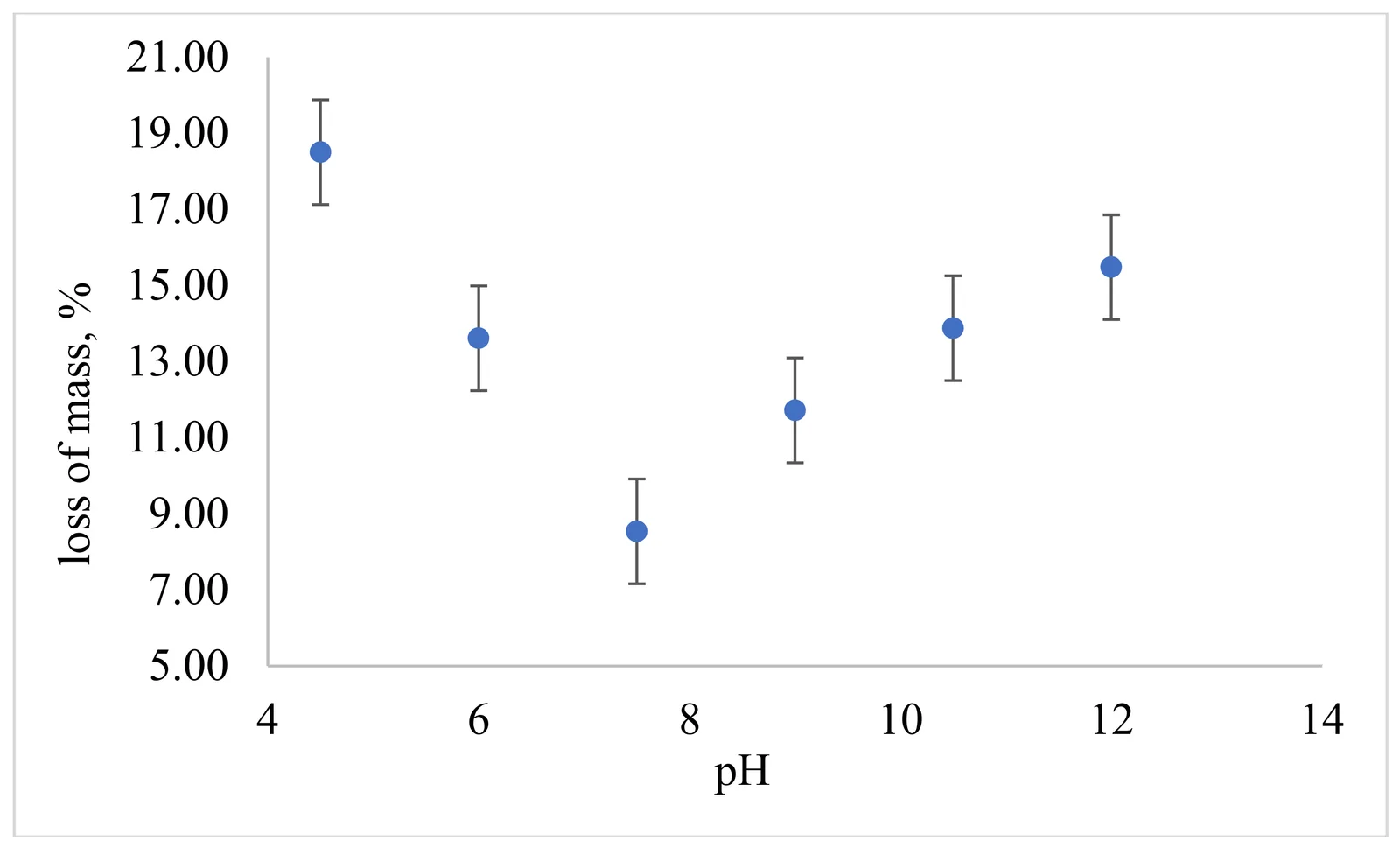
pH medium effect on the mass loss of chitosan films modified with ferulic acid under conditions of laccase catalysis (ScaSL, 130 U/g, pH 7.0, n(FA)/n(NH_2_) = 1:10). The data are presented with standard deviations (*p* 0.95, n = 3).

The mass loss profile was non-monotonic in media with different acidity (Figure 6).

The maximum mass loss of samples in an acidic environment (19% at pH 4.5) is caused both by protonation of free chitosan amino groups and the transition of polymer chains not embedded in a three-dimensional grid into solution. The lowest mass loss has been recorded in the neutral region (9% at pH 7.5), where chitosan is in a deprotonated state, and ferulic acid, which is not bound to the polymer, is practically insoluble. A further shift in pH to the alkaline region leads to a gradual increase in mass loss (up to 15% at pH 12), which is explained by ionization and subsequent leaching of residual ferulic acid and its condensation products not bound to chitosan covalently. It should be noted that the initial mass content of ferulic acid is about 50%, which is quite sufficient for the observed effect.

Thus, the modified chitosan–ferulic acid films demonstrate high stability in neutral and slightly alkaline environments, while maintaining their structural integrity; they undergo the expected degradation in a highly oxidizing environment. The data obtained confirm that weight loss in acidic and alcoholic media is controlled primarily by physical leaching of unreacted components, rather than by the destruction of the cross-linked mesh, which indicates the stability of covalent bonds formed during the laccase-catalyzed modification of chitosan with ferulic acid.

### 3.7. Antioxidant Activity

The antioxidant activity of modified chitosan gels was evaluated spectrophotometrically by the reduction degree of the radical cation ABTS^+•^ [42,62]. Incubation of unmodified chitosan hydrogel with a solution of the radical cation ABTS^+•^ resulted in a decrease in optical density by 25%, which is consistent with the results presented in other chitosan studies [42,62]. NH_2_ groups of amino sugars has shown to provide antiradical activity [63]. An alternative mechanism of chitosan antioxidant activity is reversible radical cation sorption.

Chitosan modified with ferulic acid exhibits high antioxidant activity (Table 3). 

This is due to the significant content of ferulic acid oligomers covalently immobilized on the polymer chain, the phenolic nature of which ensures effective neutralization of ABTS^+•^ cation radicals.

The pronounced antioxidant effect of hydrogels obtained using *Agaricus bisporus* laccase is achieved by using non-individual ferulic acid as a cross-linking agent, but an enzymatic lignin hydrolysate containing FA and other phenolic compounds with antioxidant activity [42]. It should be noted that the two-domain laccase ScaSL makes it possible to obtain cross-linked FA chitosan with maximum antioxidant activity among similar modified chitosan gels (Table 4).

To comprehensively assess the antioxidant properties of the hydrogels obtained, in addition to the ABTS test, the Folin–Denis method was used to quantify phenolic hydroxyls [29] and the method with a bipyridyl iron complex to evaluate iron-reducing ability [30]. The content of phenolic groups determined by the Folin–Denis method was 33 mg FC per 1 g of dried gel, which is lower than the theoretical content (91 mg FC/g). This is due to the high degree of hydrogel cross-linking (81%), as a result of which some phenolic groups are inaccessible for direct determination. The observed results are typical for cross-linked chitosan materials. The iron-reducing ability, measured with a bipyridyl iron complex at pH 5.0, was 7.7 mg of FC/g of gel. This is lower than the value obtained with the Folin–Denis method. This discrepancy may be explained by the fact that, at an acidic pH, ferulic acid’s phenolic hydroxyls are predominantly protonated, decreasing their ability to reduce iron ions [64].

The combined use of three different methods (ABTS, Folin–Denis, and the bipyridyl iron complex) makes it possible to obtain various but complementary information about the antioxidant activity of the material under study, which corresponds to modern approaches to evaluating such polymer systems [65,66]. The Folin–Denis method in an alkaline medium (pH ~10) determines the reducing ability of phenolic compounds by reducing phosphoric–molybdenum–tungsten heteropolyacid with a phenolate ion [65,66], whereas the method with a bipyridyl iron complex at pH 5.0 evaluates the reducing ability of these compounds in an acidic medium [64]. It should be noted that the high reducing ability of a material in methods involving a bipyridyl iron complex does not always indicate low antioxidant activity. Ascorbic acid (vitamin C), for example, is a classic case. In physiological concentrations, it functions as an antioxidant. However, in the presence of free ions of transition metals such as iron or copper, it can exhibit pro-oxidant properties [67]. The mechanism of this effect is the ability of ascorbate to reduce Fe^3+^ to Fe^2+^ or Cu^2+^ to Cu^+^, which then catalyze the Fenton reaction to produce highly toxic hydroxyl radicals. In this regard, the moderate iron-reducing ability of the developed hydrogel (7.7 mg FC/g) is a favorable factor for biomedical application, since it reduces the risk of undesirable prooxidant effects caused by transition metal ions. Thus, a comprehensive assessment of antioxidant activity shows that the developed hydrogel combines high antiradical activity (95%, according to ABTS) with a low prooxidant potential. This makes it a promising material for use as a wound coating or other biocompatible materials.

The electron transfer parameters were calculated in accordance with the classical Marcus theory to substantiate theoretically the high antioxidant activity of chitosan cross-linked with ferulic acid under the action of laccase [68]. NO_2_^•^ has been chosen as a model oxidant because it satisfies a set of requirements for a reference electron acceptor in the study of the antioxidant activity of phenylpropanoids. Firstly, NO_2_^•^ is an endogenous radical formed in biological media during the peroxynitrite decomposition [49] and in enzymatic processes [69], which ensures the biomedical relevance of the model. Secondly, the interaction of NO_2_^•^ with phenolate ions proceeds exclusively by the mechanism of external electron transfer, which is accompanied by neither the formation of adducts nor by the transfer of a hydrogen atom; this guarantees the "purity" of the oxidative act and an unambiguous interpretation of the measured kinetic parameters [70]. Thirdly, the experimental rate constant of the reaction of NO_2_^•^ with ferulate ion (lgk_ET_ = 8.87) is an order of magnitude lower than the diffusion limit (lgk_diff_ = 9.85), due to which the process is in the kinetic region. Thus, NO_2_^•^ exhibits high sensitivity to changes in the electronic structure of the substrate, which makes this radical ideal for validating quantum chemical models of electron transfer and establishing structure-reactivity correlations. The ability of modified chitosan to intercept NO_2_^•^ radicals was compared with the activity of the initial phenylpropanoid, ferulic acid, as well as its dimeric products, which could be formed in laccase-catalyzed oxidative condensation (Table 4, Figure 3A).

The calculated standard Gibbs energy of single-electron oxidation of the ferulate ion to the corresponding phenoxyl radical (−60.74 kJ/mol) agrees well with the experimental value determined by pulsed radiolysis (−62.0 kJ/mol). The electron transfer rate is also reproduced with high accuracy (the lgk_ET_ error does not exceed 3.3% relative to the experimental value lgk_ET_ = 8.87 from [71]). This indicates that the theoretical approach we used, the GFN2–xTB method in combination with the continuous Poisson–Boltzmann model, describes the studied systems well.

The Gibbs free energy of single-electron oxidation is more negative for chitosan modified with ferulic acid and for ferulic acid dimers, than for ferulic acid itself. The effect is more pronounced for the chitosan–ferulic acid conjugate (an increase in |ΔG| by 40–80% relative to the monomer) than for ferulic acid dimers (an increase in |ΔG| of the order of 16%). There is a slight increase in the energy of reorganization for all considered structures, which is explained by the large size of the molecules compared with the initial ferulic acid. Nevertheless, despite the thermodynamically more advantageous oxidation, the electron transfer rate constant decreases for both chitosan–ferulic acid conjugate and ferulic acid dimers. This decrease is due to a significant decrease in the matrix element of the HAB bond, the physical meaning of which is the probability of quantum electron tunneling from the donor (ferulic acid and its derivatives) to the acceptor (NO_2_^•^). A decrease in the electron transfer rate, constant during the transition from monomeric to oligomeric forms, is a phenomenon characteristic of large molecules, especially if the enlargement of the conjugate system is accompanied by a delocalization of the electron density. An increase in the length of the coupled system leads to a more uniform distribution of the electron density and, as a result, to a decrease in the probability of quantum electron tunneling. A similar pattern was previously described for aromatic hydrocarbons. The electron transfer rate decreases with an increase in the number of carbon atoms and the size of the π-system [72]. Thus, the totality of the data obtained allows us to draw the following conclusions about the reaction mechanism. Experimentally confirmed are: 1. The covalent nature of ferulic acid binding to chitosan (as evidenced by TG/DSC data); and 2. the absence of Schiff base formation as a possible cross-linking pathway, as indicated by the absence of characteristic signals in FTIR and XPS spectra. Theoretically justified (based on quantum chemical modeling) is the preferred addition of an amino group at the β-position of the propanoid fragment of the phenoxyl radical. Therefore, the proposed mechanism involving nucleophilic addition followed by radical recombination is consistent with the available experimental evidence, while an alternative pathway through Schiff base formation has been excluded based on FTIR and XPS spectroscopic data.

## 4. Conclusions

The study proves that bacterial laccase ScaSL, with an average redox potential of the T1 center (+0.47 V), is a highly effective biocatalyst for enzymatic cross-linking of chitosan with ferulic acid in a neutral medium. The selected conditions (pH 7.0, molar ratio FA/NH_2_ = 1:10) provide a high degree of 81% cross-linking, and the resulting material combines antioxidant (neutralization of 95% ABTS^+•^) with high oxygen permeability and stability. Quantum chemical calculations have confirmed that the reaction follows the path of addition of the chitosan amino group by a double bond of the propanoid fragment of the phenoxyl radical.

Further research is advisable to focus on a comprehensive assessment of the biocompatibility and biodegradation of the obtained hydrogels in in vitro and in vivo model systems, which will determine their suitability for clinical use. A promising area for future research is the optimization of hydrogel production and a thorough evaluation of their mechanical properties, such as tensile strength, elasticity, and rheological characteristics, for potential applications in the biomedical and food industries. An important task is to study the possibility of controlled release of drugs from a cross-linked polymer matrix, which opens prospects for creating targeted drug delivery systems. Scaling up the processes of enzymatic synthesis and the development of chitosan–ferulic acid films based on it, wound coatings, active packaging materials and antioxidant additives for the food and medical industries will contribute to the practical implementation of the results obtained. It is important to emphasize that while the measured physico-chemical characteristics are necessary prerequisites for the effectiveness of hydrogel as a wound dressing, they are not sufficient to prove its efficacy. These properties do justify the potential suitability of the material for creating wound coatings. However, final conclusions about the biomedical applicability of the hydrogel can only be made after conducting comprehensive studies on biocompatibility, cytotoxicity, and regenerative activity using in vitro and in vivo models as part of further research.

## Figures and Tables

**Table 1:** Conditions for obtaining and degree of chitosan gels cross-linking.

The Source of Laccase	Activity	Maximum Degree of Cross-Linking	n(FA)/n(NH_2_)	pH	Ref.
*Myceliophthora thermophila* (fungus)	1 U	21%	1/30	6.0	[19]
*Myceliophthora thermophila* (fungus)	1 U	38.8%	1/2.5	7.5	[20]
*Myceliophthora thermophila* (fungus)	1 U	39.0%	1/2.5	7.5	[21]
*Agaricus bisporus* (mushroom)	1 U	70%	-	4.58	[42]
*Bacillus vallismortis* fmb-103 (bacteria)	3 U	22.5%	1/9	6.5	[43]
*Streptomyces carpinensis*	2.6 U	81%	1/10	7.0	This study

**Table 2:** Parameters of the transition states formed by the interaction of the ferulic acid radical with glucosamine.

The Structures of Possible Intermediate Compounds	Gibbs Activation Energy ΔG^‡^, kJ/mol	Imaginary Frequency ν, cm^−1^
IV	32.97	−3.65
V	79.98	−3.31
VI	25.64	−7.28
VII	35.14	−8.81
VIII	21.30	−8.29
IX	8.75	−7.49
X	44.91	−7.31
XI	18.34	−11.04

**Table 3:** Antioxidant activity of chitosan cross-linked with ferulic acid under the action of laccase.

Sourse of Laccase	Incubation Time	Result	Ref.
*Myceliophthora thermophila*	30 min	51.2%	[21]
*Agaricus bisporus*	40 min	90%	[42]
*Streptomyces carpinensis*	30 min	95%	This study

**Table 4:** Thermodynamic and kinetic parameters of single-electron oxidation of modified chitosan, ferulic acid and its dimers by the NO_2_^•^ radical according to the classical Marcus theory.

Combination	ΔG, kJ/mol	λ, kJ/mol	H_AB_	lgk_ET_(NO_2_^•^)
FA	−60.74	251.75	0.647	9.18
XII	−110.64	267.23	0.018	8.43
XIII	−88.48	251.98	0.075	9.04
XIV	−73.10	267.03	0.122	7.90
XV	−72.60	254.95	0.074	7.94

Notes: ΔG is the Gibbs energy of the oxidation reaction, kJ/mol; λ is the reorganization energy, kJ/mol; H_AB_ is the matrix element of the bond.

## Data Availability

The original contributions presented in this study are included in the article/Supplementary Materials. Further inquiries can be directed to the corresponding author.

## Supplementary Materials

The following supporting information can be downloaded at https://www.mdpi.com/article/10.3390/polym18172167/s1. S1 Description of the conditions for registration of XPS spectra. S2 Cartesian coordinates of the structure of the molecular complex of honey with glucosamine and ferulic acid. Figure S1. The structure of the molecular complex of glucosamine and the ferulic acid radical, determined by the molecular docking method (GFN2-xTB quantum chemical modeling method). Figure S2. Micrography of chitosan modified with ferulic acid in the presence of ScaSL laccase (130 U/g chitosan) at pH 7.0 with a ratio of n(FA)/n(NH2) = 1:10.

## References

[1] Kaur K., Sharma A., Capalash N., Sharma P. (2019). Multicopper Oxidases: Biocatalysts in Microbial Pathogenesis and Stress Management. Microbiol. Res..

[2] Janusz G., Pawlik A., Świderska-Burek U., Polak J., Sulej J., Jarosz-Wilkołazka A., Paszczyński A. (2020). Laccase Properties, Physiological Functions, and Evolution. Int. J. Mol. Sci..

[3] Bai Y., Ali S., Liu S., Zhou J., Tang Y. (2023). Characterization of Plant Laccase Genes and Their Functions. Gene.

[4] Loi M., Glazunova O., Fedorova T., Logrieco A.F., Mulè G. (2021). Fungal Laccases: The Forefront of Enzymes for Sustainability. J. Fungi.

[5] Zavarzina A.G., Kulikova N.A., Trubitsina L.I., Belova O.V., Pyatova M.I., Danilin I.V., Pogozhev P.E., Kuzyakov Y., Lisov A.V. (2025). Disentangling Two and Three Domain Laccases in Soils: Contribution of Fungi, Bacteria and Abiotic Processes to Oxidative Activities. Soil Biol. Biochem..

[6] Lin H., Yu Z., Wang Q., Liu Y., Jiang L., Xu C., Xian M. (2023). Application of Laccase Catalysis in Bond Formation and Breakage: A Review. Catalysts.

[7] Mahuri M., Paul M., Thatoi H. (2023). A Review of Microbial Laccase Production and Activity toward Different Biotechnological Applications. Syst. Microbiol. Biomanuf.

[8] Khatami S.H., Vakili O., Movahedpour A., Ghesmati Z., Ghasemi H., Taheri-Anganeh M. (2022). Laccase: Various Types and Applications. Biotech. App Biochem..

[9] Yu Y., Xing Y., Liu F., Zhang X., Li X., Zhang J., Sun X. (2021). The Laccase Gene Family Mediate Multi-Perspective Trade-Offs during Tea Plant (Camellia Sinensis) Development and Defense Processes. Int. J. Mol. Sci..

[10] Hu Q., Min L., Yang X., Jin S., Zhang L., Li Y., Ma Y., Qi X., Li D., Liu H. (2018). Laccase GhLac1 Modulates Broad-Spectrum Biotic Stress Tolerance via Manipulating Phenylpropanoid Pathway and Jasmonic Acid Synthesis. Plant Physiol..

[11] Carunchio F. (2001). Oxidation of Ferulic Acid by Laccase: Identification of the Products and Inhibitory Effects of Some Dipeptides. Talanta.

[12] Aljawish A., Chevalot I., Jasniewski J., Paris C., Scher J., Muniglia L. (2014). Laccase-Catalysed Oxidation of Ferulic Acid and Ethyl Ferulate in Aqueous Medium: A Green Procedure for the Synthesis of New Compounds. Food Chem..

[13] Adelakun O.E., Kudanga T., Parker A., Green I.R., Le Roes-Hill M., Burton S.G. (2012). Laccase-Catalyzed Dimerization of Ferulic Acid Amplifies Antioxidant Activity. J. Mol. Catal. B Enzym..

[14] Robert B., Chenthamara D., Subramaniam S. (2022). Fabrication and Biomedical Applications of Arabinoxylan, Pectin, Chitosan, Soy Protein, and Silk Fibroin Hydrogels via Laccase—Ferulic Acid Redox Chemistry. Int. J. Biol. Macromol..

[15] Boundaoui K., Le Cerf D., Dulong V. (2024). Functionalisation and Behaviours of Polysaccharides Conjugated with Phenolic Compounds by Oxidoreductase Catalysis: A Review. Int. J. Biol. Macromol..

[16] Backes E., Kato C.G., Corrêa R.C.G., Moreira R.d.F.P.M., Peralta R.A., Barros L., Ferreira I.C.F.R., Zanin G.M., Bracht A., Peralta R.M. (2021). Laccases in Food Processing: Current Status, Bottlenecks and Perspectives. Trends Food Sci. Technol..

[17] Upadhyay P., Shrivastava R., Agrawal P.K. (2016). Bioprospecting and Biotechnological Applications of Fungal Laccase. 3 Biotech.

[18] Zerva A., Simić S., Topakas E., Nikodinovic-Runic J. (2019). Applications of Microbial Laccases: Patent Review of the Past Decade (2009–2019). Catalysts.

[19] Huber D., Tegl G., Baumann M., Sommer E., Gorji E.G., Borth N., Schleining G., Nyanhongo G.S., Guebitz G.M. (2017). Chitosan Hydrogel Formation Using Laccase Activated Phenolics as Cross-Linkers. Carbohydr. Polym..

[20] Aljawish A., Chevalot I., Piffaut B., Rondeau-Mouro C., Girardin M., Jasniewski J., Scher J., Muniglia L. (2012). Functionalization of Chitosan by Laccase-Catalyzed Oxidation of Ferulic Acid and Ethyl Ferulate under Heterogeneous Reaction Conditions. Carbohydr. Polym..

[21] Aljawish A., Chevalot I., Jasniewski J., Revol-Junelles A.-M., Scher J., Muniglia L. (2014). Laccase-Catalysed Functionalisation of Chitosan by Ferulic Acid and Ethyl Ferulate: Evaluation of Physicochemical and Biofunctional Properties. Food Chem..

[22] Kaur R., Salwan R., Sharma V. (2022). Structural Properties, Genomic Distribution of Laccases from *Streptomyces* and Their Potential Applications. Process Biochem..

[23] Chauhan P.S., Goradia B., Saxena A. (2017). Bacterial Laccase: Recent Update on Production, Properties and Industrial Applications. 3 Biotech.

[24] Trubitsina L.I., Trubitsin I.V., Lisov A.V., Gabdulkhakov A.G., Zavarzina A.G., Belova O.V., Larionova A.P., Tishchenko S.V., Leontievsky A.A. (2023). A Novel Two-Domain Laccase with Middle Redox Potential: Physicochemical and Structural Properties. Biochem. Mosc..

[25] Fu P., Zhou D., Li W.-L., Lin W.-T., Huang X.-J., Xu Z.-K., Wan L.-S. (2023). Laccase-Triggered One-Step Fabrication of Positively Charged Phenolic Acid-Amine Networks for Nanofiltration. Desalination.

[26] Dmitruk A.R., Oskin P.V., Trubitsina L.I., Yushkin A.A., Alferov V.A., Leontievskiy A.A., Ponamoreva O.N. (2025). Cross-Linking of Chitosan with Ferulic Acid in the Presence of Bacterial Laccase: Investigation of the Mechanism—The Michael Addition Reaction or the Formation of Schiff Bases?. Кинетика Катализ Kinet. Catal..

[27] Hammond G.S. (1955). A Correlation of Reaction Rates. J. Am. Chem. Soc..

[28] Campanella L., Antiochia R., Dragone R., Lavagnini I. (2005). Determination of Oxygen Permeability of Food Wrapping Films by an Amperometric Sensor. Int. J. Environ. Anal. Chem..

[29] Singleton V.L., Orthofer R., Lamuela-Raventós R.M. (1999). [14] Analysis of Total Phenols and Other Oxidation Substrates and Antioxidants by Means of Folin-Ciocalteu Reagent. Methods in Enzymology.

[30] Santana W.E.L., Nunez C.V., Moya H.D. (2015). Antioxidant Activity and Polyphenol Content of Some Brazilian Medicinal Plants Exploiting the Formation of the Fe(II)/2,2′-Bipyridine Complexes. Nat. Prod. Commun..

[31] Bannwarth C., Ehlert S., Grimme S. (2019). GFN2-xTB—An Accurate and Broadly Parametrized Self-Consistent Tight-Binding Quantum Chemical Method with Multipole Electrostatics and Density-Dependent Dispersion Contributions. J. Chem. Theory Comput..

[32] Bannwarth C., Caldeweyher E., Ehlert S., Hansen A., Pracht P., Seibert J., Spicher S., Grimme S. (2021). Extended tight-binding Quantum Chemistry Methods. WIREs Comput. Mol. Sci..

[33] Tan C., Yang L., Luo R. (2006). How Well Does Poisson−Boltzmann Implicit Solvent Agree with Explicit Solvent? A Quantitative Analysis. J. Phys. Chem. B.

[34] Plett C., Grimme S. (2023). Automated and Efficient Generation of General Molecular Aggregate Structures. Angew. Chem. Int. Ed..

[35] Grimme S. (2019). Exploration of Chemical Compound, Conformer, and Reaction Space with Meta-Dynamics Simulations Based on Tight-Binding Quantum Chemical Calculations. J. Chem. Theory Comput..

[36] Kohn J.T., Gildemeister N., Grimme S., Fazzi D., Hansen A. (2023). Efficient Calculation of Electronic Coupling Integrals with the Dimer Projection Method via a Density Matrix Tight-Binding Potential. J. Chem. Phys..

[37] Vårum K.M., Ottøy M.H., Smidsrød O. (1994). Water-Solubility of Partially N-Acetylated Chitosans as a Function of pH: Effect of Chemical Composition and Depolymerisation. Carbohydr. Polym..

[38] Kulikov S.N., Bayazitova L.T., Tyupkina O.F., Zelenikhin P.V., Salnikova M.M., Bezrodnykh E.A., Tikhonov V.E. (2016). Evaluation of a Method for the Determination of Antibacterial Activity of Chitosan. Appl. Biochem. Microbiol..

[39] Kulikov S.N., Tikhonov V.E., Bezrodnykh E.A., Lopatin S.A., Varlamov V.P. (2015). Comparative Evaluation of Antimicrobial Activity of Oligochitosans against Klebsiella Pneumoniae. Russ. J. Bioorganic Chem..

[40] Shiekh P.A., Singh A., Kumar A. (2020). Exosome Laden Oxygen Releasing Antioxidant and Antibacterial Cryogel Wound Dressing OxOBand Alleviate Diabetic and Infectious Wound Healing. Biomaterials.

[41] Khan S., Ranjha N.M. (2014). Effect of Degree of Cross-Linking on Swelling and on Drug Release of Low Viscous Chitosan/Poly(Vinyl Alcohol) Hydrogels. Polym. Bull..

[42] Giannakopoulou A., Tsapara G., Troganis A.N., Koralli P., Chochos C.L., Polydera A.C., Katapodis P., Barkoula N.-M., Stamatis H. (2022). Development of a Multi-Enzymatic Approach for the Modification of Biopolymers with Ferulic Acid. Biomolecules.

[43] Yang J., Sun J., An X., Zheng M., Lu Z., Lu F., Zhang C. (2018). Preparation of Ferulic Acid-Grafted Chitosan Using Recombinant Bacterial Laccase and Its Application in Mango Preservation. RSC Adv..

[44] Troe J. (2004). Limitations of Variational Transition State Theory for Barrierless Radical–Radical Recombination Reactions. Z. Für Phys. Chem..

[45] Kulik T., Nastasiienko N., Palianytsia B., Ilchenko M., Larsson M. (2021). Catalytic Pyrolysis of Lignin Model Compound (Ferulic Acid) over Alumina: Surface Complexes, Kinetics, and Mechanisms. Catalysts.

[46] SriBala G., Van De Vijver R., Li L., Dogu O., Marin G.B., Van Geem K.M. (2021). On the Primary Thermal Decomposition Pathways of Hydroxycinnamic Acids. Proc. Combust. Inst..

[47] Corazzari I., Nisticò R., Turci F., Faga M.G., Franzoso F., Tabasso S., Magnacca G. (2015). Advanced Physico-Chemical Characterization of Chitosan by Means of TGA Coupled on-Line with FTIR and GCMS: Thermal Degradation and Water Adsorption Capacity. Polym. Degrad. Stab..

[48] Kittur F.S., Harish Prashanth K.V., Udaya Sankar K., Tharanathan R.N. (2002). Characterization of Chitin, Chitosan and Their Carboxymethyl Derivatives by Differential Scanning Calorimetry. Carbohydr. Polym..

[49] Kirsch M., Korth H.-G., Sustmann R., Groot H.D. (2002). The Pathobiochemistry of Nitrogen Dioxide. Biol. Chem..

[50] Song J., Zhou H., Gao R., Zhang Y., Zhang H., Zhang Y., Wang G., Wong P.K., Zhao H. (2018). Selective Determination of Cr(VI) by Glutaraldehyde Cross-Linked Chitosan Polymer Fluorophores. ACS Sens..

[51] Stevie F.A., Donley C.L. (2020). Introduction to X-Ray Photoelectron Spectroscopy. J. Vac. Sci. Technol. A Vac. Surf. Films.

[52] Amaral I.F., Granja P.L., Barbosa M.A. (2005). Chemical Modification of Chitosan by Phosphorylation: An XPS, FT-IR and SEM Study. J. Biomater. Sci. Polym. Ed..

[53] Gorham J. (2012). NIST X-Ray Photoelectron Spectroscopy Database—SRD 20.

[54] Dai X., Liu X., Li Y., Xu Q., Yang L., Gao F. (2023). Nitrogen-Phosphorous Co-Doped Carbonized Chitosan Nanoparticles for Chemotherapy and ROS-Mediated Immunotherapy of Intracellular Staphylococcus Aureus Infection. Carbohydr. Polym..

[55] Kaczmarek-Szczepańska B., Wekwejt M., Mazur O., Zasada L., Pałubicka A., Olewnik-Kruszkowska E. (2021). The Physicochemical and Antibacterial Properties of Chitosan-Based Materials Modified with Phenolic Acids Irradiated by UVC Light. Int. J. Mol. Sci..

[56] Graham M., Klinge S. (2024). Multiscale Homogenisation of Diffusion in Enzymatically-Calcified Hydrogels. J. Mech. Behav. Biomed. Mater..

[57] Zhang D., Zhou W., Wei B., Wang X., Tang R., Nie J., Wang J. (2015). Carboxyl-Modified Poly(Vinyl Alcohol)-Crosslinked Chitosan Hydrogel Films for Potential Wound Dressing. Carbohydr. Polym..

[58] Jayakumar R., Prabaharan M., Sudheesh Kumar P.T., Nair S.V., Tamura H. (2011). Biomaterials Based on Chitin and Chitosan in Wound Dressing Applications. Biotechnol. Adv..

[59] Şalva E., Akdağ A.E., Alan S., Arısoy S., Akbuğa F.J. (2023). Evaluation of the Effect of Honey-Containing Chitosan/Hyaluronic Acid Hydrogels on Wound Healing. Gels.

[60] Głąb M., Drabczyk A., Kudłacik-Kramarczyk S., Krzan M., Tyliszczak B. (2021). Physicochemical Characteristics of Chitosan-Based Hydrogels Modified with *Equisetum arvense* L. (Horsetail) Extract in View of Their Usefulness as Innovative Dressing Materials. Materials.

[61] Chang K.L.B., Tai M.-C., Cheng F.-H. (2001). Kinetics and Products of the Degradation of Chitosan by Hydrogen Peroxide. J. Agric. Food Chem..

[62] Božič M., Štrancar J., Kokol V. (2013). Laccase-Initiated Reaction between Phenolic Acids and Chitosan. React. Funct. Polym..

[63] Tan W., Li Q., Zhou T., Chen Q., Wang G., Dong F., Guo Z. (2017). Synthesis and Antioxidant Ability of 6,6′-Diamino-6,6′-Dideoxytrehalose. Bioorg. Chem..

[64] Stratil P., Klejdus B., Kubáň V. (2006). Determination of Total Content of Phenolic Compounds and Their Antioxidant Activity in Vegetables—Evaluation of Spectrophotometric Methods. J. Agric. Food Chem..

[65] Prior R.L., Wu X., Schaich K. (2005). Standardized Methods for the Determination of Antioxidant Capacity and Phenolics in Foods and Dietary Supplements. J. Agric. Food Chem..

[66] Petrovic M., Suznjevic D., Pastor F., Veljovic M., Pezo L., Antic M., Gorjanovic S. (2016). Antioxidant Capacity Determination of Complex Samples and Individual Phenolics—Multilateral Approach. Comb. Chem. High Throughput Screen..

[67] Villagran M., Ferreira J., Martorell M., Mardones L. (2021). The Role of Vitamin C in Cancer Prevention and Therapy: A Literature Review. Antioxidants.

[68] Marcus R.A. (1956). On the Theory of Oxidation-Reduction Reactions Involving Electron Transfer. J. Chem. Phys..

[69] Byun J., Mueller D.M., Fabjan J.S., Heinecke J.W. (1999). Nitrogen Dioxide Radical Generated by the Myeloperoxidase-hydrogen Peroxide-nitrite System Promotes Lipid Peroxidation of Low Density Lipoprotein. FEBS Lett..

[70] Roder M., Földiák G., Wojnárovits L. (1999). Electron Transfer from Cresols to N3, BrO2, ClO2, NO2 and SO−4 Radicals: Correlation between Rate Constants and One-Electron Reduction Potentials. Radiat. Phys. Chem..

[71] Zhouen Z., Side Y., Weizhen L., Wenfeng W., Yizun J., Nianyun L. (1998). Mechanism of Reaction of Nitrogen Dioxide Radical with Hydroxycinnamic Acid Derivatives: A Pulse Radiolysis Study. Free Radic. Res..

[72] Suga K., Aoyagui S. (1973). The Applicability of the Theory of R. A. Marcus to the Electron-Transfer Reactions between Polycyclic Aromatic Hydrocarbons and Their Anion Radicals. Bull. Chem. Soc. Jpn..

