# Supplementary material for: Efficient Chitosan–Ferulic Acid Hydrogel Formation at Neutral pH by a Two-Domain Bacterial Laccase: Mechanistic and Functional Study
Source: Polymers (Basel). 2026 Sep 5;18(17):2167. doi: 10.3390/polym18172167 (PMC13568245; doi:10.3390/polym18172167)
Supplement: Supplementary file 1 [file polymers-18-02167-s001.zip › polymers-4485103-supplementary.pdf]

## S1 Description of the conditions for registration of XPS spectra

The film sample was placed on a vacuum-compatible conductive adhesive tape fixed on a copper table and installed in the device without further preparation, after which the sample was evacuated. Photoelectron spectra were obtained from a sample previously degassed in vacuum using an upgraded electronic spectrometer ES – 2403 of the SKB AP RAS, equipped with a PHOIBOS 100-5MCD energy analyzer (manufactured by Specs GmbH, Germany) and an MgKa/AlKa XR-50 X-ray source (manufactured by Specs GmbH, Germany). The characteristic Mg Ka radiation with a power of 250 watts was used for photoelectronic excitation. The spectra were recorded at a pressure of no more than 2*10-6 Pa. The overview spectrum was obtained in the range of 1100-0 eV with an energy step of 0.5 eV and a shutter speed of 0.4 s; the transmission energy of the analyzer was 40 eV (Ag 3d5/2 PSHPV 1.4 eV). High-resolution spectra of the photoelectronic bands were obtained in energy increments of 0.05 eV; the transmission energy of the analyzer was 7 eV, which corresponds to 0.85 eV of the photovoltaic band of the Ag 3d5/2 standard. The spectra were obtained using the standard SpecsLab2 software. The C 1s photoelectronic band with a reference value of 284.8 eV was used as a reference band.The CasaXPS software package was used to analyze the spectra. The analysis area is 5.6 $mm^2$.

## S2 Cartesian coordinates of the structure of the molecular complex of honey with glucosamine and ferulic acid

| | | | |
|---|---|---|---|
| O | -0.67957824419494 | 0.56932184652353 | -1.14665052972285 |
| O | -0.88276950533837 | -1.37904586941938 | 2.52693662069170 |
| O | 1.50912210792524 | -1.48452378034461 | 0.90367613232510 |
| O | -2.62619405106504 | 1.65238962971504 | -0.75744382183321 |
| O | 1.73307313518800 | 0.42052867226339 | -2.75224824260960 |
| N | -2.66033609873925 | 0.88369371112711 | 1.96668278367424 |
| C | -0.58263417471903 | -0.42328483059099 | 1.53725001543423 |
| C | 0.27418706122606 | -1.02793276987334 | 0.41522536575609 |
| C | -1.85625217745959 | 0.21177952098129 | 0.95606539556447 |
| C | 0.53765106896518 | 0.03864121805090 | -0.65550420298137 |
| C | -1.47370549875398 | 1.19528309927948 | -0.16008333818345 |
| C | 1.29945274894766 | -0.55636822386759 | -1.84459342662505 |
| H | 0.01180824069415 | 0.34121656155278 | 2.05451900338888 |
| H | -0.28345001582985 | -1.85721802655908 | -0.05205957821951 |
| H | -2.48029396639364 | -0.57078585697180 | 0.50343739988138 |
| H | 1.14145040541957 | 0.84497492624129 | -0.20935465201449 |
| H | -0.88610803169654 | 2.03133148835884 | 0.28306487035036 |
| H | 0.65329771869521 | -1.29188576682728 | -2.33858627946623 |
| H | 2.19392480285767 | -1.05244082119832 | -1.46522450606927 |
| H | -2.21037665237965 | 1.74155136341356 | 2.26928377211798 |
| H | -2.74857502697709 | 0.27179987024346 | 2.77327424048723 |
| H | -1.46740990861784 | -2.05725549977540 | 2.15636374318633 |
| H | 1.35526284215825 | -1.95596760862829 | 1.73847957968496 |
| H | -2.41387831434233 | 2.19770510737789 | -1.53006905907196 |
| H | 0.95631405571373 | 0.86416199176427 | -3.12190825946823 |
| O | -11.31963187650804 | 1.10660904409586 | -2.49130702736091 |
| O | -12.01026559227937 | -1.35178567328363 | -3.87085232904239 |
| O | -5.09638334452541 | 1.13305470532018 | 0.89030845676579 |
| O | -4.70562041214653 | -0.97187182254151 | 0.24348445366826 |
| C | -8.46404173490810 | -0.98714058605901 | -1.80796450064260 |
| C | -10.56527590148668 | 0.00279942896859 | -2.53574088335319 |
| C | -9.36136241877261 | 0.07836233840876 | -1.85452590046815 |
| C | -8.81034037135470 | -2.18760328180982 | -2.48805368525641 |
| C | -10.93852639185535 | -1.22953903498637 | -3.24290765778411 |
| C | -9.97839631811502 | -2.30226850069520 | -3.16570861159677 |
| C | -7.21313620528407 | -0.92949131559470 | -1.10673575947560 |
| C | -6.71541718118562 | 0.11528742215246 | -0.41903821656688 |
| C | -12.58087820546053 | 1.20032904795254 | -3.13012762993345 |

| | | | |
|---|---|---|---|
| C | -5.41131804232297 | 0.02813239335605 | 0.25714074295007 |
| H | -9.14227196648094 | 1.00706339401231 | -1.35320092429516 |
| H | -8.11990680370776 | -3.01658796994391 | -2.45605943027036 |
| H | -10.24650149478780 | -3.21041876967935 | -3.68347615138272 |
| H | -6.60500011838671 | -1.82594698876514 | -1.13529885969303 |
| H | -7.21778666589091 | 1.06275480923111 | -0.31584797156945 |
| H | -13.27858720257957 | 0.45737523480212 | -2.73977075573600 |
| H | -12.49089942835762 | 1.05488010518872 | -4.20803973004400 |
| H | -12.93052191721117 | 2.20639273655378 | -2.90824377099675 |
| H | -4.16828429791576 | 1.09347173469578 | 1.32287686111019 |

Transition state IV:

| | | | |
|---|---|---|---|
| O | -2.35410765427096 | 0.15782714738650 | -2.09615317345674 |
| O | -2.92625644067166 | -0.53594174751267 | 1.91322520253330 |
| O | -0.33101262685917 | -0.77519544458952 | 0.82349965945434 |
| O | -4.39218811159585 | 1.05228826376693 | -2.38317013573944 |
| O | 0.11883184177118 | -0.06124361654614 | -3.42042371069067 |
| N | -4.73453131345677 | 1.22058945182961 | 0.51631737615023 |
| C | -2.51233014627815 | 0.12269433330074 | 0.74003783984101 |
| C | -1.45549786878353 | -0.69188919101921 | -0.01888655947487 |
| C | -3.76427499654198 | 0.36121548354632 | -0.12628209482447 |
| C | -1.15652322928113 | 0.01946717517329 | -1.34991785796555 |
| C | -3.34572479717841 | 0.95302447338395 | -1.48541925459316 |
| C | -0.18692339958204 | -0.76256274416349 | -2.24433332157677 |
| H | -2.05897378574564 | 1.09500332213648 | 1.00014213595911 |
| H | -1.86872037614397 | -1.68796101713513 | -0.23006653453802 |
| H | -4.24459390781927 | -0.61048696163580 | -0.29653574766391 |
| H | -0.73399246503415 | 1.01397509943138 | -1.13696042985933 |
| H | -2.96978348422352 | 1.97948980388247 | -1.33452031243882 |
| H | -0.63404579532113 | -1.73688801180817 | -2.48056367501484 |
| H | 0.76328358539995 | -0.90456455587434 | -1.72845115134883 |
| H | -4.36165506752485 | 2.15959410053857 | 0.60956550095515 |
| H | -4.90366646906870 | 0.86059768361084 | 1.45049932364790 |
| H | -2.13622071649085 | -0.76558537108098 | 2.42524173477513 |
| H | 0.27470635169662 | -1.46296369571688 | 0.51700583799522 |
| H | -4.58368807388581 | 0.16429356221087 | -2.71888639046389 |
| H | -0.71063373818617 | 0.15607426270261 | -3.87049355077875 |
| O | -7.75790496620262 | 2.06371421607527 | -1.99435064709029 |
| O | -7.76364953417716 | 0.55443955090120 | -4.47127445174832 |
| O | -6.20668588001232 | -1.53233929917016 | 3.87396046217653 |
| O | -5.80731609252875 | -3.38284745638761 | 2.68825513627299 |
| C | -6.95835135338842 | -1.25950501396802 | -0.86459079476374 |
| C | -7.50726433001484 | 0.76095069912877 | -2.10328320898837 |
| C | -7.26854203107533 | 0.09680885459512 | -0.90907851928464 |
| C | -6.92952773875479 | -1.98761476245691 | -2.08809986845064 |
| C | -7.50401973024596 | 0.02103355824327 | -3.37417859185707 |
| C | -7.19468091650363 | -1.38442180755610 | -3.27204811739544 |
| C | -6.61834203424352 | -1.94324854669246 | 0.34933500130978 |
| C | -6.62645756792557 | -1.42880755900346 | 1.59514920667367 |
| C | -7.86742166201995 | 2.91508951805590 | -3.11977802206677 |
| C | -6.17879647031638 | -2.22168711945032 | 2.73602853480542 |
| H | -7.27466339035379 | 0.69285392955876 | -0.01334111351977 |
| H | -6.69497942587240 | -3.04112974655035 | -2.05776074400511 |
| H | -7.18226201283798 | -1.93210338158347 | -4.20208502784482 |
| H | -6.30420272172612 | -2.97615369813554 | 0.25162344436684 |
| H | -6.93552438750371 | -0.42241822077121 | 1.82080543140652 |
| H | -8.72423377193791 | 2.64629857061312 | -3.74069031101297 |
| H | -6.96549474202850 | 2.86584020281498 | -3.73282511499740 |
| H | -7.99396814439309 | 3.91291321753893 | -2.70539107642269 |
| H | -5.89278131608751 | -2.05908454692694 | 4.63275126142557 |

Transition state V:

| | | | |
|---|---|---|---|
| O | -1.44938246207031 | -0.87525222103928 | -2.23903128353581 |
| O | -3.05399563432724 | 0.65114251165483 | 1.24050635098092 |
| O | -0.23316584314388 | 0.56725443058331 | 0.92125879413707 |
| O | -3.48963477286322 | -0.91410814411791 | -3.20791859343441 |
| O | 1.35811463256212 | -1.11769124019716 | -2.70270637431405 |
| N | -4.63077595551291 | 0.62976076921877 | -0.97234367430908 |
| C | -2.40053126758828 | 0.59502795367790 | 0.00018816402671 |
| C | -1.06711770065969 | -0.16707350258632 | 0.06071120721000 |
| C | -3.34952071855904 | -0.10339975797953 | -0.97823845767642 |
| C | -0.50379276311605 | -0.26637291556490 | -1.37252201938479 |
| C | -2.67839760669277 | -0.19342244955095 | -2.36205204768400 |
| C | 0.76482466576592 | -1.12831162180139 | -1.43182191009631 |
| H | -2.17646691718697 | 1.61576893924353 | -0.35152008485482 |
| H | -1.25273028672855 | -1.17750976730832 | 0.44788308043728 |
| H | -3.53591258223155 | -1.12305714869564 | -0.62810823269560 |
| H | -0.26905318504827 | 0.74682185665330 | -1.73395631415790 |
| H | -2.48667400468495 | 0.83019993416133 | -2.74913765497340 |
| H | 0.51042437467226 | -2.15078909797934 | -1.12720349459243 |
| H | 1.51503719819409 | -0.71660766959229 | -0.75558141057501 |
| H | -4.52595165544201 | 1.53799414967436 | -1.44255802476840 |
| H | -4.83297407703838 | 0.81391894518179 | 0.02347895956112 |
| H | -2.42796762186021 | 0.95078439239240 | 1.91492473309789 |
| H | 0.50386511277256 | 0.02653932676102 | 1.23187376527345 |
| H | -3.02520840535475 | -1.13742376329067 | -4.02815749239536 |
| H | 0.74041337344282 | -1.50642313928188 | -3.33740358164270 |
| O | -9.81211324615546 | 1.43166996682124 | -1.17507214153723 |
| O | -8.12263770436325 | 2.28234371962680 | -3.41358413857675 |
| O | -7.20185811357816 | -3.31140070086276 | 3.35807078787681 |
| O | -5.28446411389889 | -3.38505027716133 | 2.19821782026263 |
| C | -6.80835081371964 | -0.47889384330034 | -0.51942647907816 |
| C | -8.55300374818727 | 0.99455617499463 | -1.42596845414811 |
| C | -8.08008283032585 | 0.08903544634699 | -0.49600650827365 |
| C | -5.86854948331461 | -0.05587352063387 | -1.60757581310325 |
| C | -7.71849816571323 | 1.43615993640811 | -2.55452992738981 |
| C | -6.44927122928437 | 0.87745126357536 | -2.59350179645925 |
| C | -6.36146305267917 | -1.43044123233844 | 0.40918017756579 |
| C | -7.02584694458465 | -1.92643772510024 | 1.50062941427020 |
| C | -10.52925924630019 | 2.22441350072746 | -2.10281080388452 |
| C | -6.41287302872032 | -2.91097847068894 | 2.33734513590560 |
| H | -8.77117532089163 | -0.19660931518101 | 0.28206718363495 |
| H | -5.46277069618422 | -0.93134587934905 | -2.12674453698908 |
| H | -5.81940249288154 | 1.16458084149000 | -3.42221375234352 |
| H | -5.37373704431030 | -1.84930924493830 | 0.26463882382657 |
| H | -8.01852333215326 | -1.61914990169984 | 1.78139592561455 |
| H | -10.65626918112984 | 1.70019959727480 | -3.05191220273258 |
| H | -10.02165880840806 | 3.16977508625331 | -2.29803299914067 |
| H | -11.49578293037526 | 2.39709360078215 | -1.63265741478233 |
| H | -6.75017805355438 | -3.98036046681599 | 3.90213676358661 |

Transitional state VI:

| | | | |
|---|---|---|---|
| O | -0.95568343781475 | 0.47708650704977 | -1.59996942293783 |
| O | -1.17666346427200 | 2.05036510641461 | 2.19190789022479 |
| O | 0.21648351619728 | 3.46878006319053 | 0.18343828100354 |
| O | -2.68799026773503 | -0.94221233909892 | -1.27251312038925 |
| O | 0.53572905049090 | 1.62950893639911 | -3.71423218610993 |
| N | -3.21275647251200 | 0.21715271082969 | 1.30083650432871 |
| C | -1.42626510685657 | 1.89948948889811 | 0.81662133849093 |
| C | -0.15148657352893 | 2.12446978813092 | -0.01044628952208 |
| C | -1.98384402345382 | 0.48215686390639 | 0.58606139194077 |
| C | -0.43809684422503 | 1.79157923202816 | -1.48654910136543 |
| C | -2.18506930259079 | 0.28780495615020 | -0.92592949009631 |
| C | 0.82427417299018 | 1.83186273381342 | -2.35640190632140 |
| H | -2.17643671001012 | 2.64003511407307 | 0.48911732703462 |

| | | | |
|---|---|---|---|
| H | 0.62639495080803 | 1.44324054944348 | 0.36241544968407 |
| H | -1.23409717712847 | -0.23751452450084 | 0.93616058589738 |
| H | -1.17389497292936 | 2.51339231748204 | -1.87734135189447 |
| H | -2.91372088283067 | 1.02911726375742 | -1.29357296621130 |
| H | 1.52344843388242 | 1.06792891493802 | -1.99186129537387 |
| H | 1.28724456115654 | 2.81775030245679 | -2.29723676916740 |
| H | -3.94852008799523 | 0.83110954706720 | 0.96528130882875 |
| H | -3.06248387953436 | 0.42292190532617 | 2.28308748075725 |
| H | -0.74527968837042 | 2.90636564610532 | 2.33355912254674 |
| H | 1.11939293681366 | 3.62380065148281 | -0.12419155579293 |
| H | -2.08915193625183 | -1.63998249135096 | -0.96140785017372 |
| H | 0.03783399696744 | 0.80390888234346 | -3.80338782901162 |
| O | -5.48837389286570 | 2.12943169133320 | -1.04851099270349 |
| O | -4.67673466043176 | 1.00089997528201 | -3.58850572388652 |
| O | -8.51215081770518 | -2.33313580022880 | 3.52836618898108 |
| O | -8.29132738074119 | -4.02908079835642 | 2.09149308744098 |
| C | -6.43189288789477 | -1.33680104573622 | -0.69249394039260 |
| C | -5.65007180468516 | 0.84694419529579 | -1.41718313634689 |
| C | -6.22748779013349 | 0.02478237830132 | -0.46843475916876 |
| C | -6.01885847855184 | -1.88347572685720 | -1.93868867866257 |
| C | -5.21735964122319 | 0.30017561987313 | -2.71019951284748 |
| C | -5.44689560681696 | -1.11107736884980 | -2.89274705203012 |
| C | -7.04734700974839 | -2.20083871756840 | 0.27049484141174 |
| C | -7.51486945432083 | -1.85617854709177 | 1.48785941303607 |
| C | -5.06458262393460 | 3.13523406122020 | -1.95283098728137 |
| C | -8.12980900219111 | -2.85186101023148 | 2.36448357359729 |
| H | -6.52145245226605 | 0.47889072189668 | 0.46379122103721 |
| H | -6.16819830229727 | -2.93759573942042 | -2.11445471855481 |
| H | -5.12746383956205 | -1.51896762426750 | -3.83889540967510 |
| H | -7.15255513575701 | -3.24116859227972 | -0.01342497449951 |
| H | -7.46897390496733 | -0.85812513999168 | 1.89044253409327 |
| H | -5.71710312530439 | 3.18339417069978 | -2.82575992882255 |
| H | -4.04377748964084 | 2.95894391844604 | -2.29997258372379 |
| H | -5.11769794377209 | 4.06177632667662 | -1.38461738322101 |
| H | -8.91969440535028 | -2.99820592428791 | 4.11489147308477 |

Transitional state VII

| | | | |
|---|---|---|---|
| O | -0.63153817259514 | 0.24727717926514 | -2.68594441155754 |
| O | -3.22975039660443 | -1.22814483431769 | 0.17660177829854 |
| O | -0.48301825814147 | -1.34115073963593 | 0.62261535250702 |
| O | -2.34383518291334 | 1.22745412534395 | -3.79949110547571 |
| O | 2.20096760674285 | 0.11712733158703 | -2.39421881598562 |
| N | -4.14505960305998 | 0.85144877602301 | -1.58991994792692 |
| C | -2.33631469243336 | -0.34706658906333 | -0.44745630548232 |
| C | -0.97511572469880 | -1.03456567586656 | -0.66019634501284 |
| C | -2.91774219306191 | 0.11234415824784 | -1.78769674637369 |
| C | -0.03736270816981 | -0.10321838199788 | -1.44636367194697 |
| C | -1.85072062112498 | 0.93883313067381 | -2.54253019535225 |
| C | 1.30830942180093 | -0.76554694196504 | -1.76751460301255 |
| H | -2.17410802995790 | 0.53687770850494 | 0.19252331201922 |
| H | -1.14267360132139 | -1.94542152995287 | -1.25104346642166 |
| H | -3.15197749714385 | -0.76659234152478 | -2.39737952925670 |
| H | 0.13797249020197 | 0.80954741556204 | -0.85382143206043 |
| H | -1.65717718139151 | 1.87392043264832 | -1.96661768668008 |
| H | 1.12551769704689 | -1.64544876247975 | -2.39690808407859 |
| H | 1.79582670031524 | -1.06575421672329 | -0.83913591174179 |
| H | -2.78396713874892 | -1.58124728777491 | 0.96332983745869 |
| H | 0.19369426431640 | -2.02909860294609 | 0.57359605838324 |
| H | -1.64202241189558 | 1.59318007386590 | -4.35943951460448 |
| H | 1.80330192804469 | 0.42130392617111 | -3.22194223894751 |
| O | -9.06437796917041 | -0.35364829933067 | -3.32134260334359 |
| O | -6.69981215635369 | -1.54919060529382 | -3.58871324097860 |
| O | -7.36185482057968 | 0.37670343835513 | 4.88668094499678 |

| | | | |
|---|---|---|---|
| O | -9.25443087711403 | 1.10876316263102 | 3.95199494867451 |
| C | -7.84471096379799 | -0.30330101628882 | 0.15113197704072 |
| C | -8.33123549912779 | -0.50848798224808 | -2.21689189238388 |
| C | -8.68777949813628 | -0.09749559767498 | -0.95210907084869 |
| C | -6.60267367850240 | -0.96069461908621 | -0.05120620780059 |
| C | -7.03881765468422 | -1.18711717955154 | -2.44941606054661 |
| C | -6.22214917967495 | -1.38708923968390 | -1.27961135942235 |
| C | -8.27437779730850 | 0.15474212694426 | 1.43646521178368 |
| C | -7.59732897894896 | 0.05333548301971 | 2.60057396801844 |
| C | -10.33045235419613 | 0.28301315571129 | -3.24157927178323 |
| C | -8.16708369956005 | 0.56515979674929 | 3.84284138738459 |
| H | -9.63153286089979 | 0.39909545847317 | -0.78686324743354 |
| H | -5.93605383099890 | -1.11748515530464 | 0.77865118841217 |
| H | -9.24570666224796 | 0.63378351942116 | 1.48357715793968 |
| H | -6.62264948795926 | -0.39626447784423 | 2.68974483493232 |
| H | -11.00448025952231 | -0.27512675940684 | -2.58855436270323 |
| H | -10.71462969252799 | 0.28320456134760 | -4.25861686352768 |
| H | -10.22642859156587 | 1.30863337588217 | -2.88176076522329 |
| H | -7.74648372915503 | 0.71679817460648 | 5.71615905500147 |
| H | -4.54723385070701 | 1.07627473645986 | -2.49261596793161 |
| H | -3.95673696592488 | 1.71889934919029 | -1.09876364846094 |
| H | -5.27333314767346 | -1.87717850405865 | -1.41990517293137 |

Transitional state VIII

| | | | |
|---|---|---|---|
| O | -0.25278593621796 | 1.31228175144583 | -3.13016269291068 |
| O | -2.16372592309683 | 0.36177521902336 | 0.40232839216167 |
| O | 0.35267633341644 | -0.77851700049362 | -0.16702080117783 |
| O | -1.58597321112664 | 3.09061031543428 | -3.53011572235563 |
| O | 2.15745963036323 | 0.18502681495582 | -3.98749218492679 |
| N | -2.84714461110657 | 2.78264232983531 | -0.95761874902852 |
| C | -1.23635355873197 | 0.93775318271391 | -0.48627169032821 |
| C | -0.38152108421932 | -0.13250886893050 | -1.17929033196613 |
| C | -2.01064757275912 | 1.75232701079480 | -1.53513234889763 |
| C | 0.52259598285667 | 0.54910237036974 | -2.22309433174600 |
| C | -1.00291182535885 | 2.34936065521292 | -2.53013877940336 |
| C | 1.30034630271475 | -0.45675011172896 | -3.08109261376054 |
| H | -0.55655876101877 | 1.60672602515440 | 0.06975401950391 |
| H | -1.05805742441914 | -0.83179929148232 | -1.69019352593042 |
| H | -2.66354285397107 | 1.05568177043078 | -2.08374313957275 |
| H | 1.23214620666727 | 1.20860663876694 | -1.69728428675242 |
| H | -0.32091438363107 | 3.03728974161369 | -1.99844985939169 |
| H | 0.57976588330377 | -1.09123877905277 | -3.61361711703365 |
| H | 1.94209335664890 | -1.07228366974523 | -2.44964319416918 |
| H | -2.30486315385225 | 3.60700029272797 | -0.72504996146937 |
| H | -3.27324175087970 | 2.42182478061730 | -0.10961709492065 |
| H | -1.67159023224759 | -0.19491443718628 | 1.02400454612507 |
| H | 0.73482070108905 | -1.60238394917866 | -0.49695282600340 |
| H | -2.16962106328500 | 2.52244384267028 | -4.05668699186029 |
| H | 1.63274696624212 | 0.80915342075174 | -4.51058210453930 |
| O | -6.22701487145765 | 1.52693130374771 | -1.19423749641831 |
| O | -4.25105370352641 | -0.06671483224413 | -2.62618182283840 |
| O | -9.24732517832026 | -1.55908951474229 | 4.41675653042848 |
| O | -8.02565057183195 | -3.40219447135675 | 4.09888081733237 |
| C | -6.20708776884066 | -1.54459893488566 | 0.68395847548765 |
| C | -5.84576906674995 | 0.28779204967932 | -0.88183298556008 |
| C | -6.47141542244949 | -0.25472011036671 | 0.23294665068443 |
| C | -5.26286778385959 | -2.32645305993358 | -0.03572777115782 |
| C | -4.86259750213624 | -0.50418903343507 | -1.62500037964626 |
| C | -4.63038777069034 | -1.83357554689601 | -1.12831282206672 |
| C | -6.84329280055397 | -2.12060417050543 | 1.83269160148229 |
| C | -7.77510312319251 | -1.54842761267792 | 2.62153875657128 |
| C | -5.69132846102143 | 2.26436893047695 | -2.27895099918394 |
| C | -8.33436630150249 | -2.27155082942744 | 3.75863601634273 |

| | | | |
|---|---|---|---|
| H | -7.18182826509300 | 0.37485524392425 | 0.74367081792956 |
| H | -5.05451040137670 | -3.32904623918389 | 0.30542562990704 |
| H | -3.91254459105621 | -2.42370092361011 | -1.67759746265934 |
| H | -6.54382280859545 | -3.12930874032118 | 2.09229644635758 |
| H | -8.16233728941384 | -0.55446652193826 | 2.47180938946485 |
| H | -5.68082823173801 | 1.67894334884910 | -3.19794555948404 |
| H | -4.66940002425196 | 2.59188933805615 | -2.04630638091769 |
| H | -6.34608545250085 | 3.12754208320805 | -2.37742029252238 |
| H | -9.61991153751174 | -2.04757575840683 | 5.17478588545482 |

Transitional state IX

| | | | |
|---|---|---|---|
| O | -0.37997238898484 | 0.58103722137745 | -0.79911965145516 |
| O | -3.21886133553748 | -2.39830279092577 | -1.57435433223185 |
| O | -1.28475545400813 | -2.70820270745586 | 0.47344039071619 |
| O | -1.36327784131937 | 1.99845684723690 | -2.26828099165343 |
| O | 1.41400943897565 | 0.25696303544452 | 1.47313455992376 |
| N | -3.60353307911855 | 0.24107516067456 | -2.65168742409873 |
| C | -2.53107096695660 | -1.28967721349105 | -1.05529633259119 |
| C | -1.15826522686969 | -1.69608073738809 | -0.49070359216158 |
| C | -2.34065408844373 | -0.15517444852858 | -2.06522882437547 |
| C | -0.51214289591680 | -0.46229780402128 | 0.15295772011762 |
| C | -1.61241861669601 | 1.00066107525118 | -1.34300927185166 |
| C | 0.88588818943974 | -0.77983177425117 | 0.68898863391942 |
| H | -3.13617554701989 | -0.92901686542732 | -0.21481475398316 |
| H | -0.50955575203696 | -2.04293118565259 | -1.31371362890468 |
| H | -1.68454689476134 | -0.49022913914810 | -2.87866267008859 |
| H | -1.15621027002016 | -0.12644705169109 | 0.98044068503714 |
| H | -2.26089080914640 | 1.36792283961994 | -0.51820987419506 |
| H | 1.54356208909127 | -0.99924726297690 | -0.16119652297275 |
| H | 0.81593426308750 | -1.65736805964727 | 1.33283460737839 |
| H | -3.43749339987649 | 0.99771573796344 | -3.30652070241667 |
| H | -4.22092757102048 | 0.59412420786865 | -1.92660707349257 |
| H | -2.87985884971041 | -2.60669860632397 | -2.45676727452930 |
| H | -1.99682649890338 | -3.30494214856780 | 0.18695227578632 |
| H | -0.73648133266068 | 2.64009920547719 | -1.90215566274127 |
| H | 1.46821950792511 | 1.05507834691711 | 0.92850465485308 |
| O | -10.69746264132134 | 0.80416180693735 | -1.40021810055548 |
| O | -10.42977891634089 | -1.32812594728967 | -3.34343792755973 |
| O | -4.98968820835406 | 2.00453517314674 | 2.66258721434198 |
| O | -3.76112499303473 | 0.68732768492787 | 1.34183085191400 |
| C | -7.31459660471807 | -0.31305398231433 | -0.85842082875113 |
| C | -9.60270853062280 | 0.06388187715988 | -1.59063967637608 |
| C | -8.52888165668673 | 0.36492704247855 | -0.76811562441021 |
| C | -7.18311833534768 | -1.35014719703163 | -1.82517003298968 |
| C | -9.48942219502023 | -1.01063116287839 | -2.58988596636301 |
| C | -8.21218691368565 | -1.67987311693446 | -2.64174633141454 |
| C | -6.17891309774321 | -0.00327109187836 | -0.04284804755051 |
| C | -6.09430174051040 | 0.93562509917907 | 0.92281023001829 |
| C | -11.87678768272961 | 0.64642228819887 | -2.16931879588357 |
| C | -4.84435598292226 | 1.17066270582190 | 1.63855144324624 |
| H | -8.66925164012733 | 1.15884181983521 | -0.05252670222351 |
| H | -6.23801890336645 | -1.86702202279198 | -1.89891232395125 |
| H | -8.12229939969027 | -2.46340863170494 | -3.37791365765333 |
| H | -5.29478830303418 | -0.59774695879205 | -0.23762600968214 |
| H | -6.92024636706038 | 1.55622832503819 | 1.22876274872809 |
| H | -12.29494829005555 | -0.35485911871822 | -2.05264010537393 |
| H | -11.68298639766451 | 0.81594178684662 | -3.22995539944924 |
| H | -12.56410016196779 | 1.39545722532273 | -1.78175641005329 |
| H | -4.15530407036918 | 2.15545410477946 | 3.14606589514249 |

Transitional state X

| | | | |
|---|---|---|---|
| O | -1.92893473708840 | 0.96298707896241 | -1.38090245381605 |
| O | -1.10771977447187 | -2.91660936325033 | -0.26005579080803 |

| | | | |
|---|---|---|---|
| O | 0.94951920295461 | -0.99369877573135 | -0.20109280745262 |
| O | -4.14471938527883 | 0.56191711658081 | -1.40463123653317 |
| O | 0.06624337907829 | 3.02418064014007 | -1.41825234929972 |
| N | -3.80796629560918 | -1.91626659048178 | -0.02772389290003 |
| C | -1.34991011760295 | -1.53558218099608 | -0.14308098531172 |
| C | -0.26536804552588 | -0.71754777619240 | -0.85701725379783 |
| C | -2.73893016725701 | -1.24564080540798 | -0.73759563998225 |
| C | -0.65086313674543 | 0.77098293044223 | -0.79882273746854 |
| C | -2.97266172516924 | 0.27278921630464 | -0.73122607916131 |
| C | 0.32989002073810 | 1.65584320841930 | -1.57882367605127 |
| H | -1.35369560374096 | -1.24269784861008 | 0.92138652676124 |
| H | -0.22889243859400 | -1.03567714034627 | -1.90823770588562 |
| H | -2.75792232012004 | -1.58969170451672 | -1.77799786741481 |
| H | -0.67609325250469 | 1.08825060808746 | 0.25639990315766 |
| H | -3.02617274938269 | 0.61496157679085 | 0.32591658500236 |
| H | 0.28788400186259 | 1.37278314062181 | -2.63810826545061 |
| H | 1.33942243663856 | 1.50615171353097 | -1.19424103834208 |
| H | -3.81845894186805 | -1.59501991519035 | 0.93874439382750 |
| H | -3.60553609334285 | -2.91051133148921 | -0.01635415272008 |
| H | -0.20766837434197 | -3.09499106648925 | 0.05145377256006 |
| H | 1.69967943859286 | -0.72036832686423 | -0.74602875274600 |
| H | -4.08784420586263 | 1.46228319473680 | -1.75997737354728 |
| H | -0.83028811905509 | 3.20652470736989 | -1.73253178951187 |
| O | -7.77447245145108 | -0.61438706276976 | -3.56023430386617 |
| O | -10.66368587493483 | -0.53463331390166 | -3.34089314659119 |
| O | -5.86565631611934 | 0.92932019623018 | 3.47875783576402 |
| O | -4.18691062263160 | -0.11076523973024 | 2.50631695144970 |
| C | -8.25722586543236 | 0.02980786274492 | -0.04964321236287 |
| C | -8.45706145735758 | -0.40272377111759 | -2.43334065055463 |
| C | -7.68501706389276 | -0.21020939686365 | -1.29732763815956 |
| C | -9.67675117790031 | 0.08498951709384 | 0.04680172832931 |
| C | -9.92504191803043 | -0.36175941965827 | -2.35052623800793 |
| C | -10.46486327027490 | -0.10266791719394 | -1.03887372463607 |
| C | -7.48089716580413 | 0.23260107811242 | 1.14209405457607 |
| C | -6.14016384282084 | 0.10895220561370 | 1.22316069900093 |
| C | -8.40103200702490 | -0.84394042603257 | -4.80899916530493 |
| C | -5.33823312901496 | 0.29400343200290 | 2.43279962018261 |
| H | -6.61365860847970 | -0.24959724546200 | -1.42133756789942 |
| H | -10.12808948021294 | 0.27455003982536 | 1.00895031358413 |
| H | -11.54137781193943 | -0.06742272289307 | -0.97218913965569 |
| H | -8.06752449476912 | 0.47029220810216 | 2.02157483208583 |
| H | -5.55816257380557 | -0.20514480962148 | 0.37053645731870 |
| H | -9.01461517349067 | 0.00833773504734 | -5.10734441170037 |
| H | -9.03767835809336 | -1.73004263071799 | -4.77687660419511 |
| H | -7.58113375300349 | -0.98841969570459 | -5.50934465843341 |
| H | -6.76734442150134 | 1.26135440680918 | 3.31758511855196 |

Transitional state XI

| | | | |
|---|---|---|---|
| O | -0.89476880086293 | 0.63489345545170 | -1.60288523346307 |
| O | -3.38545544523535 | -1.17835949998706 | 1.16286634076720 |
| O | -0.73773955108488 | -2.05312210545608 | 0.83607236686198 |
| O | -2.32462194031373 | 2.36694011209636 | -1.90786592212996 |
| O | 1.79654017785521 | -0.42099801956691 | -1.97001271838301 |
| N | -3.92334725571371 | 1.60329522713377 | 0.35074148716291 |
| C | -2.40885732442987 | -0.30687065310830 | 0.65638263438091 |
| C | -1.37007611982430 | -1.18121863449028 | -0.06292192520151 |
| C | -2.98171071745971 | 0.73068451170999 | -0.31966834099703 |
| C | -0.30166170759441 | -0.30252039028711 | -0.72161023740238 |
| C | -1.80720951243207 | 1.49933699635347 | -0.96516401297981 |
| C | 0.68172446255034 | -1.15156229477941 | -1.53307426364776 |
| H | -1.90538497533345 | 0.22234741744259 | 1.48197104158668 |
| H | -1.89085035434651 | -1.75268806104531 | -0.84749956115780 |
| H | -3.51814935742168 | 0.21301667868475 | -1.12280539404833 |

| | | | |
|---|---|---|---|
| H | 0.25063618154212 | 0.23157973886335 | 0.06829645093051 |
| H | -1.26499570889967 | 2.05779854512544 | -0.16655795861626 |
| H | 0.14397107427641 | -1.59199021796719 | -2.38110043533629 |
| H | 1.05980413386678 | -1.94552305621920 | -0.88785517019320 |
| H | -4.27674790508556 | 2.28080497010517 | -0.31609892792919 |
| H | -3.45347706425451 | 2.11317035891022 | 1.09260727817447 |
| H | -3.96897765734996 | -0.69025872173373 | 1.78123558117743 |
| H | -1.43066757979349 | -2.49916279794339 | 1.34978140564626 |
| H | -1.61361134314913 | 2.71084809491013 | -2.46984086696400 |
| H | 1.49129616270910 | 0.28625964384006 | -2.55568744872086 |
| O | -10.25249767909508 | -0.05855598492570 | -2.70490962113554 |
| O | -8.85563846546541 | -1.83285627062531 | -4.52474127691048 |
| O | -6.92255007705405 | 1.49712836804023 | 3.31925306399099 |
| O | -5.19862603041308 | 0.18213456603467 | 2.81221427916423 |
| C | -7.21058722528910 | -0.76800301656991 | -0.90349243520237 |
| C | -9.05286520787102 | -0.60583066368105 | -2.48574224222174 |
| C | -8.45810694133470 | -0.27265543017510 | -1.27940594934139 |
| C | -6.53574124150362 | -1.64442057077507 | -1.79956505898828 |
| C | -8.36689190304607 | -1.50499423580264 | -3.42495365168858 |
| C | -7.07995085877704 | -1.99068300617125 | -2.99086233993719 |
| C | -6.56959709030172 | -0.44490435989986 | 0.33719741616321 |
| C | -7.03047188162172 | 0.39316429852763 | 1.28800014911700 |
| C | -11.00335593316773 | -0.29815055870238 | -3.88214596095089 |
| C | -6.28449860535123 | 0.65656261324912 | 2.51310808516946 |
| H | -9.00873918090398 | 0.39009187686516 | -0.63187744454582 |
| H | -5.57102364187120 | -2.03176361055269 | -1.50814347471598 |
| H | -6.57348002192994 | -2.65338897607536 | -3.67570505063616 |
| H | -5.61539925681055 | -0.92440282539923 | 0.50948531098763 |
| H | -7.96494794101435 | 0.92493135551144 | 1.21043397079924 |
| H | -11.23054490398286 | -1.35935743375814 | -3.99813939866532 |
| H | -10.46482999072516 | 0.03702343812137 | -4.77033709049735 |
| H | -11.91791640503935 | 0.27620543471000 | -3.75042033812965 |
| H | -6.42633271850795 | 1.67272454064625 | 4.14107639983853 |

Transitional state XII

| | | | |
|---|---|---|---|
| O | -2.88553470199644 | 2.93924812621734 | -7.02361934862814 |
| O | -1.56723899019921 | 0.27258827967701 | -4.15029332225883 |
| O | -0.05270366781631 | 0.67932647112009 | -6.43567635123547 |
| O | -4.51425925006602 | 3.69313064708174 | -5.65494026179445 |
| O | -1.43530906444884 | 3.43377391952711 | -9.42960864233797 |
| N | -3.23250547343653 | 2.59350452517414 | -3.38354363860872 |
| C | -1.65278022936238 | 1.46418981359984 | -4.89192856724599 |
| C | -1.36673136807427 | 1.17765254154433 | -6.37565040604752 |
| C | -3.04588074051271 | 2.08556054236387 | -4.72994808845289 |
| C | -1.55995589184492 | 2.46437498586766 | -7.19350876913128 |
| C | -3.19745166615033 | 3.27459188448126 | -5.69039274307159 |
| C | -1.35770210380042 | 2.23991987189550 | -8.69755445300040 |
| H | -0.90330663579103 | 2.18308563866824 | -4.52615797068673 |
| H | -2.09277538194721 | 0.42904562024079 | -6.72471562217585 |
| H | -3.79748804705747 | 1.32924531624714 | -5.01555580194116 |
| H | -0.84855126434328 | 3.22672208080629 | -6.83832163242629 |
| H | -2.50830479135214 | 4.07608133413318 | -5.35362279728653 |
| H | -2.10664681091441 | 1.51868198294769 | -9.04860665149234 |
| H | -0.35441709440039 | 1.85158396037863 | -8.87668972580285 |
| H | -3.94693948422680 | 3.31619060707764 | -3.40006219777965 |
| H | -0.73663606803270 | -0.16217223382191 | -4.40380589388520 |
| H | 0.10739020570164 | 0.23669816588798 | -7.27949050654135 |
| H | -4.62824489596026 | 4.46736728081463 | -6.22842805852246 |
| H | -2.30988568549804 | 3.82368550177079 | -9.29256999001547 |
| O | -8.00301625644648 | 1.11807209264836 | -4.48845450623235 |
| O | -7.61530222379634 | -1.53879455474840 | -4.62253644107942 |
| O | -4.92536432643614 | 3.73158544140740 | 0.36401738584790 |
| O | -5.33367682030364 | 3.93887976393742 | -1.80770414815382 |

| | | | |
|---|---|---|---|
| C | -4.80672575051594 | 0.78324180722455 | -2.75073095686003 |
| C | -6.95462425098305 | 0.61391712298166 | -3.85941443174389 |
| C | -5.91030589745125 | 1.37417806852072 | -3.32225896305018 |
| C | -4.76521001513731 | -0.63150367309252 | -2.65587977009459 |
| C | -6.85559626384742 | -0.85751364014312 | -3.90697650868068 |
| C | -5.73963196768232 | -1.41204548612526 | -3.18656458243259 |
| C | -3.58952460617177 | 1.60274150366385 | -2.37418085671168 |
| C | -3.70367539499175 | 2.31316193190789 | -1.01811579317095 |
| C | -8.04165215074543 | 2.49895259242806 | -4.82389933476604 |
| C | -4.74784829208089 | 3.38930339460030 | -0.89738147052165 |
| H | -5.96247730107753 | 2.44965507586725 | -3.38602374135802 |
| H | -3.91418957113380 | -1.09903396932549 | -2.18510281986687 |
| H | -5.67897798654387 | -2.48792056674786 | -3.14281306449864 |
| H | -2.74330408350924 | 0.91029305767197 | -2.30857282377908 |
| H | -3.87238041184004 | 1.59887623889056 | -0.21310581931026 |
| H | -8.91857380638891 | 2.60964946480712 | -5.45629083543552 |
| H | -7.13243783026401 | 2.77995147305670 | -5.36044348347823 |
| H | -8.15291791440040 | 3.11693846438829 | -3.93172391038781 |
| H | -5.56465209843777 | 4.45952925754233 | 0.47835872779451 |
| H | -2.74797003600219 | 2.80384067943102 | -0.82016450177436 |
| O | -9.39509426368295 | 1.12844118059116 | -2.52797759655234 |
| H | -9.25150360606263 | -1.41950217168294 | -2.58775265038769 |
| H | -7.75235474476928 | 3.86982543019713 | -1.86071093208281 |
| H | -9.29529857846187 | 2.99888793596077 | -1.61832916301663 |
| C | -8.63024458004718 | 0.46504603605994 | -1.77965369889602 |
| C | -8.61987789646586 | -0.97732031251778 | -1.83090017964683 |
| C | -8.40393825864263 | 3.38322299379199 | -1.12850517225877 |
| H | -8.67593307323663 | 4.09717649070782 | -0.35223171335528 |
| C | -7.76602563420588 | 1.05418775427796 | -0.76304793068658 |
| C | -7.92307684769872 | -1.74377995205667 | -0.94768068802435 |
| O | -7.66289600199245 | 2.36184661681657 | -0.47349497616986 |
| H | -7.97332184673557 | -2.81889150721663 | -1.01861875271196 |
| C | -7.03764601937055 | 0.22988292657626 | 0.09055096928637 |
| C | -7.11602548267169 | -1.15088930020204 | 0.03684581823879 |
| H | -8.15429264097978 | -3.32478587601155 | 1.47364141219184 |
| H | -6.44453370179515 | 0.72586783705684 | 0.84568099271519 |
| O | -9.60345393623427 | -1.88074482970026 | 2.67308822392904 |
| C | -6.42522839444690 | -2.06255636744760 | 1.02964048901787 |
| O | -3.87407297362778 | -3.06186192266140 | -0.20279755065435 |
| H | -5.98716422509392 | -2.89476280723877 | 0.46768780439719 |
| C | -7.48750030232509 | -2.63795924613478 | 1.99058834949955 |
| H | -10.15504194002162 | -1.19689479786746 | 3.10068172855712 |
| H | -3.26522586097137 | -3.79385940690414 | -0.38683103838914 |
| C | -8.32160925237768 | -1.55496110845183 | 2.62227900074070 |
| H | -4.44499274604971 | -0.56263101535644 | 0.12744811210984 |
| H | -2.05430215853345 | -1.25789066654544 | -0.71054602195174 |
| C | -4.18909459618877 | -1.05333516762386 | 1.08050451212377 |
| O | -7.90214098240077 | -0.50232760205232 | 3.05119624602420 |
| C | -3.27066347283191 | -2.24258683769685 | 0.76906177037370 |
| N | -5.36046033810912 | -1.48367781126109 | 1.81995251618164 |
| H | -6.96522603861498 | -3.17032631687037 | 2.78659500059000 |
| C | -1.90181983768048 | -1.75682974554258 | 0.25691785343188 |
| H | -0.35312994117500 | -2.73049808595010 | -0.46675340086058 |
| O | -1.10495244048018 | -2.90834770761491 | 0.11321848794426 |
| H | -5.73219997747263 | -0.71014478600521 | 2.36481440490209 |
| H | -0.18905871643985 | 0.42056931968997 | -0.18603862986721 |
| H | -3.11043922474369 | -2.81395629887202 | 1.69541388193095 |
| O | -4.23172003588340 | 1.07963586325872 | 2.08889954202005 |
| C | -3.44216364796552 | -0.04685473840466 | 1.96821257240449 |
| O | -2.20349510106437 | 0.34137205207889 | 1.41900256534779 |
| C | -1.30387878510052 | -0.73947467241861 | 1.24235569867514 |
| C | 0.01362297184606 | -0.13996816111156 | 0.73491795589818 |
| H | 0.72662615985030 | -0.94043070896045 | 0.53454675756895 |

| | | | |
|---|---|---|---|
| H | -3.82705334365040 | 1.71459457578911 | 2.69876050412393 |
| H | -3.26380175892928 | -0.52778365836392 | 2.95136108572139 |
| H | -1.13114885411616 | -1.23886559466986 | 2.20897377820044 |
| O | 0.62907857196332 | 0.68061041704276 | 1.69269472300194 |
| H | 0.01991441092389 | 1.39646042361628 | 1.92115666853319 |

Transitional state XIII

| | | | |
|---|---|---|---|
| O | -2.19714130518866 | 3.72219272025207 | -7.08840465023823 |
| O | -2.22665646153454 | 0.04874632717159 | -5.31491379625659 |
| O | 0.02692716089098 | 0.87639629134779 | -7.09608592088163 |
| O | -3.80023116291681 | 4.48034046170398 | -5.64826819354541 |
| O | -0.04172642101560 | 4.44039114288391 | -9.00420540175697 |
| N | -3.08280562795052 | 2.48783249676737 | -3.75813333839074 |
| C | -1.74106896021980 | 1.37139358704105 | -5.49835268587363 |
| C | -1.22491775718545 | 1.52044674873714 | -6.94779761494191 |
| C | -2.90389978943114 | 2.33341415281768 | -5.20794852193415 |
| C | -0.99323021983538 | 2.99939849745770 | -7.29586080795034 |
| C | -2.61564964405214 | 3.75076073369911 | -5.73213560611319 |
| C | -0.59868010443478 | 3.16850995855130 | -8.77050059147611 |
| H | -0.91186254228354 | 1.57226650266467 | -4.79910681532565 |
| H | -1.97027160268049 | 1.08741938341615 | -7.63531944602060 |
| H | -3.81227795068956 | 1.95126843606851 | -5.69974077840967 |
| H | -0.19293106790943 | 3.40628480559388 | -6.65405709551477 |
| H | -1.81642786331578 | 4.21940713266102 | -5.11939971876961 |
| H | -1.48247731550388 | 2.99471349764138 | -9.40164772390227 |
| H | 0.16811402012235 | 2.42773606699207 | -9.01970906518259 |
| H | -3.58074644496430 | 3.36373970208084 | -3.59498924401114 |
| H | -1.50846875134797 | -0.58149607383021 | -5.43843252044930 |
| H | -0.06536940425477 | -0.07928825301916 | -7.05398311614067 |
| H | -3.66569728710964 | 5.35017908997581 | -6.04202990332652 |
| H | -0.69312968264732 | 5.12419458372000 | -8.81656640032566 |
| O | -8.07089419967105 | 3.20940728498439 | -4.89971198647325 |
| O | -9.19260824604813 | 0.76757191386256 | -4.62480461387476 |
| O | -4.06632928996544 | 3.36435952367578 | 0.02541872826263 |
| O | -5.19563058828807 | 3.66853718111092 | -1.86003831016845 |
| C | -5.20908525465556 | 1.17040590092228 | -3.68812075489008 |
| C | -7.27928852258284 | 2.17195993849702 | -4.53447224884141 |
| C | -5.97714660557965 | 2.28764449075115 | -4.17964193655846 |
| C | -5.73526449481681 | -0.06568313476309 | -3.69992121110343 |
| C | -7.99778747911001 | 0.88265525923454 | -4.45493214772972 |
| C | -7.08920410701374 | -0.33891380450766 | -4.27876248198915 |
| C | -3.81485563572097 | 1.39841166075516 | -3.11710347189178 |
| C | -3.83942040784221 | 1.67159784430272 | -1.60302664441148 |
| C | -7.55778789660614 | 4.52952369971638 | -4.80960365450629 |
| C | -4.44289487577911 | 2.99427966272571 | -1.20093186508148 |
| H | -5.49414943948548 | 3.25332674354293 | -4.21677360938384 |
| H | -5.19171437642005 | -0.92578716539147 | -3.32903617772634 |
| H | -6.93039736992626 | -0.65599245816546 | -5.32777992964008 |
| H | -3.23812490529319 | 0.47637180545342 | -3.25589777002687 |
| H | -4.40923751860779 | 0.89112782772901 | -1.08945299830058 |
| H | -8.37715956954959 | 5.19542652990300 | -5.07751947572673 |
| H | -6.72412355455493 | 4.67667980728985 | -5.50316948036863 |
| H | -7.22247515465573 | 4.74765854116468 | -3.79088629371304 |
| H | -4.46026581061083 | 4.21947508703616 | 0.24810664323532 |
| H | -2.82271280825868 | 1.63090125330975 | -1.20571920656200 |
| O | -7.76129624152367 | -1.42906628579529 | -3.66971814793476 |
| H | -7.53109528113251 | -3.73248197064758 | -2.61970101529912 |
| H | -7.78041302289333 | 2.68921179771161 | -1.52837379621075 |
| H | -8.53080298569553 | 1.76051704900180 | -0.19904027238494 |
| C | -7.62182860644138 | -1.62612547344057 | -2.31356136949727 |
| C | -7.49131533295840 | -2.93954565807726 | -1.88372254230941 |
| C | -7.71101561301803 | 1.78303122848837 | -0.92425999960421 |
| H | -6.75700680576356 | 1.78507303853050 | -0.39056358176829 |

| | | | |
|---|---|---|---|
| C | -7.60102495256120 | -0.58128357175532 | -1.37280546528279 |
| C | -7.26296219219841 | -3.22451970677995 | -0.54564179839620 |
| O | -7.79458876355158 | 0.69252094453545 | -1.82881349962155 |
| H | -7.12806728998740 | -4.25266962293122 | -0.23350233997388 |
| C | -7.35645220871363 | -0.87977507315711 | -0.03875418779789 |
| C | -7.15429469650848 | -2.19323749506047 | 0.38084091253061 |
| H | -8.55298735604232 | -3.56778157479595 | 2.35423720776744 |
| H | -7.30026934209979 | -0.07783645662511 | 0.68303429846233 |
| O | -9.85925569260242 | -1.92858661901501 | 3.76474349125890 |
| C | -6.73770945894234 | -2.52145222695892 | 1.80507617446009 |
| O | -3.86870018667312 | -3.56139749738322 | 1.60586432430354 |
| H | -6.17803697382063 | -3.46432481767257 | 1.77753222614672 |
| C | -7.92723068257877 | -2.76214356810119 | 2.74437927638377 |
| H | -10.41515567185605 | -1.17445641172345 | 3.99513731463611 |
| H | -3.26482068572072 | -4.21410208061201 | 1.97423729594738 |
| C | -8.81524681189880 | -1.57297220614243 | 3.01758736828454 |
| H | -4.78630643836100 | -1.39208428040752 | 0.55340083683244 |
| H | -2.18414322429086 | -2.01193799901877 | 0.39891344204736 |
| C | -4.62606570095264 | -1.28589765203089 | 1.63786291170773 |
| O | -8.63812832707115 | -0.43876808455949 | 2.65043978656968 |
| C | -3.52962978425972 | -2.26830329409087 | 2.08067744343839 |
| N | -5.86104305558837 | -1.51001522686745 | 2.40001979004266 |
| H | -7.54900008187188 | -3.09064730345992 | 3.71604961210283 |
| C | -2.16175059982913 | -1.85284929063329 | 1.48941839815610 |
| H | -1.11283077554380 | -3.50414126735769 | 1.75107672539521 |
| O | -1.11719207597367 | -2.60172728903519 | 2.08187852060310 |
| H | -6.36755333298289 | -0.62381941888256 | 2.44908792653742 |
| H | -0.79325058712266 | 0.35165939989927 | 0.06436573674408 |
| H | -3.46251419179081 | -2.27392700092718 | 3.18164722480696 |
| O | -5.12915185707506 | 1.02500807156406 | 1.38278560678105 |
| C | -4.18415987611344 | 0.15778006012193 | 1.92590047235637 |
| O | -2.93305241930535 | 0.42547749849872 | 1.30691560990508 |
| C | -1.86725404120531 | -0.37479687754680 | 1.79628194611448 |
| C | -0.57404329326781 | 0.09889907353933 | 1.11269981710898 |
| H | 0.15896948336862 | -0.71390490163347 | 1.13745558195519 |
| H | -4.75816915137582 | 1.92172227825263 | 1.38419252193148 |
| H | -4.08544995307879 | 0.31526249454425 | 3.02119042647976 |
| H | -1.76801923641145 | -0.24414901784650 | 2.88780034554354 |
| O | -0.00050674334467 | 1.18865189464212 | 1.79583176512795 |
| H | -0.57972112689844 | 1.95721503751255 | 1.75245254378634 |

The structure of the intermolecular ferulic acid complex with $NO_2^{\bullet}$

| | | | |
|---|---|---|---|
| O | -3.11953607361865 | 1.38980948301554 | 0.01392708074157 |
| O | -4.21226201317614 | -1.07312144075667 | 0.00341728732338 |
| O | 4.24468329443014 | 1.46652216239565 | 0.28756000063305 |
| O | 4.17997153981275 | -0.75533235071404 | 0.14660747705020 |
| C | -0.08207734483188 | -0.72650724393658 | 0.05967112744788 |
| C | -2.26824275415514 | 0.32238899746616 | 0.04465928170942 |
| C | -0.89184486551665 | 0.41536459241091 | 0.07526518004230 |
| C | -0.70003948399056 | -1.97854214959227 | 0.01146530996421 |
| C | -2.89015994670336 | -0.95579857900528 | 0.01500911246778 |
| C | -2.07100314119397 | -2.09005582510151 | 0.00852773605680 |
| C | 1.36353211367173 | -0.64747410035903 | 0.09265329425133 |
| C | 2.13001070258484 | 0.44421280098655 | 0.16934879052050 |
| C | -2.57471990105083 | 2.69100808227144 | 0.12027381962236 |
| C | 3.63375938701487 | 0.37149887527159 | 0.20324332501371 |
| H | -0.41536700973564 | 1.38128494170937 | 0.10892252172468 |
| H | -0.08689224316223 | -2.86702352888119 | -0.00394594793965 |
| H | -2.55669236791350 | -3.05358119774007 | -0.00987950381091 |
| H | 1.89367395343725 | -1.59355572244991 | 0.05496964832672 |
| H | 1.73823850116232 | 1.45095648625514 | 0.21868669125085 |
| H | -1.92170895713996 | 2.90924501129554 | -0.72954521644155 |
| H | -2.02211279190332 | 2.80701196006772 | 1.05648986924453 |

| | | | |
|---|---|---|---|
| H | -3.43652241467094 | 3.35439710519674 | 0.11309186953003 |
| N | -4.93211310060392 | -0.59580850201927 | 1.45909416395001 |
| O | -4.07780131996998 | -0.14834051297415 | 2.24368701084617 |
| O | -6.02717815153190 | -0.06656624704035 | 1.21965873732735 |

Structure of the intermolecular complex of the ferulic acid dimer (XIV) with $NO_2^{\bullet}$

| | | | |
|---|---|---|---|
| C | 2.96878524492477 | -0.88927278331074 | 2.32027872511507 |
| C | 1.76402874839009 | -0.29986010767264 | 1.98232509487744 |
| C | 0.68850171721281 | -0.41423744937966 | 2.85273415586590 |
| C | 3.12938452153595 | -1.62422834359793 | 3.49031322286137 |
| C | 0.83349960494394 | -1.11295263389947 | 4.02789438743049 |
| C | 2.04069480481717 | -1.73085110977687 | 4.38474849145276 |
| O | 4.38608349613592 | -2.11966609140212 | 3.67332647288651 |
| C | 4.66321621488717 | -3.03979021038663 | 4.71806742137304 |
| O | 2.11696779796953 | -2.39766063604422 | 5.54474156095086 |
| C | 1.63023122333663 | 0.39698079459849 | 0.65534892190813 |
| C | 1.35589593476554 | -0.59252917277408 | -0.50974022340252 |
| C | 0.95404409765794 | -2.02999859226983 | 0.00458761975111 |
| C | 0.20534837093662 | 0.05528918246037 | -1.20354973400515 |
| C | -0.22089235530723 | 1.12636203333831 | -0.42977676728804 |
| C | -1.33953292622132 | 1.87766542660981 | -0.78729571960421 |
| C | -0.45909293196658 | -0.29019867874885 | -2.35936693970053 |
| C | -2.00216342253958 | 1.51565679912269 | -1.95136062297416 |
| C | -1.57005857259994 | 0.45896847275119 | -2.75451342685008 |
| O | 0.55987211217449 | 1.35151565202578 | 0.66828340822430 |
| O | 1.82390722538886 | -2.90983391665984 | -0.13617062268884 |
| O | -0.18534706746005 | -2.17534897956770 | 0.47710733710812 |
| O | -1.69736681729863 | 2.91486243147818 | 0.03195066771200 |
| C | -2.80828501748932 | 3.70722645437380 | -0.34788784813904 |
| C | -2.25640672537733 | 0.10792590294444 | -3.99028791601309 |
| C | -3.11419983509368 | 0.84920892268292 | -4.68957901675139 |
| C | -3.58462403721945 | 2.26540788912880 | -4.39527844354384 |
| O | -2.81052870265013 | 3.20278043714940 | -4.68507960140733 |
| O | -4.74220002387007 | 2.38701733853834 | -3.93215834081064 |
| H | 3.81371372571158 | -0.83398422793295 | 1.65028952521544 |
| H | -0.25955786071247 | 0.02975608276326 | 2.59106934820049 |
| H | -0.00003149396499 | -1.24210219887361 | 4.70361523015563 |
| H | 5.70073162930211 | -3.32470082132812 | 4.55842673437119 |
| H | 4.01115257668837 | -3.91515682592175 | 4.65520920666506 |
| H | 4.53006431675406 | -2.57196090872213 | 5.69554101659383 |
| H | 2.55452740889776 | 0.95913410391004 | 0.45172279040047 |
| H | 2.23849440826138 | -0.72988170404228 | -1.13871177899539 |
| H | -0.12130606418581 | -1.13168120899530 | -2.94583234679188 |
| H | -2.88377456693584 | 2.05364331680630 | -2.25531122284184 |
| H | -2.85861936952256 | 4.49214669887424 | 0.40250182694085 |
| H | -3.73176138988409 | 3.12123926332779 | -0.34625672943739 |
| H | -2.66690653908916 | 4.13872092483298 | -1.34330454894420 |
| H | -2.00009778832044 | -0.87850952291203 | -4.36495463940080 |
| H | -3.55881878890425 | 0.44134357112360 | -5.58926854204898 |
| N | 1.29040531392459 | -3.88071668064518 | 5.49737912260131 |
| O | 0.06325499681695 | -3.68399092078922 | 5.53413002014623 |
| O | 1.85796595339922 | -4.64761176781370 | 4.69301026588253 |

Structure of the intermolecular complex of the ferulic acid (XIV) dimer with $NO_2^{\bullet}$

| | | | |
|---|---|---|---|
| C | 0.19216271515761 | -0.92778311872518 | 2.68539583696181 |
| C | 1.25231743842198 | -0.08085820513683 | 2.60341506597933 |
| C | 2.37959314919273 | -0.30292921906287 | 3.45652008371588 |
| C | 0.15286424804751 | -1.97634116875593 | 3.71189502559844 |
| C | 2.51939078397145 | -1.42202636384154 | 4.22931996183729 |
| C | 1.50140123864895 | -2.43399808859950 | 4.25312402721939 |
| C | 1.33896219683727 | 1.02842923496913 | 1.67487869176466 |
| C | 0.48691479909528 | 1.39970131876867 | 0.70634304684770 |

| | | | |
|---|---|---|---|
| C | 0.74557742029374 | 2.68179407687203 | -0.08833424306295 |
| O | -0.72072516865819 | 0.78404035210175 | 0.49114194506131 |
| C | -1.16582884980489 | 0.70023614894202 | -0.81296423516217 |
| C | -0.28385562358142 | 0.36300333251439 | -1.83443526734007 |
| C | -2.52543579935025 | 0.85263243674525 | -1.11231284827485 |
| C | -0.72320801380644 | 0.14306199800687 | -3.12096077224399 |
| C | -2.95492830277405 | 0.60472954390937 | -2.40899504015934 |
| C | -2.08037893890797 | 0.23119698347125 | -3.42706253799185 |
| C | -2.61203452514944 | -0.01647591272988 | -4.75980749558751 |
| C | -2.00800752157261 | -0.62295352967662 | -5.77995942087892 |
| C | -0.62174324411429 | -1.24522686065807 | -5.81726086275457 |
| O | 0.27184673977946 | -0.59158935178896 | -6.40357708309325 |
| O | -0.49362983461068 | -2.38606708980185 | -5.32198011745515 |
| O | -0.26309282397403 | 3.24853662607117 | -0.56122634812981 |
| O | 1.92436512505489 | 3.08016491031174 | -0.16192428266345 |
| O | 1.60900851882932 | -3.57563069792807 | 4.74922660406334 |
| O | 3.73674108281700 | -1.60253790832392 | 4.84731493097605 |
| O | -3.49926992144179 | 1.21963390729054 | -0.22240712486453 |
| C | -3.14364933453288 | 2.03008324015867 | 0.88708554436855 |
| C | 3.75401073184461 | -2.28180189116642 | 6.09641774841235 |
| H | -0.66287082864000 | -0.82526787857408 | 2.03968514956233 |
| H | 3.19624516888310 | 0.40205507071448 | 3.43278583268625 |
| H | -0.48373933396021 | -2.83378031122750 | 3.48029187220864 |
| H | 2.21656424457641 | 1.65683187693406 | 1.75072261756742 |
| H | 0.76759659411539 | 0.28293466949961 | -1.58896576647147 |
| H | -0.00787400682141 | -0.10307127962488 | -3.88991448477068 |
| H | -4.00926238680817 | 0.71852222368979 | -2.60842796159278 |
| H | -3.62846372737257 | 0.33610907494968 | -4.89850098513064 |
| H | -2.52898250297475 | -0.72244391158171 | -6.72441948495094 |
| H | -2.73227307371083 | 1.43068395324420 | 1.70397677142871 |
| H | -4.07139335780100 | 2.50460822049644 | 1.19988478201346 |
| H | -2.40868048125253 | 2.78679828962895 | 0.59957175965544 |
| H | 4.77176061684971 | -2.16538304605849 | 6.45976895128564 |
| H | 3.49911419877312 | -3.33651020162886 | 5.97622733349975 |
| H | 3.04263269693330 | -1.82508748401224 | 6.79073182910420 |
| N | -0.34081512056378 | -1.46664149691552 | 5.03364689988571 |
| O | -0.06956436191557 | -0.28882104388065 | 5.37116382394699 |
| O | -0.76426345699492 | -2.31629951523380 | 5.85685498863582 |

The structure of the intermolecular ferulic acid-glucosamine (XII) complex with $NO_2^{\bullet}$

| | | | |
|---|---|---|---|
| O | 2.33999527202169 | 0.15525298940590 | -5.55978570242024 |
| O | 3.27442532622808 | 0.36649861748955 | -1.48799147414050 |
| O | 4.98328736508511 | -1.00908957239757 | -3.35829267049004 |
| O | 0.62827855234416 | 1.58901630351833 | -5.33294668526782 |
| O | 4.01626276074320 | -1.25860007577572 | -7.37887030222617 |
| N | 1.43561718272992 | 2.27110190529828 | -2.66750659491869 |
| C | 3.23415090574937 | 0.62439600694723 | -2.87761693706481 |
| C | 3.66914217895843 | -0.62084576345837 | -3.67329685718103 |
| C | 1.82331353721033 | 1.03456900563967 | -3.32894167094244 |
| C | 3.62381560053009 | -0.30043542770569 | -5.16930589794177 |
| C | 1.88056773949262 | 1.27667384506824 | -4.84378879700779 |
| C | 3.93689849982382 | -1.54306755313549 | -6.01093323594154 |
| H | 3.90807190068000 | 1.46361826459630 | -3.10518904817264 |
| H | 2.94541579790976 | -1.41802447315987 | -3.45018574456843 |
| H | 1.12448779364869 | 0.20663969610955 | -3.12141663311515 |
| H | 4.36546535789263 | 0.48839021821327 | -5.37430090451994 |
| H | 2.58763922954177 | 2.11900592278626 | -5.00641478244885 |
| H | 3.16159966650616 | -2.29146278654214 | -5.80262530954415 |
| H | 4.91145995354297 | -1.92777284146691 | -5.70837761017445 |
| H | 0.84535962283341 | 2.83700406467907 | -3.27967366680549 |
| H | 4.21125616502852 | 0.35864649446133 | -1.22964847058405 |
| H | 4.98931547168816 | -1.47273292551361 | -2.50935420571326 |
| H | 0.70766951983871 | 1.79552563121192 | -6.28083783783042 |

| | | | |
|---|---|---|---|
| H | 3.17330615925185 | -0.87251934817168 | -7.65673404304248 |
| O | -3.36431869382984 | 0.54436608919827 | -3.84289207515131 |
| O | -4.06555417856679 | -0.91675271473412 | -1.71303121504091 |
| O | -1.86000377289830 | 4.45455607999085 | -1.17462672075899 |
| O | -0.56853704904284 | 4.32258860135391 | -2.97109180957925 |
| C | -0.58240265263847 | 1.27031153285899 | -1.53045432665607 |
| C | -2.55137539363584 | 0.58377705556263 | -2.74277578299325 |
| C | -1.36554791657114 | 1.26802373450495 | -2.68919246755980 |
| C | -1.03768033956893 | 0.57902213257517 | -0.42448873535011 |
| C | -3.04566365717266 | -0.17260421159646 | -1.60962453180661 |
| C | -2.24914566675135 | -0.09490484788263 | -0.41617146830473 |
| C | 0.68469362154954 | 2.09552649308262 | -1.43210249108950 |
| C | 0.31463972261988 | 3.48815416763991 | -0.89672565364158 |
| C | -2.97006332546032 | 1.28261764848764 | -4.98982728812276 |
| C | -0.80404018839620 | 4.13543096166877 | -1.75780403361938 |
| H | -1.01853466818387 | 1.82444491631933 | -3.54610149160331 |
| H | -0.45255325301556 | 0.59299378069777 | 0.49076712379514 |
| H | 1.35108051200125 | 1.59542143988423 | -0.71285344981292 |
| H | -0.05833084487086 | 3.40185042467149 | 0.12568428828302 |
| H | -3.76236076905618 | 1.10364541831037 | -5.71205960935867 |
| H | -2.00698677147352 | 0.92564786593472 | -5.36405732083229 |
| H | -2.88946971912438 | 2.34841310947722 | -4.75527862873132 |
| H | 1.20015435605309 | 4.12485829350268 | -0.92765261263925 |
| O | -4.66712977195129 | 0.56532674067086 | 0.91970996852977 |
| H | -6.58419854087295 | 0.54906674201704 | 2.27923998566169 |
| H | -6.72679251502498 | -1.05432809579413 | 1.54695329370623 |
| C | -3.95619865223012 | -0.37989790901954 | 1.38410953878621 |
| C | -2.69252232100168 | -0.74910295749472 | 0.80489674705671 |
| C | -6.64537956115105 | -0.51899596193598 | 2.49639169529842 |
| H | -7.49016609593667 | -0.74366415739482 | 3.14461281765292 |
| C | -4.29535856698895 | -1.08618736579004 | 2.59545642075441 |
| C | -1.83853597197819 | -1.62446927828529 | 1.45548514861088 |
| O | -5.51330149213483 | -0.96001229162961 | 3.23076418519689 |
| H | -0.92519744050637 | -1.89371959209271 | 0.93698497660432 |
| C | -3.38242251390353 | -1.89242589062839 | 3.23831442487232 |
| C | -2.12605641096124 | -2.17050819116367 | 2.69803994867027 |
| H | -2.26917888794141 | -4.66877746669782 | 3.93036265338394 |
| H | -3.68124721854600 | -2.31804580291692 | 4.18513545306217 |
| O | -3.32528048591234 | -4.08691703482499 | 6.06304230007474 |
| C | -1.05461170501009 | -2.95829269079487 | 3.42174608900671 |
| O | 1.94942708394819 | -2.89803274013642 | 2.12797216191694 |
| H | -0.46939368807672 | -3.49957638486162 | 2.66301430305692 |
| C | -1.59328212096222 | -3.97391271181924 | 4.43227063892170 |
| H | 1.54765818281537 | -2.64511791820663 | 1.26883135939848 |
| C | -2.35106369917861 | -3.40895781263015 | 5.66864734298117 |
| H | 0.13317412743709 | -0.84511748207297 | 2.40768985260328 |
| H | 2.42741705289165 | -0.43404086768292 | 1.31425658724534 |
| C | 0.65543083815506 | -1.14943677998477 | 3.32850354536517 |
| O | -1.93334343395365 | -2.37386658758003 | 6.22812315035254 |
| C | 2.01808869808807 | -1.75894528735343 | 2.95278255890454 |
| N | -0.10344660972134 | -2.09250331401722 | 4.12885932268634 |
| H | -0.74154735075851 | -4.52998555557670 | 4.83021574801262 |
| C | 2.90195646587398 | -0.70022935192443 | 2.27695817150558 |
| H | 4.09781330109350 | -2.14935504294631 | 1.84311446500293 |
| O | 4.19373489804299 | -1.20749112628798 | 2.06623400202102 |
| H | -0.63435458136162 | -1.61739965465201 | 4.85916883545032 |
| H | 3.23061938317786 | 1.91461086489162 | 1.51844735404858 |
| H | 2.49686429321178 | -2.11412366716837 | 3.87407657938173 |
| O | -0.32809510482270 | 0.73619378105423 | 4.37757612285306 |
| C | 0.88982332735657 | 0.13042135607822 | 4.13973735974376 |
| O | 1.71051954665953 | 1.05655621341638 | 3.46486546127996 |
| C | 2.99900973692532 | 0.54930692793031 | 3.15289672958226 |
| C | 3.77520054976515 | 1.65964395279687 | 2.43746815150379 |

| | | | |
|---|---|---|---|
| H | 4.76174467767525 | 1.27175075199640 | 2.18232986331134 |
| H | -0.19076615335176 | 1.56346474152887 | 4.86927154704107 |
| H | 1.38547010834445 | -0.15377423839738 | 5.09336169790765 |
| H | 3.51121815454880 | 0.27834682012391 | 4.09031564121870 |
| O | 3.97542475414506 | 2.78539578574712 | 3.24502067267172 |
| H | 3.10963709917611 | 3.13227760297783 | 3.50124358313924 |
| N | 1.10036941878954 | -2.44184112165256 | -1.40234824575621 |
| O | 0.53443044175461 | -1.87689974381711 | -2.37963888393317 |
| O | 0.91272630780957 | -1.92279772486546 | -0.25473785020325 |

Structure of the intermolecular ferulic acid-glucosamine complex (XIII)c $NO_2^{\bullet}$

| | | | |
|---|---|---|---|
| O | 1.46379214129806 | 4.12698752668761 | -5.41295964635450 |
| O | 2.56900627647442 | 1.30888425450432 | -2.54713789092124 |
| O | 3.98903944463330 | 1.50970059655229 | -4.90417767561332 |
| O | -0.11624692326237 | 4.97549777647823 | -4.05065286199806 |
| O | 2.94808193940340 | 4.45416233646812 | -7.81400990618931 |
| N | 1.01625699767469 | 3.69206411200758 | -1.78357513268001 |
| C | 2.53009861867019 | 2.49293329260993 | -3.30359536925132 |
| C | 2.75116055713559 | 2.17116130097344 | -4.78929653193831 |
| C | 1.18437259557075 | 3.21155693248141 | -3.14818116347198 |
| C | 2.71757477282654 | 3.48280583510484 | -5.58725022425607 |
| C | 1.15419802750152 | 4.43454162176924 | -4.07453081146202 |
| C | 2.89460495656347 | 3.25376788711158 | -7.09333520635708 |
| H | 3.32569654036597 | 3.17100324355229 | -2.95314757339835 |
| H | 1.91412645981661 | 1.53497779998923 | -5.10718376955762 |
| H | 0.38191196542336 | 2.52395469160530 | -3.46367960393628 |
| H | 3.51714870553536 | 4.14296442187639 | -5.21387639355784 |
| H | 1.90294466527426 | 5.16218692394113 | -3.69248805305498 |
| H | 2.06733730657303 | 2.62224537897172 | -7.44194895503587 |
| H | 3.84932549632157 | 2.75676957088422 | -7.26866764371579 |
| H | 0.33352644382011 | 4.45090394600917 | -1.77477583077972 |
| H | 3.37579799776225 | 0.83544918259681 | -2.81871700823034 |
| H | 3.97606722682980 | 0.90010491716680 | -5.65495299979920 |
| H | -0.16196521112501 | 5.73019813736635 | -4.66187936028138 |
| H | 2.14154646697348 | 4.95458198279520 | -7.62888897825306 |
| O | -4.17865897908971 | 2.40332630996151 | -2.19532785165439 |
| O | -4.03089128558484 | -0.30936533509064 | -2.03793033908767 |
| O | -2.13625662770812 | 3.90704341474083 | 0.94109291231381 |
| O | -0.97270171190900 | 5.30731834393320 | -0.32305600626927 |
| C | -0.60062826451956 | 1.83804018618261 | -1.37957977570188 |
| C | -2.95540739893154 | 1.81984378081193 | -2.00977782040716 |
| C | -1.79395363404542 | 2.49904811241297 | -1.80237979330737 |
| C | -0.52418454913148 | 0.49660242051772 | -1.43300626910775 |
| C | -2.99859113647887 | 0.37918443357284 | -1.96108019093064 |
| C | -1.62875202459821 | -0.28893081902505 | -2.00923109554923 |
| C | 0.54466221381069 | 2.67636059036563 | -0.84828059996533 |
| C | 0.15308233279826 | 3.34648645864160 | 0.47850892696782 |
| C | -4.28300358621593 | 3.80125660296104 | -1.97046829969841 |
| C | -1.09129343330745 | 4.26041121661846 | 0.35063448008530 |
| H | -1.77891296399913 | 3.57504938085312 | -1.87107484110185 |
| H | 0.34331064197306 | -0.05052086168580 | -1.09877686337442 |
| H | 1.38737439983651 | 1.99966466560225 | -0.65951368142130 |
| H | -0.06831852160791 | 2.57708347272584 | 1.21936692494000 |
| H | -3.85924957641937 | 4.07350421293113 | -0.99841618706013 |
| H | -5.34998692162852 | 4.00688138354032 | -1.99251512161971 |
| H | -3.76597066551461 | 4.35883691454233 | -2.75644558878179 |
| H | 0.98907545340716 | 3.96413345245632 | 0.81000003643698 |
| O | -1.63392255902698 | -1.65259809085097 | -1.70572418607007 |
| H | -1.39021905480424 | -4.03486712243314 | -1.18848851773007 |
| H | -3.35851484735604 | 0.44408602268544 | 2.28097383729119 |
| H | -1.60062383775635 | 0.69917609932194 | 2.44476788373451 |
| C | -1.74101155792753 | -2.10728813633998 | -0.41006391030196 |
| C | -1.55360972801287 | -3.47953889868864 | -0.27928391892209 |

| | | | |
|---|---|---|---|
| C | -2.45195067961709 | 0.76850494136756 | 1.76001528033882 |
| H | -2.56013715826673 | 1.80164494460541 | 1.42433147918581 |
| C | -1.98554260340395 | -1.34264460735356 | 0.74080216787948 |
| C | -1.55916418865041 | -4.09469302114978 | 0.95677326557924 |
| O | -2.22950221975645 | 0.00669023938974 | 0.59596860131017 |
| H | -1.38550443778082 | -5.15572269785432 | 1.03472765899077 |
| C | -1.95200793687766 | -1.97581343767464 | 1.97557322670155 |
| C | -1.74011013631463 | -3.34046097959730 | 2.10715650820226 |
| H | -3.70211403985224 | -4.17462885926006 | 3.87473197740454 |
| H | -2.09254626874014 | -1.38398917920439 | 2.86394404275528 |
| O | -4.29326760860583 | -1.86357493874009 | 4.39354876931436 |
| C | -1.58801968787689 | -3.96390861330528 | 3.47987367777191 |
| O | 0.75244645640687 | -5.76135262314481 | 2.75293330092532 |
| H | -1.44411950974354 | -5.04301230033220 | 3.34141306256937 |
| C | -2.82377112028457 | -3.75333885119875 | 4.36571402334370 |
| H | 1.25862482434794 | -6.59225622773290 | 2.75878721696167 |
| C | -3.15407521118454 | -2.27510851142216 | 4.70295873440043 |
| H | 0.63316832684946 | -3.16850126565128 | 2.35205106937914 |
| H | 2.79255461091526 | -4.42817781501881 | 1.77569047623938 |
| C | 0.81609225556000 | -3.44164338123520 | 3.40529675133795 |
| O | -2.28384751447169 | -1.59613141692045 | 5.29737749216565 |
| C | 1.51246780663698 | -4.80925020898390 | 3.44944063589654 |
| N | -0.40416593111192 | -3.47999797616600 | 4.19447614359923 |
| H | -2.65047122592415 | -4.25828720826143 | 5.31782983288166 |
| C | 2.91673469040108 | -4.70944076212766 | 2.83011632456692 |
| H | 4.20200015241209 | -6.09341601881858 | 2.28746528467595 |
| O | 3.51878410943549 | -5.97361215096592 | 2.96177765231058 |
| H | -0.62487655841878 | -2.54600581316582 | 4.54087383322944 |
| H | 4.97939867021500 | -3.11087022356391 | 1.88471044378536 |
| H | 1.60729762287516 | -5.10658619085878 | 4.50526640587685 |
| O | 1.16867349378876 | -1.12806616649783 | 3.75766639828227 |
| C | 1.73551431001591 | -2.36750348800670 | 3.99875068015091 |
| O | 3.02071285426572 | -2.36500684355510 | 3.42492574147054 |
| C | 3.70810102648471 | -3.60413728627767 | 3.54558616233314 |
| C | 5.10172519886977 | -3.39502667932208 | 2.93761558844484 |
| H | 5.67255115048046 | -4.32091024653372 | 3.01646630589962 |
| H | 1.75525650501412 | -0.43482797651146 | 4.09880200212323 |
| H | 1.82797381529872 | -2.55740627340252 | 5.08929131090362 |
| H | 3.80072816896210 | -3.86335471769542 | 4.61197193071953 |
| O | 5.84771962904262 | -2.42942203524076 | 3.62904833114870 |
| H | 5.35291051483373 | -1.59894426366589 | 3.62236766659259 |
| N | -1.55372580844023 | -0.27214627287935 | -3.51920897145469 |
| O | -1.03437659268202 | 0.73675518225931 | -4.07699766090846 |
| O | -2.25678249487933 | -1.09395142624740 | -4.16454875606074 |

**S2 Cartesian coordinates of the structure of the molecular complex of honey with glucosamine and ferulic acid**

| | | | |
|---|---|---|---|
| O | -0.67957824419494 | 0.56932184652353 | -1.14665052972285 |
| O | -0.88276950533837 | -1.37904586941938 | 2.52693662069170 |
| O | 1.50912210792524 | -1.48452378034461 | 0.90367613232510 |
| O | -2.62619405106504 | 1.65238962971504 | -0.75744382183321 |
| O | 1.73307313518800 | 0.42052867226339 | -2.75224824260960 |
| N | -2.66033609873925 | 0.88369371112711 | 1.96668278367424 |
| C | -0.58263417471903 | -0.42328483059099 | 1.53725001543423 |
| C | 0.27418706122606 | -1.02793276987334 | 0.41522536575609 |
| C | -1.85625217745959 | 0.21177952098129 | 0.95606539556447 |
| C | 0.53765106896518 | 0.03864121805090 | -0.65550420298137 |
| C | -1.47370549875398 | 1.19528309927948 | -0.16008333818345 |
| C | 1.29945274894766 | -0.55636822386759 | -1.84459342662505 |
| H | 0.01180824069415 | 0.34121656155278 | 2.05451900338888 |
| H | -0.28345001582985 | -1.85721802655908 | -0.05205957821951 |
| H | -2.48029396639364 | -0.57078585697180 | 0.50343739988138 |
| H | 1.14145040541957 | 0.84497492624129 | -0.20935465201449 |

| | | | |
|---|---|---|---|
| H | -0.88610803169654 | 2.03133148835884 | 0.28306487035036 |
| H | 0.65329771869521 | -1.29188576682728 | -2.33858627946623 |
| H | 2.19392480285767 | -1.05244082119832 | -1.46522450606927 |
| H | -2.21037665237965 | 1.74155136341356 | 2.26928377211798 |
| H | -2.74857502697709 | 0.27179987024346 | 2.77327424048723 |
| H | -1.46740990861784 | -2.05725549977540 | 2.15636374318633 |
| H | 1.35526284215825 | -1.95596760862829 | 1.73847957968496 |
| H | -2.41387831434233 | 2.19770510737789 | -1.53006905907196 |
| H | 0.95631405571373 | 0.86416199176427 | -3.12190825946823 |
| O | -11.31963187650804 | 1.10660904409586 | -2.49130702736091 |
| O | -12.01026559227937 | -1.35178567328363 | -3.87085232904239 |
| O | -5.09638334452541 | 1.13305470532018 | 0.89030845676579 |
| O | -4.70562041214653 | -0.97187182254151 | 0.24348445366826 |
| C | -8.46404173490810 | -0.98714058605901 | -1.80796450064260 |
| C | -10.56527590148668 | 0.00279942896859 | -2.53574088335319 |
| C | -9.36136241877261 | 0.07836233840876 | -1.85452590046815 |
| C | -8.81034037135470 | -2.18760328180982 | -2.48805368525641 |
| C | -10.93852639185535 | -1.22953903498637 | -3.24290765778411 |
| C | -9.97839631811502 | -2.30226850069520 | -3.16570861159677 |
| C | -7.21313620528407 | -0.92949131559470 | -1.10673575947560 |
| C | -6.71541718118562 | 0.11528742215246 | -0.41903821656688 |
| C | -12.58087820546053 | 1.20032904795254 | -3.13012762993345 |
| C | -5.41131804232297 | 0.02813239335605 | 0.25714074295007 |
| H | -9.14227196648094 | 1.00706339401231 | -1.35320092429516 |
| H | -8.11990680370776 | -3.01658796994391 | -2.45605943027036 |
| H | -10.24650149478780 | -3.21041876967935 | -3.68347615138272 |
| H | -6.60500011838671 | -1.82594698876514 | -1.13529885969303 |
| H | -7.21778666589091 | 1.06275480923111 | -0.31584797156945 |
| H | -13.27858720257957 | 0.45737523480212 | -2.73977075573600 |
| H | -12.49089942835762 | 1.05488010518872 | -4.20803973004400 |
| H | -12.93052191721117 | 2.20639273655378 | -2.90824377099675 |
| H | -4.16828429791576 | 1.09347173469578 | 1.32287686111019 |

Transition state IV:

| | | | |
|---|---|---|---|
| O | -2.35410765427096 | 0.15782714738650 | -2.09615317345674 |
| O | -2.92625644067166 | -0.53594174751267 | 1.91322520253330 |
| O | -0.33101262685917 | -0.77519544458952 | 0.82349965945434 |
| O | -4.39218811159585 | 1.05228826376693 | -2.38317013573944 |
| O | 0.11883184177118 | -0.06124361654614 | -3.42042371069067 |
| N | -4.73453131345677 | 1.22058945182961 | 0.51631737615023 |
| C | -2.51233014627815 | 0.12269433330074 | 0.74003783984101 |
| C | -1.45549786878353 | -0.69188919101921 | -0.01888655947487 |
| C | -3.76427499654198 | 0.36121548354632 | -0.12628209482447 |
| C | -1.15652322928113 | 0.01946717517329 | -1.34991785796555 |
| C | -3.34572479717841 | 0.95302447338395 | -1.48541925459316 |
| C | -0.18692339958204 | -0.76256274416349 | -2.24433332157677 |
| H | -2.05897378574564 | 1.09500332213648 | 1.00014213595911 |
| H | -1.86872037614397 | -1.68796101713513 | -0.23006653453802 |
| H | -4.24459390781927 | -0.61048696163580 | -0.29653574766391 |
| H | -0.73399246503415 | 1.01397509943138 | -1.13696042985933 |
| H | -2.96978348422352 | 1.97948980388247 | -1.33452031243882 |
| H | -0.63404579532113 | -1.73688801180817 | -2.48056367501484 |
| H | 0.76328358539995 | -0.90456455587434 | -1.72845115134883 |
| H | -4.36165506752485 | 2.15959410053857 | 0.60956550095515 |
| H | -4.90366646906870 | 0.86059768361084 | 1.45049932364790 |
| H | -2.13622071649085 | -0.76558537108098 | 2.42524173477513 |
| H | 0.27470635169662 | -1.46296369571688 | 0.51700583799522 |
| H | -4.58368807388581 | 0.16429356221087 | -2.71888639046389 |
| H | -0.71063373818617 | 0.15607426270261 | -3.87049355077875 |
| O | -7.75790496620262 | 2.06371421607527 | -1.99435064709029 |
| O | -7.76364953417716 | 0.55443955090120 | -4.47127445174832 |
| O | -6.20668588001232 | -1.53233929917016 | 3.87396046217653 |

| | | | |
|---|---|---|---|
| O | -5.80731609252875 | -3.38284745638761 | 2.68825513627299 |
| C | -6.95835135338842 | -1.25950501396802 | -0.86459079476374 |
| C | -7.50726433001484 | 0.76095069912877 | -2.10328320898837 |
| C | -7.26854203107533 | 0.09680885459512 | -0.90907851928464 |
| C | -6.92952773875479 | -1.98761476245691 | -2.08809986845064 |
| C | -7.50401973024596 | 0.02103355824327 | -3.37417859185707 |
| C | -7.19468091650363 | -1.38442180755610 | -3.27204811739544 |
| C | -6.61834203424352 | -1.94324854669246 | 0.34933500130978 |
| C | -6.62645756792557 | -1.42880755900346 | 1.59514920667367 |
| C | -7.86742166201995 | 2.91508951805590 | -3.11977802206677 |
| C | -6.17879647031638 | -2.22168711945032 | 2.73602853480542 |
| H | -7.27466339035379 | 0.69285392955876 | -0.01334111351977 |
| H | -6.69497942587240 | -3.04112974655035 | -2.05776074400511 |
| H | -7.18226201283798 | -1.93210338158347 | -4.20208502784482 |
| H | -6.30420272172612 | -2.97615369813554 | 0.25162344436684 |
| H | -6.93552438750371 | -0.42241822077121 | 1.82080543140652 |
| H | -8.72423377193791 | 2.64629857061312 | -3.74069031101297 |
| H | -6.96549474202850 | 2.86584020281498 | -3.73282511499740 |
| H | -7.99396814439309 | 3.91291321753893 | -2.70539107642269 |
| H | -5.89278131608751 | -2.05908454692694 | 4.63275126142557 |

Transition state V:

| | | | |
|---|---|---|---|
| O | -1.44938246207031 | -0.87525222103928 | -2.23903128353581 |
| O | -3.05399563432724 | 0.65114251165483 | 1.24050635098092 |
| O | -0.23316584314388 | 0.56725443058331 | 0.92125879413707 |
| O | -3.48963477286322 | -0.91410814411791 | -3.20791859343441 |
| O | 1.35811463256212 | -1.11769124019716 | -2.70270637431405 |
| N | -4.63077595551291 | 0.62976076921877 | -0.97234367430908 |
| C | -2.40053126758828 | 0.59502795367790 | 0.00018816402671 |
| C | -1.06711770065969 | -0.16707350258632 | 0.06071120721000 |
| C | -3.34952071855904 | -0.10339975797953 | -0.97823845767642 |
| C | -0.50379276311605 | -0.26637291556490 | -1.37252201938479 |
| C | -2.67839760669277 | -0.19342244955095 | -2.36205204768400 |
| C | 0.76482466576592 | -1.12831162180139 | -1.43182191009631 |
| H | -2.17646691718697 | 1.61576893924353 | -0.35152008485482 |
| H | -1.25273028672855 | -1.17750976730832 | 0.44788308043728 |
| H | -3.53591258223155 | -1.12305714869564 | -0.62810823269560 |
| H | -0.26905318504827 | 0.74682185665330 | -1.73395631415790 |
| H | -2.48667400468495 | 0.83019993416133 | -2.74913765497340 |
| H | 0.51042437467226 | -2.15078909797934 | -1.12720349459243 |
| H | 1.51503719819409 | -0.71660766959229 | -0.75558141057501 |
| H | -4.52595165544201 | 1.53799414967436 | -1.44255802476840 |
| H | -4.83297407703838 | 0.81391894518179 | 0.02347895956112 |
| H | -2.42796762186021 | 0.95078439239240 | 1.91492473309789 |
| H | 0.50386511277256 | 0.02653932676102 | 1.23187376527345 |
| H | -3.02520840535475 | -1.13742376329067 | -4.02815749239536 |
| H | 0.74041337344282 | -1.50642313928188 | -3.33740358164270 |
| O | -9.81211324615546 | 1.43166996682124 | -1.17507214153723 |
| O | -8.12263770436325 | 2.28234371962680 | -3.41358413857675 |
| O | -7.20185811357816 | -3.31140070086276 | 3.35807078787681 |
| O | -5.28446411389889 | -3.38505027716133 | 2.19821782026263 |
| C | -6.80835081371964 | -0.47889384330034 | -0.51942647907816 |
| C | -8.55300374818727 | 0.99455617499463 | -1.42596845414811 |
| C | -8.08008283032585 | 0.08903544634699 | -0.49600650827365 |
| C | -5.86854948331461 | -0.05587352063387 | -1.60757581310325 |
| C | -7.71849816571323 | 1.43615993640811 | -2.55452992738981 |
| C | -6.44927122928437 | 0.87745126357536 | -2.59350179645925 |
| C | -6.36146305267917 | -1.43044123233844 | 0.40918017756579 |
| C | -7.02584694458465 | -1.92643772510024 | 1.50062941427020 |
| C | -10.52925924630019 | 2.22441350072746 | -2.10281080388452 |
| C | -6.41287302872032 | -2.91097847068894 | 2.33734513590560 |
| H | -8.77117532089163 | -0.19660931518101 | 0.28206718363495 |
| H | -5.46277069618422 | -0.93134587934905 | -2.12674453698908 |

| | | | |
|---|---|---|---|
| H | -5.81940249288154 | 1.16458084149000 | -3.42221375234352 |
| H | -5.37373704431030 | -1.84930924493830 | 0.26463882382657 |
| H | -8.01852333215326 | -1.61914990169984 | 1.78139592561455 |
| H | -10.65626918112984 | 1.70019959727480 | -3.05191220273258 |
| H | -10.02165880840806 | 3.16977508625331 | -2.29803299914067 |
| H | -11.49578293037526 | 2.39709360078215 | -1.63265741478233 |
| H | -6.75017805355438 | -3.98036046681599 | 3.90213676358661 |

Transitional state VI:

| | | | |
|---|---|---|---|
| O | -0.95568343781475 | 0.47708650704977 | -1.59996942293783 |
| O | -1.17666346427200 | 2.05036510641461 | 2.19190789022479 |
| O | 0.21648351619728 | 3.46878006319053 | 0.18343828100354 |
| O | -2.68799026773503 | -0.94221233909892 | -1.27251312038925 |
| O | 0.53572905049090 | 1.62950893639911 | -3.71423218610993 |
| N | -3.21275647251200 | 0.21715271082969 | 1.30083650432871 |
| C | -1.42626510685657 | 1.89948948889811 | 0.81662133849093 |
| C | -0.15148657352893 | 2.12446978813092 | -0.01044628952208 |
| C | -1.98384402345382 | 0.48215686390639 | 0.58606139194077 |
| C | -0.43809684422503 | 1.79157923202816 | -1.48654910136543 |
| C | -2.18506930259079 | 0.28780495615020 | -0.92592949009631 |
| C | 0.82427417299018 | 1.83186273381342 | -2.35640190632140 |
| H | -2.17643671001012 | 2.64003511407307 | 0.48911732703462 |
| H | 0.62639495080803 | 1.44324054944348 | 0.36241544968407 |
| H | -1.23409717712847 | -0.23751452450084 | 0.93616058589738 |
| H | -1.17389497292936 | 2.51339231748204 | -1.87734135189447 |
| H | -2.91372088283067 | 1.02911726375742 | -1.29357296621130 |
| H | 1.52344843388242 | 1.06792891493802 | -1.99186129537387 |
| H | 1.28724456115654 | 2.81775030245679 | -2.29723676916740 |
| H | -3.94852008799523 | 0.83110954706720 | 0.96528130882875 |
| H | -3.06248387953436 | 0.42292190532617 | 2.28308748075725 |
| H | -0.74527968837042 | 2.90636564610532 | 2.33355912254674 |
| H | 1.11939293681366 | 3.62380065148281 | -0.12419155579293 |
| H | -2.08915193625183 | -1.63998249135096 | -0.96140785017372 |
| H | 0.03783399696744 | 0.80390888234346 | -3.80338782901162 |
| O | -5.48837389286570 | 2.12943169133320 | -1.04851099270349 |
| O | -4.67673466043176 | 1.00089997528201 | -3.58850572388652 |
| O | -8.51215081770518 | -2.33313580022880 | 3.52836618898108 |
| O | -8.29132738074119 | -4.02908079835642 | 2.09149308744098 |
| C | -6.43189288789477 | -1.33680104573622 | -0.69249394039260 |
| C | -5.65007180468516 | 0.84694419529579 | -1.41718313634689 |
| C | -6.22748779013349 | 0.02478237830132 | -0.46843475916876 |
| C | -6.01885847855184 | -1.88347572685720 | -1.93868867866257 |
| C | -5.21735964122319 | 0.30017561987313 | -2.71019951284748 |
| C | -5.44689560681696 | -1.11107736884980 | -2.89274705203012 |
| C | -7.04734700974839 | -2.20083871756840 | 0.27049484141174 |
| C | -7.51486945432083 | -1.85617854709177 | 1.48785941303607 |
| C | -5.06458262393460 | 3.13523406122020 | -1.95283098728137 |
| C | -8.12980900219111 | -2.85186101023148 | 2.36448357359729 |
| H | -6.52145245226605 | 0.47889072189668 | 0.46379122103721 |
| H | -6.16819830229727 | -2.93759573942042 | -2.11445471855481 |
| H | -5.12746383956205 | -1.51896762426750 | -3.83889540967510 |
| H | -7.15255513575701 | -3.24116859227972 | -0.01342497449951 |
| H | -7.46897390496733 | -0.85812513999168 | 1.89044253409327 |
| H | -5.71710312530439 | 3.18339417069978 | -2.82575992882255 |
| H | -4.04377748964084 | 2.95894391844604 | -2.29997258372379 |
| H | -5.11769794377209 | 4.06177632667662 | -1.38461738322101 |
| H | -8.91969440535028 | -2.99820592428791 | 4.11489147308477 |

Transitional state VII

| | | | |
|---|---|---|---|
| O | -0.63153817259514 | 0.24727717926514 | -2.68594441155754 |
| O | -3.22975039660443 | -1.22814483431769 | 0.17660177829854 |
| O | -0.48301825814147 | -1.34115073963593 | 0.62261535250702 |
| O | -2.34383518291334 | 1.22745412534395 | -3.79949110547571 |

| | | | |
|---|---|---|---|
| O | 2.20096760674285 | 0.11712733158703 | -2.39421881598562 |
| N | -4.14505960305998 | 0.85144877602301 | -1.58991994792692 |
| C | -2.33631469243336 | -0.34706658906333 | -0.44745630548232 |
| C | -0.97511572469880 | -1.03456567586656 | -0.66019634501284 |
| C | -2.91774219306191 | 0.11234415824784 | -1.78769674637369 |
| C | -0.03736270816981 | -0.10321838199788 | -1.44636367194697 |
| C | -1.85072062112498 | 0.93883313067381 | -2.54253019535225 |
| C | 1.30830942180093 | -0.76554694196504 | -1.76751460301255 |
| H | -2.17410802995790 | 0.53687770850494 | 0.19252331201922 |
| H | -1.14267360132139 | -1.94542152995287 | -1.25104346642166 |
| H | -3.15197749714385 | -0.76659234152478 | -2.39737952925670 |
| H | 0.13797249020197 | 0.80954741556204 | -0.85382143206043 |
| H | -1.65717718139151 | 1.87392043264832 | -1.96661768668008 |
| H | 1.12551769704689 | -1.64544876247975 | -2.39690808407859 |
| H | 1.79582670031524 | -1.06575421672329 | -0.83913591174179 |
| H | -2.78396713874892 | -1.58124728777491 | 0.96332983745869 |
| H | 0.19369426431640 | -2.02909860294609 | 0.57359605838324 |
| H | -1.64202241189558 | 1.59318007386590 | -4.35943951460448 |
| H | 1.80330192804469 | 0.42130392617111 | -3.22194223894751 |
| O | -9.06437796917041 | -0.35364829933067 | -3.32134260334359 |
| O | -6.69981215635369 | -1.54919060529382 | -3.58871324097860 |
| O | -7.36185482057968 | 0.37670343835513 | 4.88668094499678 |
| O | -9.25443087711403 | 1.10876316263102 | 3.95199494867451 |
| C | -7.84471096379799 | -0.30330101628882 | 0.15113197704072 |
| C | -8.33123549912779 | -0.50848798224808 | -2.21689189238388 |
| C | -8.68777949813628 | -0.09749559767498 | -0.95210907084869 |
| C | -6.60267367850240 | -0.96069461908621 | -0.05120620780059 |
| C | -7.03881765468422 | -1.18711717955154 | -2.44941606054661 |
| C | -6.22214917967495 | -1.38708923968390 | -1.27961135942235 |
| C | -8.27437779730850 | 0.15474212694426 | 1.43646521178368 |
| C | -7.59732897894896 | 0.05333548301971 | 2.60057396801844 |
| C | -10.33045235419613 | 0.28301315571129 | -3.24157927178323 |
| C | -8.16708369956005 | 0.56515979674929 | 3.84284138738459 |
| H | -9.63153286089979 | 0.39909545847317 | -0.78686324743354 |
| H | -5.93605383099890 | -1.11748515530464 | 0.77865118841217 |
| H | -9.24570666224796 | 0.63378351942116 | 1.48357715793968 |
| H | -6.62264948795926 | -0.39626447784423 | 2.68974483493232 |
| H | -11.00448025952231 | -0.27512675940684 | -2.58855436270323 |
| H | -10.71462969252799 | 0.28320456134760 | -4.25861686352768 |
| H | -10.22642859156587 | 1.30863337588217 | -2.88176076522329 |
| H | -7.74648372915503 | 0.71679817460648 | 5.71615905500147 |
| H | -4.54723385070701 | 1.07627473645986 | -2.49261596793161 |
| H | -3.95673696592488 | 1.71889934919029 | -1.09876364846094 |
| H | -5.27333314767346 | -1.87717850405865 | -1.41990517293137 |

Transitional state VIII

| | | | |
|---|---|---|---|
| O | -0.25278593621796 | 1.31228175144583 | -3.13016269291068 |
| O | -2.16372592309683 | 0.36177521902336 | 0.40232839216167 |
| O | 0.35267633341644 | -0.77851700049362 | -0.16702080117783 |
| O | -1.58597321112664 | 3.09061031543428 | -3.53011572235563 |
| O | 2.15745963036323 | 0.18502681495582 | -3.98749218492679 |
| N | -2.84714461110657 | 2.78264232983531 | -0.95761874902852 |
| C | -1.23635355873197 | 0.93775318271391 | -0.48627169032821 |
| C | -0.38152108421932 | -0.13250886893050 | -1.17929033196613 |
| C | -2.01064757275912 | 1.75232701079480 | -1.53513234889763 |
| C | 0.52259598285667 | 0.54910237036974 | -2.22309433174600 |
| C | -1.00291182535885 | 2.34936065521292 | -2.53013877940336 |
| C | 1.30034630271475 | -0.45675011172896 | -3.08109261376054 |
| H | -0.55655876101877 | 1.60672602515440 | 0.06975401950391 |
| H | -1.05805742441914 | -0.83179929148232 | -1.69019352593042 |
| H | -2.66354285397107 | 1.05568177043078 | -2.08374313957275 |
| H | 1.23214620666727 | 1.20860663876694 | -1.69728428675242 |
| H | -0.32091438363107 | 3.03728974161369 | -1.99844985939169 |

| | | | |
|---|---|---|---|
| H | 0.57976588330377 | -1.09123877905277 | -3.61361711703365 |
| H | 1.94209335664890 | -1.07228366974523 | -2.44964319416918 |
| H | -2.30486315385225 | 3.60700029272797 | -0.72504996146937 |
| H | -3.27324175087970 | 2.42182478061730 | -0.10961709492065 |
| H | -1.67159023224759 | -0.19491443718628 | 1.02400454612507 |
| H | 0.73482070108905 | -1.60238394917866 | -0.49695282600340 |
| H | -2.16962106328500 | 2.52244384267028 | -4.05668699186029 |
| H | 1.63274696624212 | 0.80915342075174 | -4.51058210453930 |
| O | -6.22701487145765 | 1.52693130374771 | -1.19423749641831 |
| O | -4.25105370352641 | -0.06671483224413 | -2.62618182283840 |
| O | -9.24732517832026 | -1.55908951474229 | 4.41675653042848 |
| O | -8.02565057183195 | -3.40219447135675 | 4.09888081733237 |
| C | -6.20708776884066 | -1.54459893488566 | 0.68395847548765 |
| C | -5.84576906674995 | 0.28779204967932 | -0.88183298556008 |
| C | -6.47141542244949 | -0.25472011036671 | 0.23294665068443 |
| C | -5.26286778385959 | -2.32645305993358 | -0.03572777115782 |
| C | -4.86259750213624 | -0.50418903343507 | -1.62500037964626 |
| C | -4.63038777069034 | -1.83357554689601 | -1.12831282206672 |
| C | -6.84329280055397 | -2.12060417050543 | 1.83269160148229 |
| C | -7.77510312319251 | -1.54842761267792 | 2.62153875657128 |
| C | -5.69132846102143 | 2.26436893047695 | -2.27895099918394 |
| C | -8.33436630150249 | -2.27155082942744 | 3.75863601634273 |
| H | -7.18182826509300 | 0.37485524392425 | 0.74367081792956 |
| H | -5.05451040137670 | -3.32904623918389 | 0.30542562990704 |
| H | -3.91254459105621 | -2.42370092361011 | -1.67759746265934 |
| H | -6.54382280859545 | -3.12930874032118 | 2.09229644635758 |
| H | -8.16233728941384 | -0.55446652193826 | 2.47180938946485 |
| H | -5.68082823173801 | 1.67894334884910 | -3.19794555948404 |
| H | -4.66940002425196 | 2.59188933805615 | -2.04630638091769 |
| H | -6.34608545250085 | 3.12754208320805 | -2.37742029252238 |
| H | -9.61991153751174 | -2.04757575840683 | 5.17478588545482 |

Transitional state IX

| | | | |
|---|---|---|---|
| O | -0.37997238898484 | 0.58103722137745 | -0.79911965145516 |
| O | -3.21886133553748 | -2.39830279092577 | -1.57435433223185 |
| O | -1.28475545400813 | -2.70820270745586 | 0.47344039071619 |
| O | -1.36327784131937 | 1.99845684723690 | -2.26828099165343 |
| O | 1.41400943897565 | 0.25696303544452 | 1.47313455992376 |
| N | -3.60353307911855 | 0.24107516067456 | -2.65168742409873 |
| C | -2.53107096695660 | -1.28967721349105 | -1.05529633259119 |
| C | -1.15826522686969 | -1.69608073738809 | -0.49070359216158 |
| C | -2.34065408844373 | -0.15517444852858 | -2.06522882437547 |
| C | -0.51214289591680 | -0.46229780402128 | 0.15295772011762 |
| C | -1.61241861669601 | 1.00066107525118 | -1.34300927185166 |
| C | 0.88588818943974 | -0.77983177425117 | 0.68898863391942 |
| H | -3.13617554701989 | -0.92901686542732 | -0.21481475398316 |
| H | -0.50955575203696 | -2.04293118565259 | -1.31371362890468 |
| H | -1.68454689476134 | -0.49022913914810 | -2.87866267008859 |
| H | -1.15621027002016 | -0.12644705169109 | 0.98044068503714 |
| H | -2.26089080914640 | 1.36792283961994 | -0.51820987419506 |
| H | 1.54356208909127 | -0.99924726297690 | -0.16119652297275 |
| H | 0.81593426308750 | -1.65736805964727 | 1.33283460737839 |
| H | -3.43749339987649 | 0.99771573796344 | -3.30652070241667 |
| H | -4.22092757102048 | 0.59412420786865 | -1.92660707349257 |
| H | -2.87985884971041 | -2.60669860632397 | -2.45676727452930 |
| H | -1.99682649890338 | -3.30494214856780 | 0.18695227578632 |
| H | -0.73648133266068 | 2.64009920547719 | -1.90215566274127 |
| H | 1.46821950792511 | 1.05507834691711 | 0.92850465485308 |
| O | -10.69746264132134 | 0.80416180693735 | -1.40021810055548 |
| O | -10.42977891634089 | -1.32812594728967 | -3.34343792755973 |
| O | -4.98968820835406 | 2.00453517314674 | 2.66258721434198 |
| O | -3.76112499303473 | 0.68732768492787 | 1.34183085191400 |
| C | -7.31459660471807 | -0.31305398231433 | -0.85842082875113 |

| | | | |
|---|---|---|---|
| C | -9.60270853062280 | 0.06388187715988 | -1.59063967637608 |
| C | -8.52888165668673 | 0.36492704247855 | -0.76811562441021 |
| C | -7.18311833534768 | -1.35014719703163 | -1.82517003298968 |
| C | -9.48942219502023 | -1.01063116287839 | -2.58988596636301 |
| C | -8.21218691368565 | -1.67987311693446 | -2.64174633141454 |
| C | -6.17891309774321 | -0.00327109187836 | -0.04284804755051 |
| C | -6.09430174051040 | 0.93562509917907 | 0.92281023001829 |
| C | -11.87678768272961 | 0.64642228819887 | -2.16931879588357 |
| C | -4.84435598292226 | 1.17066270582190 | 1.63855144324624 |
| H | -8.66925164012733 | 1.15884181983521 | -0.05252670222351 |
| H | -6.23801890336645 | -1.86702202279198 | -1.89891232395125 |
| H | -8.12229939969027 | -2.46340863170494 | -3.37791365765333 |
| H | -5.29478830303418 | -0.59774695879205 | -0.23762600968214 |
| H | -6.92024636706038 | 1.55622832503819 | 1.22876274872809 |
| H | -12.29494829005555 | -0.35485911871822 | -2.05264010537393 |
| H | -11.68298639766451 | 0.81594178684662 | -3.22995539944924 |
| H | -12.56410016196779 | 1.39545722532273 | -1.78175641005329 |
| H | -4.15530407036918 | 2.15545410477946 | 3.14606589514249 |

Transitional state X

| | | | |
|---|---|---|---|
| O | -1.92893473708840 | 0.96298707896241 | -1.38090245381605 |
| O | -1.10771977447187 | -2.91660936325033 | -0.26005579080803 |
| O | 0.94951920295461 | -0.99369877573135 | -0.20109280745262 |
| O | -4.14471938527883 | 0.56191711658081 | -1.40463123653317 |
| O | 0.06624337907829 | 3.02418064014007 | -1.41825234929972 |
| N | -3.80796629560918 | -1.91626659048178 | -0.02772389290003 |
| C | -1.34991011760295 | -1.53558218099608 | -0.14308098531172 |
| C | -0.26536804552588 | -0.71754777619240 | -0.85701725379783 |
| C | -2.73893016725701 | -1.24564080540798 | -0.73759563998225 |
| C | -0.65086313674543 | 0.77098293044223 | -0.79882273746854 |
| C | -2.97266172516924 | 0.27278921630464 | -0.73122607916131 |
| C | 0.32989002073810 | 1.65584320841930 | -1.57882367605127 |
| H | -1.35369560374096 | -1.24269784861008 | 0.92138652676124 |
| H | -0.22889243859400 | -1.03567714034627 | -1.90823770588562 |
| H | -2.75792232012004 | -1.58969170451672 | -1.77799786741481 |
| H | -0.67609325250469 | 1.08825060808746 | 0.25639990315766 |
| H | -3.02617274938269 | 0.61496157679085 | 0.32591658500236 |
| H | 0.28788400186259 | 1.37278314062181 | -2.63810826545061 |
| H | 1.33942243663856 | 1.50615171353097 | -1.19424103834208 |
| H | -3.81845894186805 | -1.59501991519035 | 0.93874439382750 |
| H | -3.60553609334285 | -2.91051133148921 | -0.01635415272008 |
| H | -0.20766837434197 | -3.09499106648925 | 0.05145377256006 |
| H | 1.69967943859286 | -0.72036832686423 | -0.74602875274600 |
| H | -4.08784420586263 | 1.46228319473680 | -1.75997737354728 |
| H | -0.83028811905509 | 3.20652470736989 | -1.73253178951187 |
| O | -7.77447245145108 | -0.61438706276976 | -3.56023430386617 |
| O | -10.66368587493483 | -0.53463331390166 | -3.34089314659119 |
| O | -5.86565631611934 | 0.92932019623018 | 3.47875783576402 |
| O | -4.18691062263160 | -0.11076523973024 | 2.50631695144970 |
| C | -8.25722586543236 | 0.02980786274492 | -0.04964321236287 |
| C | -8.45706145735758 | -0.40272377111759 | -2.43334065055463 |
| C | -7.68501706389276 | -0.21020939686365 | -1.29732763815956 |
| C | -9.67675117790031 | 0.08498951709384 | 0.04680172832931 |
| C | -9.92504191803043 | -0.36175941965827 | -2.35052623800793 |
| C | -10.46486327027490 | -0.10266791719394 | -1.03887372463607 |
| C | -7.48089716580413 | 0.23260107811242 | 1.14209405457607 |
| C | -6.14016384282084 | 0.10895220561370 | 1.22316069900093 |
| C | -8.40103200702490 | -0.84394042603257 | -4.80899916530493 |
| C | -5.33823312901496 | 0.29400343200290 | 2.43279962018261 |
| H | -6.61365860847970 | -0.24959724546200 | -1.42133756789942 |
| H | -10.12808948021294 | 0.27455003982536 | 1.00895031358413 |
| H | -11.54137781193943 | -0.06742272289307 | -0.97218913965569 |
| H | -8.06752449476912 | 0.47029220810216 | 2.02157483208583 |

| | | | |
|---|---|---|---|
| H | -5.55816257380557 | -0.20514480962148 | 0.37053645731870 |
| H | -9.01461517349067 | 0.00833773504734 | -5.10734441170037 |
| H | -9.03767835809336 | -1.73004263071799 | -4.77687660419511 |
| H | -7.58113375300349 | -0.98841969570459 | -5.50934465843341 |
| H | -6.76734442150134 | 1.26135440680918 | 3.31758511855196 |

Transitional state XI

| | | | |
|---|---|---|---|
| O | -0.89476880086293 | 0.63489345545170 | -1.60288523346307 |
| O | -3.38545544523535 | -1.17835949998706 | 1.16286634076720 |
| O | -0.73773955108488 | -2.05312210545608 | 0.83607236686198 |
| O | -2.32462194031373 | 2.36694011209636 | -1.90786592212996 |
| O | 1.79654017785521 | -0.42099801956691 | -1.97001271838301 |
| N | -3.92334725571371 | 1.60329522713377 | 0.35074148716291 |
| C | -2.40885732442987 | -0.30687065310830 | 0.65638263438091 |
| C | -1.37007611982430 | -1.18121863449028 | -0.06292192520151 |
| C | -2.98171071745971 | 0.73068451170999 | -0.31966834099703 |
| C | -0.30166170759441 | -0.30252039028711 | -0.72161023740238 |
| C | -1.80720951243207 | 1.49933699635347 | -0.96516401297981 |
| C | 0.68172446255034 | -1.15156229477941 | -1.53307426364776 |
| H | -1.90538497533345 | 0.22234741744259 | 1.48197104158668 |
| H | -1.89085035434651 | -1.75268806104531 | -0.84749956115780 |
| H | -3.51814935742168 | 0.21301667868475 | -1.12280539404833 |
| H | 0.25063618154212 | 0.23157973886335 | 0.06829645093051 |
| H | -1.26499570889967 | 2.05779854512544 | -0.16655795861626 |
| H | 0.14397107427641 | -1.59199021796719 | -2.38110043533629 |
| H | 1.05980413386678 | -1.94552305621920 | -0.88785517019320 |
| H | -4.27674790508556 | 2.28080497010517 | -0.31609892792919 |
| H | -3.45347706425451 | 2.11317035891022 | 1.09260727817447 |
| H | -3.96897765734996 | -0.69025872173373 | 1.78123558117743 |
| H | -1.43066757979349 | -2.49916279794339 | 1.34978140564626 |
| H | -1.61361134314913 | 2.71084809491013 | -2.46984086696400 |
| H | 1.49129616270910 | 0.28625964384006 | -2.55568744872086 |
| O | -10.25249767909508 | -0.05855598492570 | -2.70490962113554 |
| O | -8.85563846546541 | -1.83285627062531 | -4.52474127691048 |
| O | -6.92255007705405 | 1.49712836804023 | 3.31925306399099 |
| O | -5.19862603041308 | 0.18213456603467 | 2.81221427916423 |
| C | -7.21058722528910 | -0.76800301656991 | -0.90349243520237 |
| C | -9.05286520787102 | -0.60583066368105 | -2.48574224222174 |
| C | -8.45810694133470 | -0.27265543017510 | -1.27940594934139 |
| C | -6.53574124150362 | -1.64442057077507 | -1.79956505898828 |
| C | -8.36689190304607 | -1.50499423580264 | -3.42495365168858 |
| C | -7.07995085877704 | -1.99068300617125 | -2.99086233993719 |
| C | -6.56959709030172 | -0.44490435989986 | 0.33719741616321 |
| C | -7.03047188162172 | 0.39316429852763 | 1.28800014911700 |
| C | -11.00335593316773 | -0.29815055870238 | -3.88214596095089 |
| C | -6.28449860535123 | 0.65656261324912 | 2.51310808516946 |
| H | -9.00873918090398 | 0.39009187686516 | -0.63187744454582 |
| H | -5.57102364187120 | -2.03176361055269 | -1.50814347471598 |
| H | -6.57348002192994 | -2.65338897607536 | -3.67570505063616 |
| H | -5.61539925681055 | -0.92440282539923 | 0.50948531098763 |
| H | -7.96494794101435 | 0.92493135551144 | 1.21043397079924 |
| H | -11.23054490398286 | -1.35935743375814 | -3.99813939866532 |
| H | -10.46482999072516 | 0.03702343812137 | -4.77033709049735 |
| H | -11.91791640503935 | 0.27620543471000 | -3.75042033812965 |
| H | -6.42633271850795 | 1.67272454064625 | 4.14107639983853 |

Transitional state XII

| | | | |
|---|---|---|---|
| O | -2.88553470199644 | 2.93924812621734 | -7.02361934862814 |
| O | -1.56723899019921 | 0.27258827967701 | -4.15029332225883 |
| O | -0.05270366781631 | 0.67932647112009 | -6.43567635123547 |
| O | -4.51425925006602 | 3.69313064708174 | -5.65494026179445 |
| O | -1.43530906444884 | 3.43377391952711 | -9.42960864233797 |
| N | -3.23250547343653 | 2.59350452517414 | -3.38354363860872 |

| | | | |
|---|---|---|---|
| C | -1.65278022936238 | 1.46418981359984 | -4.89192856724599 |
| C | -1.36673136807427 | 1.17765254154433 | -6.37565040604752 |
| C | -3.04588074051271 | 2.08556054236387 | -4.72994808845289 |
| C | -1.55995589184492 | 2.46437498586766 | -7.19350876913128 |
| C | -3.19745166615033 | 3.27459188448126 | -5.69039274307159 |
| C | -1.35770210380042 | 2.23991987189550 | -8.69755445300040 |
| H | -0.90330663579103 | 2.18308563866824 | -4.52615797068673 |
| H | -2.09277538194721 | 0.42904562024079 | -6.72471562217585 |
| H | -3.79748804705747 | 1.32924531624714 | -5.01555580194116 |
| H | -0.84855126434328 | 3.22672208080629 | -6.83832163242629 |
| H | -2.50830479135214 | 4.07608133413318 | -5.35362279728653 |
| H | -2.10664681091441 | 1.51868198294769 | -9.04860665149234 |
| H | -0.35441709440039 | 1.85158396037863 | -8.87668972580285 |
| H | -3.94693948422680 | 3.31619060707764 | -3.40006219777965 |
| H | -0.73663606803270 | -0.16217223382191 | -4.40380589388520 |
| H | 0.10739020570164 | 0.23669816588798 | -7.27949050654135 |
| H | -4.62824489596026 | 4.46736728081463 | -6.22842805852246 |
| H | -2.30988568549804 | 3.82368550177079 | -9.29256999001547 |
| O | -8.00301625644648 | 1.11807209264836 | -4.48845450623235 |
| O | -7.61530222379634 | -1.53879455474840 | -4.62253644107942 |
| O | -4.92536432643614 | 3.73158544140740 | 0.36401738584790 |
| O | -5.33367682030364 | 3.93887976393742 | -1.80770414815382 |
| C | -4.80672575051594 | 0.78324180722455 | -2.75073095686003 |
| C | -6.95462425098305 | 0.61391712298166 | -3.85941443174389 |
| C | -5.91030589745125 | 1.37417806852072 | -3.32225896305018 |
| C | -4.76521001513731 | -0.63150367309252 | -2.65587977009459 |
| C | -6.85559626384742 | -0.85751364014312 | -3.90697650868068 |
| C | -5.73963196768232 | -1.41204548612526 | -3.18656458243259 |
| C | -3.58952460617177 | 1.60274150366385 | -2.37418085671168 |
| C | -3.70367539499175 | 2.31316193190789 | -1.01811579317095 |
| C | -8.04165215074543 | 2.49895259242806 | -4.82389933476604 |
| C | -4.74784829208089 | 3.38930339460030 | -0.89738147052165 |
| H | -5.96247730107753 | 2.44965507586725 | -3.38602374135802 |
| H | -3.91418957113380 | -1.09903396932549 | -2.18510281986687 |
| H | -5.67897798654387 | -2.48792056674786 | -3.14281306449864 |
| H | -2.74330408350924 | 0.91029305767197 | -2.30857282377908 |
| H | -3.87238041184004 | 1.59887623889056 | -0.21310581931026 |
| H | -8.91857380638891 | 2.60964946480712 | -5.45629083543552 |
| H | -7.13243783026401 | 2.77995147305670 | -5.36044348347823 |
| H | -8.15291791440040 | 3.11693846438829 | -3.93172391038781 |
| H | -5.56465209843777 | 4.45952925754233 | 0.47835872779451 |
| H | -2.74797003600219 | 2.80384067943102 | -0.82016450177436 |
| O | -9.39509426368295 | 1.12844118059116 | -2.52797759655234 |
| H | -9.25150360606263 | -1.41950217168294 | -2.58775265038769 |
| H | -7.75235474476928 | 3.86982543019713 | -1.86071093208281 |
| H | -9.29529857846187 | 2.99888793596077 | -1.61832916301663 |
| C | -8.63024458004718 | 0.46504603605994 | -1.77965369889602 |
| C | -8.61987789646586 | -0.97732031251778 | -1.83090017964683 |
| C | -8.40393825864263 | 3.38322299379199 | -1.12850517225877 |
| H | -8.67593307323663 | 4.09717649070782 | -0.35223171335528 |
| C | -7.76602563420588 | 1.05418775427796 | -0.76304793068658 |
| C | -7.92307684769872 | -1.74377995205667 | -0.94768068802435 |
| O | -7.66289600199245 | 2.36184661681657 | -0.47349497616986 |
| H | -7.97332184673557 | -2.81889150721663 | -1.01861875271196 |
| C | -7.03764601937055 | 0.22988292657626 | 0.09055096928637 |
| C | -7.11602548267169 | -1.15088930020204 | 0.03684581823879 |
| H | -8.15429264097978 | -3.32478587601155 | 1.47364141219184 |
| H | -6.44453370179515 | 0.72586783705684 | 0.84568099271519 |
| O | -9.60345393623427 | -1.88074482970026 | 2.67308822392904 |
| C | -6.42522839444690 | -2.06255636744760 | 1.02964048901787 |
| O | -3.87407297362778 | -3.06186192266140 | -0.20279755065435 |
| H | -5.98716422509392 | -2.89476280723877 | 0.46768780439719 |
| C | -7.48750030232509 | -2.63795924613478 | 1.99058834949955 |

| | | | |
|---|---|---|---|
| H | -10.15504194002162 | -1.19689479786746 | 3.10068172855712 |
| H | -3.26522586097137 | -3.79385940690414 | -0.38683103838914 |
| C | -8.32160925237768 | -1.55496110845183 | 2.62227900074070 |
| H | -4.44499274604971 | -0.56263101535644 | 0.12744811210984 |
| H | -2.05430215853345 | -1.25789066654544 | -0.71054602195174 |
| C | -4.18909459618877 | -1.05333516762386 | 1.08050451212377 |
| O | -7.90214098240077 | -0.50232760205232 | 3.05119624602420 |
| C | -3.27066347283191 | -2.24258683769685 | 0.76906177037370 |
| N | -5.36046033810912 | -1.48367781126109 | 1.81995251618164 |
| H | -6.96522603861498 | -3.17032631687037 | 2.78659500059000 |
| C | -1.90181983768048 | -1.75682974554258 | 0.25691785343188 |
| H | -0.35312994117500 | -2.73049808595010 | -0.46675340086058 |
| O | -1.10495244048018 | -2.90834770761491 | 0.11321848794426 |
| H | -5.73219997747263 | -0.71014478600521 | 2.36481440490209 |
| H | -0.18905871643985 | 0.42056931968997 | -0.18603862986721 |
| H | -3.11043922474369 | -2.81395629887202 | 1.69541388193095 |
| O | -4.23172003588340 | 1.07963586325872 | 2.08889954202005 |
| C | -3.44216364796552 | -0.04685473840466 | 1.96821257240449 |
| O | -2.20349510106437 | 0.34137205207889 | 1.41900256534779 |
| C | -1.30387878510052 | -0.73947467241861 | 1.24235569867514 |
| C | 0.01362297184606 | -0.13996816111156 | 0.73491795589818 |
| H | 0.72662615985030 | -0.94043070896045 | 0.53454675756895 |
| H | -3.82705334365040 | 1.71459457578911 | 2.69876050412393 |
| H | -3.26380175892928 | -0.52778365836392 | 2.95136108572139 |
| H | -1.13114885411616 | -1.23886559466986 | 2.20897377820044 |
| O | 0.62907857196332 | 0.68061041704276 | 1.69269472300194 |
| H | 0.01991441092389 | 1.39646042361628 | 1.92115666853319 |

Transitional state XIII

| | | | |
|---|---|---|---|
| O | -2.19714130518866 | 3.72219272025207 | -7.08840465023823 |
| O | -2.22665646153454 | 0.04874632717159 | -5.31491379625659 |
| O | 0.02692716089098 | 0.87639629134779 | -7.09608592088163 |
| O | -3.80023116291681 | 4.48034046170398 | -5.64826819354541 |
| O | -0.04172642101560 | 4.44039114288391 | -9.00420540175697 |
| N | -3.08280562795052 | 2.48783249676737 | -3.75813333839074 |
| C | -1.74106896021980 | 1.37139358704105 | -5.49835268587363 |
| C | -1.22491775718545 | 1.52044674873714 | -6.94779761494191 |
| C | -2.90389978943114 | 2.33341415281768 | -5.20794852193415 |
| C | -0.99323021983538 | 2.99939849745770 | -7.29586080795034 |
| C | -2.61564964405214 | 3.75076073369911 | -5.73213560611319 |
| C | -0.59868010443478 | 3.16850995855130 | -8.77050059147611 |
| H | -0.91186254228354 | 1.57226650266467 | -4.79910681532565 |
| H | -1.97027160268049 | 1.08741938341615 | -7.63531944602060 |
| H | -3.81227795068956 | 1.95126843606851 | -5.69974077840967 |
| H | -0.19293106790943 | 3.40628480559388 | -6.65405709551477 |
| H | -1.81642786331578 | 4.21940713266102 | -5.11939971876961 |
| H | -1.48247731550388 | 2.99471349764138 | -9.40164772390227 |
| H | 0.16811402012235 | 2.42773606699207 | -9.01970906518259 |
| H | -3.58074644496430 | 3.36373970208084 | -3.59498924401114 |
| H | -1.50846875134797 | -0.58149607383021 | -5.43843252044930 |
| H | -0.06536940425477 | -0.07928825301916 | -7.05398311614067 |
| H | -3.66569728710964 | 5.35017908997581 | -6.04202990332652 |
| H | -0.69312968264732 | 5.12419458372000 | -8.81656640032566 |
| O | -8.07089419967105 | 3.20940728498439 | -4.89971198647325 |
| O | -9.19260824604813 | 0.76757191386256 | -4.62480461387476 |
| O | -4.06632928996544 | 3.36435952367578 | 0.02541872826263 |
| O | -5.19563058828807 | 3.66853718111092 | -1.86003831016845 |
| C | -5.20908525465556 | 1.17040590092228 | -3.68812075489008 |
| C | -7.27928852258284 | 2.17195993849702 | -4.53447224884141 |
| C | -5.97714660557965 | 2.28764449075115 | -4.17964193655846 |
| C | -5.73526449481681 | -0.06568313476309 | -3.69992121110343 |
| C | -7.99778747911001 | 0.88265525923454 | -4.45493214772972 |
| C | -7.08920410701374 | -0.33891380450766 | -4.27876248198915 |

| | | | |
|---|---|---|---|
| C | -3.81485563572097 | 1.39841166075516 | -3.11710347189178 |
| C | -3.83942040784221 | 1.67159784430272 | -1.60302664441148 |
| C | -7.55778789660614 | 4.52952369971638 | -4.80960365450629 |
| C | -4.44289487577911 | 2.99427966272571 | -1.20093186508148 |
| H | -5.49414943948548 | 3.25332674354293 | -4.21677360938384 |
| H | -5.19171437642005 | -0.92578716539147 | -3.32903617772634 |
| H | -6.93039736992626 | -0.65599245816546 | -5.32777992964008 |
| H | -3.23812490529319 | 0.47637180545342 | -3.25589777002687 |
| H | -4.40923751860779 | 0.89112782772901 | -1.08945299830058 |
| H | -8.37715956954959 | 5.19542652990300 | -5.07751947572673 |
| H | -6.72412355455493 | 4.67667980728985 | -5.50316948036863 |
| H | -7.22247515465573 | 4.74765854116468 | -3.79088629371304 |
| H | -4.46026581061083 | 4.21947508703616 | 0.24810664323532 |
| H | -2.82271280825868 | 1.63090125330975 | -1.20571920656200 |
| O | -7.76129624152367 | -1.42906628579529 | -3.66971814793476 |
| H | -7.53109528113251 | -3.73248197064758 | -2.61970101529912 |
| H | -7.78041302289333 | 2.68921179771161 | -1.52837379621075 |
| H | -8.53080298569553 | 1.76051704900180 | -0.19904027238494 |
| C | -7.62182860644138 | -1.62612547344057 | -2.31356136949727 |
| C | -7.49131533295840 | -2.93954565807726 | -1.88372254230941 |
| C | -7.71101561301803 | 1.78303122848837 | -0.92425999960421 |
| H | -6.75700680576356 | 1.78507303853050 | -0.39056358176829 |
| C | -7.60102495256120 | -0.58128357175532 | -1.37280546528279 |
| C | -7.26296219219841 | -3.22451970677995 | -0.54564179839620 |
| O | -7.79458876355158 | 0.69252094453545 | -1.82881349962155 |
| H | -7.12806728998740 | -4.25266962293122 | -0.23350233997388 |
| C | -7.35645220871363 | -0.87977507315711 | -0.03875418779789 |
| C | -7.15429469650848 | -2.19323749506047 | 0.38084091253061 |
| H | -8.55298735604232 | -3.56778157479595 | 2.35423720776744 |
| H | -7.30026934209979 | -0.07783645662511 | 0.68303429846233 |
| O | -9.85925569260242 | -1.92858661901501 | 3.76474349125890 |
| C | -6.73770945894234 | -2.52145222695892 | 1.80507617446009 |
| O | -3.86870018667312 | -3.56139749738322 | 1.60586432430354 |
| H | -6.17803697382063 | -3.46432481767257 | 1.77753222614672 |
| C | -7.92723068257877 | -2.76214356810119 | 2.74437927638377 |
| H | -10.41515567185605 | -1.17445641172345 | 3.99513731463611 |
| H | -3.26482068572072 | -4.21410208061201 | 1.97423729594738 |
| C | -8.81524681189880 | -1.57297220614243 | 3.01758736828454 |
| H | -4.78630643836100 | -1.39208428040752 | 0.55340083683244 |
| H | -2.18414322429086 | -2.01193799901877 | 0.39891344204736 |
| C | -4.62606570095264 | -1.28589765203089 | 1.63786291170773 |
| O | -8.63812832707115 | -0.43876808455949 | 2.65043978656968 |
| C | -3.52962978425972 | -2.26830329409087 | 2.08067744343839 |
| N | -5.86104305558837 | -1.51001522686745 | 2.40001979004266 |
| H | -7.54900008187188 | -3.09064730345992 | 3.71604961210283 |
| C | -2.16175059982913 | -1.85284929063329 | 1.48941839815610 |
| H | -1.11283077554380 | -3.50414126735769 | 1.75107672539521 |
| O | -1.11719207597367 | -2.60172728903519 | 2.08187852060310 |
| H | -6.36755333298289 | -0.62381941888256 | 2.44908792653742 |
| H | -0.79325058712266 | 0.35165939989927 | 0.06436573674408 |
| H | -3.46251419179081 | -2.27392700092718 | 3.18164722480696 |
| O | -5.12915185707506 | 1.02500807156406 | 1.38278560678105 |
| C | -4.18415987611344 | 0.15778006012193 | 1.92590047235637 |
| O | -2.93305241930535 | 0.42547749849872 | 1.30691560990508 |
| C | -1.86725404120531 | -0.37479687754680 | 1.79628194611448 |
| C | -0.57404329326781 | 0.09889907353933 | 1.11269981710898 |
| H | 0.15896948336862 | -0.71390490163347 | 1.13745558195519 |
| H | -4.75816915137582 | 1.92172227825263 | 1.38419252193148 |
| H | -4.08544995307879 | 0.31526249454425 | 3.02119042647976 |
| H | -1.76801923641145 | -0.24414901784650 | 2.88780034554354 |
| O | -0.00050674334467 | 1.18865189464212 | 1.79583176512795 |
| H | -0.57972112689844 | 1.95721503751255 | 1.75245254378634 |

The structure of the intermolecular ferulic acid complex with $NO_2^{\bullet}$

| | | | |
|---|---|---|---|
| O | -3.11953607361865 | 1.38980948301554 | 0.01392708074157 |
| O | -4.21226201317614 | -1.07312144075667 | 0.00341728732338 |
| O | 4.24468329443014 | 1.46652216239565 | 0.28756000063305 |
| O | 4.17997153981275 | -0.75533235071404 | 0.14660747705020 |
| C | -0.08207734483188 | -0.72650724393658 | 0.05967112744788 |
| C | -2.26824275415514 | 0.32238899746616 | 0.04465928170942 |
| C | -0.89184486551665 | 0.41536459241091 | 0.07526518004230 |
| C | -0.70003948399056 | -1.97854214959227 | 0.01146530996421 |
| C | -2.89015994670336 | -0.95579857900528 | 0.01500911246778 |
| C | -2.07100314119397 | -2.09005582510151 | 0.00852773605680 |
| C | 1.36353211367173 | -0.64747410035903 | 0.09265329425133 |
| C | 2.13001070258484 | 0.44421280098655 | 0.16934879052050 |
| C | -2.57471990105083 | 2.69100808227144 | 0.12027381962236 |
| C | 3.63375938701487 | 0.37149887527159 | 0.20324332501371 |
| H | -0.41536700973564 | 1.38128494170937 | 0.10892252172468 |
| H | -0.08689224316223 | -2.86702352888119 | -0.00394594793965 |
| H | -2.55669236791350 | -3.05358119774007 | -0.00987950381091 |
| H | 1.89367395343725 | -1.59355572244991 | 0.05496964832672 |
| H | 1.73823850116232 | 1.45095648625514 | 0.21868669125085 |
| H | -1.92170895713996 | 2.90924501129554 | -0.72954521644155 |
| H | -2.02211279190332 | 2.80701196006772 | 1.05648986924453 |
| H | -3.43652241467094 | 3.35439710519674 | 0.11309186953003 |
| N | -4.93211310060392 | -0.59580850201927 | 1.45909416395001 |
| O | -4.07780131996998 | -0.14834051297415 | 2.24368701084617 |
| O | -6.02717815153190 | -0.06656624704035 | 1.21965873732735 |

Structure of the intermolecular complex of the ferulic acid dimer (XIV) with $NO_2^{\bullet}$

| | | | |
|---|---|---|---|
| C | 2.96878524492477 | -0.88927278331074 | 2.32027872511507 |
| C | 1.76402874839009 | -0.29986010767264 | 1.98232509487744 |
| C | 0.68850171721281 | -0.41423744937966 | 2.85273415586590 |
| C | 3.12938452153595 | -1.62422834359793 | 3.49031322286137 |
| C | 0.83349960494394 | -1.11295263389947 | 4.02789438743049 |
| C | 2.04069480481717 | -1.73085110977687 | 4.38474849145276 |
| O | 4.38608349613592 | -2.11966609140212 | 3.67332647288651 |
| C | 4.66321621488717 | -3.03979021038663 | 4.71806742137304 |
| O | 2.11696779796953 | -2.39766063604422 | 5.54474156095086 |
| C | 1.63023122333663 | 0.39698079459849 | 0.65534892190813 |
| C | 1.35589593476554 | -0.59252917277408 | -0.50974022340252 |
| C | 0.95404409765794 | -2.02999859226983 | 0.00458761975111 |
| C | 0.20534837093662 | 0.05528918246037 | -1.20354973400515 |
| C | -0.22089235530723 | 1.12636203333831 | -0.42977676728804 |
| C | -1.33953292622132 | 1.87766542660981 | -0.78729571960421 |
| C | -0.45909293196658 | -0.29019867874885 | -2.35936693970053 |
| C | -2.00216342253958 | 1.51565679912269 | -1.95136062297416 |
| C | -1.57005857259994 | 0.45896847275119 | -2.75451342685008 |
| O | 0.55987211217449 | 1.35151565202578 | 0.66828340822430 |
| O | 1.82390722538886 | -2.90983391665984 | -0.13617062268884 |
| O | -0.18534706746005 | -2.17534897956770 | 0.47710733710812 |
| O | -1.69736681729863 | 2.91486243147818 | 0.03195066771200 |
| C | -2.80828501748932 | 3.70722645437380 | -0.34788784813904 |
| C | -2.25640672537733 | 0.10792590294444 | -3.99028791601309 |
| C | -3.11419983509368 | 0.84920892268292 | -4.68957901675139 |
| C | -3.58462403721945 | 2.26540788912880 | -4.39527844354384 |
| O | -2.81052870265013 | 3.20278043714940 | -4.68507960140733 |
| O | -4.74220002387007 | 2.38701733853834 | -3.93215834081064 |
| H | 3.81371372571158 | -0.83398422793295 | 1.65028952521544 |
| H | -0.25955786071247 | 0.02975608276326 | 2.59106934820049 |
| H | -0.00003149396499 | -1.24210219887361 | 4.70361523015563 |
| H | 5.70073162930211 | -3.32470082132812 | 4.55842673437119 |
| H | 4.01115257668837 | -3.91515682592175 | 4.65520920666506 |
| H | 4.53006431675406 | -2.57196090872213 | 5.69554101659383 |

| | | | |
|---|---|---|---|
| H | 2.55452740889776 | 0.95913410391004 | 0.45172279040047 |
| H | 2.23849440826138 | -0.72988170404228 | -1.13871177899539 |
| H | -0.12130606418581 | -1.13168120899530 | -2.94583234679188 |
| H | -2.88377456693584 | 2.05364331680630 | -2.25531122284184 |
| H | -2.85861936952256 | 4.49214669887424 | 0.40250182694085 |
| H | -3.73176138988409 | 3.12123926332779 | -0.34625672943739 |
| H | -2.66690653908916 | 4.13872092483298 | -1.34330454894420 |
| H | -2.00009778832044 | -0.87850952291203 | -4.36495463940080 |
| H | -3.55881878890425 | 0.44134357112360 | -5.58926854204898 |
| N | 1.29040531392459 | -3.88071668064518 | 5.49737912260131 |
| O | 0.06325499681695 | -3.68399092078922 | 5.53413002014623 |
| O | 1.85796595339922 | -4.64761176781370 | 4.69301026588253 |

Structure of the intermolecular complex of the ferulic acid (XIV) dimer with $NO_2^{\bullet}$

| | | | |
|---|---|---|---|
| C | 0.19216271515761 | -0.92778311872518 | 2.68539583696181 |
| C | 1.25231743842198 | -0.08085820513683 | 2.60341506597933 |
| C | 2.37959314919273 | -0.30292921906287 | 3.45652008371588 |
| C | 0.15286424804751 | -1.97634116875593 | 3.71189502559844 |
| C | 2.51939078397145 | -1.42202636384154 | 4.22931996183729 |
| C | 1.50140123864895 | -2.43399808859950 | 4.25312402721939 |
| C | 1.33896219683727 | 1.02842923496913 | 1.67487869176466 |
| C | 0.48691479909528 | 1.39970131876867 | 0.70634304684770 |
| C | 0.74557742029374 | 2.68179407687203 | -0.08833424306295 |
| O | -0.72072516865819 | 0.78404035210175 | 0.49114194506131 |
| C | -1.16582884980489 | 0.70023614894202 | -0.81296423516217 |
| C | -0.28385562358142 | 0.36300333251439 | -1.83443526734007 |
| C | -2.52543579935025 | 0.85263243674525 | -1.11231284827485 |
| C | -0.72320801380644 | 0.14306199800687 | -3.12096077224399 |
| C | -2.95492830277405 | 0.60472954390937 | -2.40899504015934 |
| C | -2.08037893890797 | 0.23119698347125 | -3.42706253799185 |
| C | -2.61203452514944 | -0.01647591272988 | -4.75980749558751 |
| C | -2.00800752157261 | -0.62295352967662 | -5.77995942087892 |
| C | -0.62174324411429 | -1.24522686065807 | -5.81726086275457 |
| O | 0.27184673977946 | -0.59158935178896 | -6.40357708309325 |
| O | -0.49362983461068 | -2.38606708980185 | -5.32198011745515 |
| O | -0.26309282397403 | 3.24853662607117 | -0.56122634812981 |
| O | 1.92436512505489 | 3.08016491031174 | -0.16192428266345 |
| O | 1.60900851882932 | -3.57563069792807 | 4.74922660406334 |
| O | 3.73674108281700 | -1.60253790832392 | 4.84731493097605 |
| O | -3.49926992144179 | 1.21963390729054 | -0.22240712486453 |
| C | -3.14364933453288 | 2.03008324015867 | 0.88708554436855 |
| C | 3.75401073184461 | -2.28180189116642 | 6.09641774841235 |
| H | -0.66287082864000 | -0.82526787857408 | 2.03968514956233 |
| H | 3.19624516888310 | 0.40205507071448 | 3.43278583268625 |
| H | -0.48373933396021 | -2.83378031122750 | 3.48029187220864 |
| H | 2.21656424457641 | 1.65683187693406 | 1.75072261756742 |
| H | 0.76759659411539 | 0.28293466949961 | -1.58896576647147 |
| H | -0.00787400682141 | -0.10307127962488 | -3.88991448477068 |
| H | -4.00926238680817 | 0.71852222368979 | -2.60842796159278 |
| H | -3.62846372737257 | 0.33610907494968 | -4.89850098513064 |
| H | -2.52898250297475 | -0.72244391158171 | -6.72441948495094 |
| H | -2.73227307371083 | 1.43068395324420 | 1.70397677142871 |
| H | -4.07139335780100 | 2.50460822049644 | 1.19988478201346 |
| H | -2.40868048125253 | 2.78679828962895 | 0.59957175965544 |
| H | 4.77176061684971 | -2.16538304605849 | 6.45976895128564 |
| H | 3.49911419877312 | -3.33651020162886 | 5.97622733349975 |
| H | 3.04263269693330 | -1.82508748401224 | 6.79073182910420 |
| N | -0.34081512056378 | -1.46664149691552 | 5.03364689988571 |
| O | -0.06956436191557 | -0.28882104388065 | 5.37116382394699 |
| O | -0.76426345699492 | -2.31629951523380 | 5.85685498863582 |

The structure of the intermolecular ferulic acid-glucosamine (XII) complex with $NO_2^{\bullet}$

| | | | |
|---|---|---|---|
| O | 2.33999527202169 | 0.15525298940590 | -5.55978570242024 |
| O | 3.27442532622808 | 0.36649861748955 | -1.48799147414050 |
| O | 4.98328736508511 | -1.00908957239757 | -3.35829267049004 |
| O | 0.62827855234416 | 1.58901630351833 | -5.33294668526782 |
| O | 4.01626276074320 | -1.25860007577572 | -7.37887030222617 |
| N | 1.43561718272992 | 2.27110190529828 | -2.66750659491869 |
| C | 3.23415090574937 | 0.62439600694723 | -2.87761693706481 |
| C | 3.66914217895843 | -0.62084576345837 | -3.67329685718103 |
| C | 1.82331353721033 | 1.03456900563967 | -3.32894167094244 |
| C | 3.62381560053009 | -0.30043542770569 | -5.16930589794177 |
| C | 1.88056773949262 | 1.27667384506824 | -4.84378879700779 |
| C | 3.93689849982382 | -1.54306755313549 | -6.01093323594154 |
| H | 3.90807190068000 | 1.46361826459630 | -3.10518904817264 |
| H | 2.94541579790976 | -1.41802447315987 | -3.45018574456843 |
| H | 1.12448779364869 | 0.20663969610955 | -3.12141663311515 |
| H | 4.36546535789263 | 0.48839021821327 | -5.37430090451994 |
| H | 2.58763922954177 | 2.11900592278626 | -5.00641478244885 |
| H | 3.16159966650616 | -2.29146278654214 | -5.80262530954415 |
| H | 4.91145995354297 | -1.92777284146691 | -5.70837761017445 |
| H | 0.84535962283341 | 2.83700406467907 | -3.27967366680549 |
| H | 4.21125616502852 | 0.35864649446133 | -1.22964847058405 |
| H | 4.98931547168816 | -1.47273292551361 | -2.50935420571326 |
| H | 0.70766951983871 | 1.79552563121192 | -6.28083783783042 |
| H | 3.17330615925185 | -0.87251934817168 | -7.65673404304248 |
| O | -3.36431869382984 | 0.54436608919827 | -3.84289207515131 |
| O | -4.06555417856679 | -0.91675271473412 | -1.71303121504091 |
| O | -1.86000377289830 | 4.45455607999085 | -1.17462672075899 |
| O | -0.56853704904284 | 4.32258860135391 | -2.97109180957925 |
| C | -0.58240265263847 | 1.27031153285899 | -1.53045432665607 |
| C | -2.55137539363584 | 0.58377705556263 | -2.74277578299325 |
| C | -1.36554791657114 | 1.26802373450495 | -2.68919246755980 |
| C | -1.03768033956893 | 0.57902213257517 | -0.42448873535011 |
| C | -3.04566365717266 | -0.17260421159646 | -1.60962453180661 |
| C | -2.24914566675135 | -0.09490484788263 | -0.41617146830473 |
| C | 0.68469362154954 | 2.09552649308262 | -1.43210249108950 |
| C | 0.31463972261988 | 3.48815416763991 | -0.89672565364158 |
| C | -2.97006332546032 | 1.28261764848764 | -4.98982728812276 |
| C | -0.80404018839620 | 4.13543096166877 | -1.75780403361938 |
| H | -1.01853466818387 | 1.82444491631933 | -3.54610149160331 |
| H | -0.45255325301556 | 0.59299378069777 | 0.49076712379514 |
| H | 1.35108051200125 | 1.59542143988423 | -0.71285344981292 |
| H | -0.05833084487086 | 3.40185042467149 | 0.12568428828302 |
| H | -3.76236076905618 | 1.10364541831037 | -5.71205960935867 |
| H | -2.00698677147352 | 0.92564786593472 | -5.36405732083229 |
| H | -2.88946971912438 | 2.34841310947722 | -4.75527862873132 |
| H | 1.20015435605309 | 4.12485829350268 | -0.92765261263925 |
| O | -4.66712977195129 | 0.56532674067086 | 0.91970996852977 |
| H | -6.58419854087295 | 0.54906674201704 | 2.27923998566169 |
| H | -6.72679251502498 | -1.05432809579413 | 1.54695329370623 |
| C | -3.95619865223012 | -0.37989790901954 | 1.38410953878621 |
| C | -2.69252232100168 | -0.74910295749472 | 0.80489674705671 |
| C | -6.64537956115105 | -0.51899596193598 | 2.49639169529842 |
| H | -7.49016609593667 | -0.74366415739482 | 3.14461281765292 |
| C | -4.29535856698895 | -1.08618736579004 | 2.59545642075441 |
| C | -1.83853597197819 | -1.62446927828529 | 1.45548514861088 |
| O | -5.51330149213483 | -0.96001229162961 | 3.23076418519689 |
| H | -0.92519744050637 | -1.89371959209271 | 0.93698497660432 |
| C | -3.38242251390353 | -1.89242589062839 | 3.23831442487232 |
| C | -2.12605641096124 | -2.17050819116367 | 2.69803994867027 |
| H | -2.26917888794141 | -4.66877746669782 | 3.93036265338394 |
| H | -3.68124721854600 | -2.31804580291692 | 4.18513545306217 |
| O | -3.32528048591234 | -4.08691703482499 | 6.06304230007474 |
| C | -1.05461170501009 | -2.95829269079487 | 3.42174608900671 |

| | | | |
|---|---|---|---|
| O | 1.94942708394819 | -2.89803274013642 | 2.12797216191694 |
| H | -0.46939368807672 | -3.49957638486162 | 2.66301430305692 |
| C | -1.59328212096222 | -3.97391271181924 | 4.43227063892170 |
| H | 1.54765818281537 | -2.64511791820663 | 1.26883135939848 |
| C | -2.35106369917861 | -3.40895781263015 | 5.66864734298117 |
| H | 0.13317412743709 | -0.84511748207297 | 2.40768985260328 |
| H | 2.42741705289165 | -0.43404086768292 | 1.31425658724534 |
| C | 0.65543083815506 | -1.14943677998477 | 3.32850354536517 |
| O | -1.93334343395365 | -2.37386658758003 | 6.22812315035254 |
| C | 2.01808869808807 | -1.75894528735343 | 2.95278255890454 |
| N | -0.10344660972134 | -2.09250331401722 | 4.12885932268634 |
| H | -0.74154735075851 | -4.52998555557670 | 4.83021574801262 |
| C | 2.90195646587398 | -0.70022935192443 | 2.27695817150558 |
| H | 4.09781330109350 | -2.14935504294631 | 1.84311446500293 |
| O | 4.19373489804299 | -1.20749112628798 | 2.06623400202102 |
| H | -0.63435458136162 | -1.61739965465201 | 4.85916883545032 |
| H | 3.23061938317786 | 1.91461086489162 | 1.51844735404858 |
| H | 2.49686429321178 | -2.11412366716837 | 3.87407657938173 |
| O | -0.32809510482270 | 0.73619378105423 | 4.37757612285306 |
| C | 0.88982332735657 | 0.13042135607822 | 4.13973735974376 |
| O | 1.71051954665953 | 1.05655621341638 | 3.46486546127996 |
| C | 2.99900973692532 | 0.54930692793031 | 3.15289672958226 |
| C | 3.77520054976515 | 1.65964395279687 | 2.43746815150379 |
| H | 4.76174467767525 | 1.27175075199640 | 2.18232986331134 |
| H | -0.19076615335176 | 1.56346474152887 | 4.86927154704107 |
| H | 1.38547010834445 | -0.15377423839738 | 5.09336169790765 |
| H | 3.51121815454880 | 0.27834682012391 | 4.09031564121870 |
| O | 3.97542475414506 | 2.78539578574712 | 3.24502067267172 |
| H | 3.10963709917611 | 3.13227760297783 | 3.50124358313924 |
| N | 1.10036941878954 | -2.44184112165256 | -1.40234824575621 |
| O | 0.53443044175461 | -1.87689974381711 | -2.37963888393317 |
| O | 0.91272630780957 | -1.92279772486546 | -0.25473785020325 |

Structure of the intermolecular ferulic acid-glucosamine complex (XIII)c $NO_2^{\bullet}$

| | | | |
|---|---|---|---|
| O | 1.46379214129806 | 4.12698752668761 | -5.41295964635450 |
| O | 2.56900627647442 | 1.30888425450432 | -2.54713789092124 |
| O | 3.98903944463330 | 1.50970059655229 | -4.90417767561332 |
| O | -0.11624692326237 | 4.97549777647823 | -4.05065286199806 |
| O | 2.94808193940340 | 4.45416233646812 | -7.81400990618931 |
| N | 1.01625699767469 | 3.69206411200758 | -1.78357513268001 |
| C | 2.53009861867019 | 2.49293329260993 | -3.30359536925132 |
| C | 2.75116055713559 | 2.17116130097344 | -4.78929653193831 |
| C | 1.18437259557075 | 3.21155693248141 | -3.14818116347198 |
| C | 2.71757477282654 | 3.48280583510484 | -5.58725022425607 |
| C | 1.15419802750152 | 4.43454162176924 | -4.07453081146202 |
| C | 2.89460495656347 | 3.25376788711158 | -7.09333520635708 |
| H | 3.32569654036597 | 3.17100324355229 | -2.95314757339835 |
| H | 1.91412645981661 | 1.53497779998923 | -5.10718376955762 |
| H | 0.38191196542336 | 2.52395469160530 | -3.46367960393628 |
| H | 3.51714870553536 | 4.14296442187639 | -5.21387639355784 |
| H | 1.90294466527426 | 5.16218692394113 | -3.69248805305498 |
| H | 2.06733730657303 | 2.62224537897172 | -7.44194895503587 |
| H | 3.84932549632157 | 2.75676957088422 | -7.26866764371579 |
| H | 0.33352644382011 | 4.45090394600917 | -1.77477583077972 |
| H | 3.37579799776225 | 0.83544918259681 | -2.81871700823034 |
| H | 3.97606722682980 | 0.90010491716680 | -5.65495299979920 |
| H | -0.16196521112501 | 5.73019813736635 | -4.66187936028138 |
| H | 2.14154646697348 | 4.95458198279520 | -7.62888897825306 |
| O | -4.17865897908971 | 2.40332630996151 | -2.19532785165439 |
| O | -4.03089128558484 | -0.30936533509064 | -2.03793033908767 |
| O | -2.13625662770812 | 3.90704341474083 | 0.94109291231381 |
| O | -0.97270171190900 | 5.30731834393320 | -0.32305600626927 |
| C | -0.60062826451956 | 1.83804018618261 | -1.37957977570188 |

| | | | |
|---|---|---|---|
| C | -2.95540739893154 | 1.81984378081193 | -2.00977782040716 |
| C | -1.79395363404542 | 2.49904811241297 | -1.80237979330737 |
| C | -0.52418454913148 | 0.49660242051772 | -1.43300626910775 |
| C | -2.99859113647887 | 0.37918443357284 | -1.96108019093064 |
| C | -1.62875202459821 | -0.28893081902505 | -2.00923109554923 |
| C | 0.54466221381069 | 2.67636059036563 | -0.84828059996533 |
| C | 0.15308233279826 | 3.34648645864160 | 0.47850892696782 |
| C | -4.28300358621593 | 3.80125660296104 | -1.97046829969841 |
| C | -1.09129343330745 | 4.26041121661846 | 0.35063448008530 |
| H | -1.77891296399913 | 3.57504938085312 | -1.87107484110185 |
| H | 0.34331064197306 | -0.05052086168580 | -1.09877686337442 |
| H | 1.38737439983651 | 1.99966466560225 | -0.65951368142130 |
| H | -0.06831852160791 | 2.57708347272584 | 1.21936692494000 |
| H | -3.85924957641937 | 4.07350421293113 | -0.99841618706013 |
| H | -5.34998692162852 | 4.00688138354032 | -1.99251512161971 |
| H | -3.76597066551461 | 4.35883691454233 | -2.75644558878179 |
| H | 0.98907545340716 | 3.96413345245632 | 0.81000003643698 |
| O | -1.63392255902698 | -1.65259809085097 | -1.70572418607007 |
| H | -1.39021905480424 | -4.03486712243314 | -1.18848851773007 |
| H | -3.35851484735604 | 0.44408602268544 | 2.28097383729119 |
| H | -1.60062383775635 | 0.69917609932194 | 2.44476788373451 |
| C | -1.74101155792753 | -2.10728813633998 | -0.41006391030196 |
| C | -1.55360972801287 | -3.47953889868864 | -0.27928391892209 |
| C | -2.45195067961709 | 0.76850494136756 | 1.76001528033882 |
| H | -2.56013715826673 | 1.80164494460541 | 1.42433147918581 |
| C | -1.98554260340395 | -1.34264460735356 | 0.74080216787948 |
| C | -1.55916418865041 | -4.09469302114978 | 0.95677326557924 |
| O | -2.22950221975645 | 0.00669023938974 | 0.59596860131017 |
| H | -1.38550443778082 | -5.15572269785432 | 1.03472765899077 |
| C | -1.95200793687766 | -1.97581343767464 | 1.97557322670155 |
| C | -1.74011013631463 | -3.34046097959730 | 2.10715650820226 |
| H | -3.70211403985224 | -4.17462885926006 | 3.87473197740454 |
| H | -2.09254626874014 | -1.38398917920439 | 2.86394404275528 |
| O | -4.29326760860583 | -1.86357493874009 | 4.39354876931436 |
| C | -1.58801968787689 | -3.96390861330528 | 3.47987367777191 |
| O | 0.75244645640687 | -5.76135262314481 | 2.75293330092532 |
| H | -1.44411950974354 | -5.04301230033220 | 3.34141306256937 |
| C | -2.82377112028457 | -3.75333885119875 | 4.36571402334370 |
| H | 1.25862482434794 | -6.59225622773290 | 2.75878721696167 |
| C | -3.15407521118454 | -2.27510851142216 | 4.70295873440043 |
| H | 0.63316832684946 | -3.16850126565128 | 2.35205106937914 |
| H | 2.79255461091526 | -4.42817781501881 | 1.77569047623938 |
| C | 0.81609225556000 | -3.44164338123520 | 3.40529675133795 |
| O | -2.28384751447169 | -1.59613141692045 | 5.29737749216565 |
| C | 1.51246780663698 | -4.80925020898390 | 3.44944063589654 |
| N | -0.40416593111192 | -3.47999797616600 | 4.19447614359923 |
| H | -2.65047122592415 | -4.25828720826143 | 5.31782983288166 |
| C | 2.91673469040108 | -4.70944076212766 | 2.83011632456692 |
| H | 4.20200015241209 | -6.09341601881858 | 2.28746528467595 |
| O | 3.51878410943549 | -5.97361215096592 | 2.96177765231058 |
| H | -0.62487655841878 | -2.54600581316582 | 4.54087383322944 |
| H | 4.97939867021500 | -3.11087022356391 | 1.88471044378536 |
| H | 1.60729762287516 | -5.10658619085878 | 4.50526640587685 |
| O | 1.16867349378876 | -1.12806616649783 | 3.75766639828227 |
| C | 1.73551431001591 | -2.36750348800670 | 3.99875068015091 |
| O | 3.02071285426572 | -2.36500684355510 | 3.42492574147054 |
| C | 3.70810102648471 | -3.60413728627767 | 3.54558616233314 |
| C | 5.10172519886977 | -3.39502667932208 | 2.93761558844484 |
| H | 5.67255115048046 | -4.32091024653372 | 3.01646630589962 |
| H | 1.75525650501412 | -0.43482797651146 | 4.09880200212323 |
| H | 1.82797381529872 | -2.55740627340252 | 5.08929131090362 |
| H | 3.80072816896210 | -3.86335471769542 | 4.61197193071953 |
| O | 5.84771962904262 | -2.42942203524076 | 3.62904833114870 |

| | | | |
|---|---|---|---|
| H | 5.35291051483373 | -1.59894426366589 | 3.62236766659259 |
| N | -1.55372580844023 | -0.27214627287935 | -3.51920897145469 |
| O | -1.03437659268202 | 0.73675518225931 | -4.07699766090846 |
| O | -2.25678249487933 | -1.09395142624740 | -4.16454875606074 |

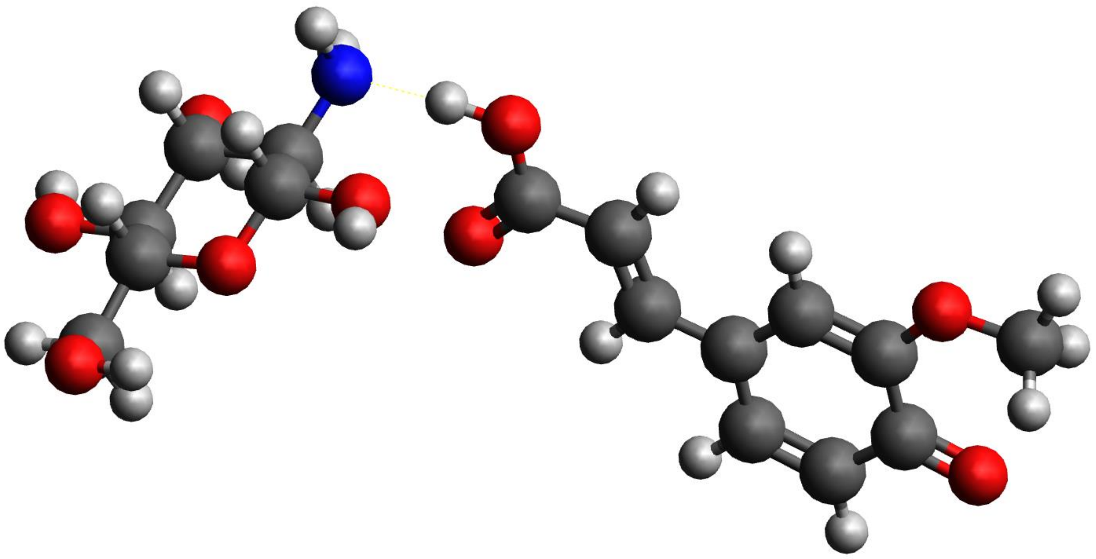

**Figure S1. The structure of the molecular complex of glucosamine and the ferulic acid radical, determined by the molecular docking method (GFN2-xTB quantum chemical modeling method).**

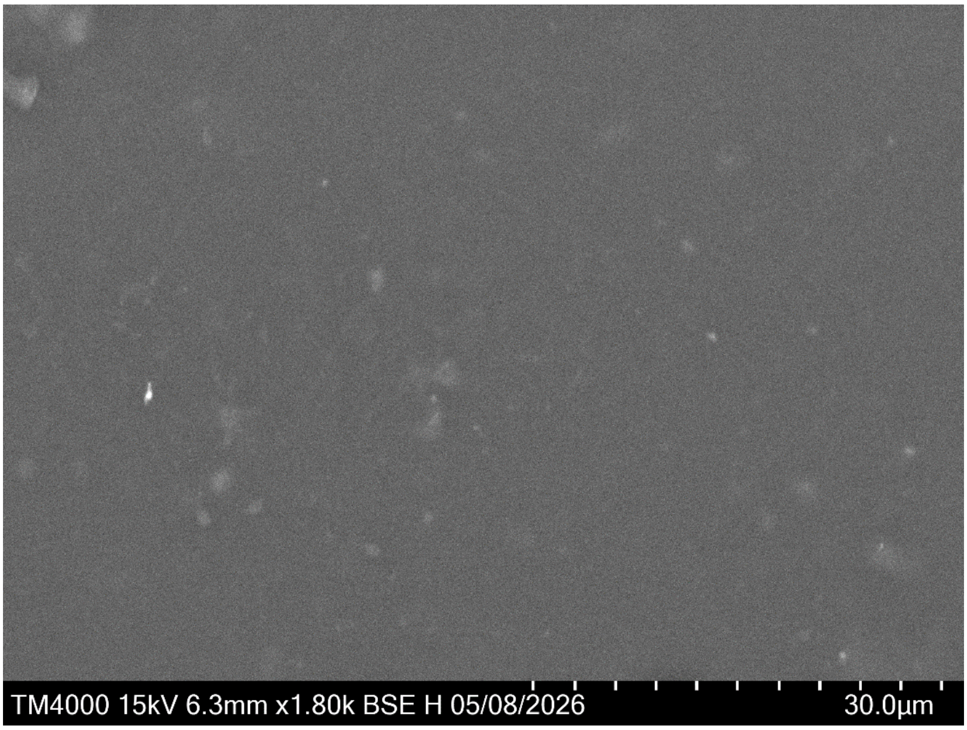


**Figure S2. Micrography of chitosan modified with ferulic acid in the presence of ScaSL laccase (130 U/g chitosan) at pH 7.0 with a ratio of n(FA)/n(NH2) = 1:10.**
